# Comparative genome-wide analysis of WRKY transcription factors in two Asian legume crops: Adzuki bean and Mung bean

**DOI:** 10.1038/s41598-018-34920-8

**Published:** 2018-11-19

**Authors:** Richa Srivastava, Sanjeev Kumar, Yasufumi Kobayashi, Kazutaka Kusunoki, Prateek Tripathi, Yuriko Kobayashi, Hiroyuki Koyama, Lingaraj Sahoo

**Affiliations:** 1 Department of Biosciences and Bioengineering, Indian Institute of Technology Guwahati, Guwahati, 781039 India; 2 Japan International Research Center for Agricultural Sciences Biological Resources, Post-Harvest Division, 1-1 Ohwashi, Tsukuba, Ibaraki, 305-8686 Japan; 3 Faculty of Applied Biological Sciences, Gifu University, 1-1, Yanagido, 501-1193 Gifu, Japan; 4 Department of Cell and Molecular Biology, The Scripps Research Institute, La Jolla, CA 92037 USA

## Abstract

The seminal participation of WRKY transcription factors in plant development, metabolism and in the governance of defense mechanism implicated their gaining importance for genomic and functional studies. The recent release of draft genome sequences of two legume crops, Adzuki bean (*Vigna angularis*) and Mung bean (*Vigna radiata*) has paved the way for characterization of WRKY gene family in these crops. We found 84 *WRKY* genes in Adzuki bean (*VaWRKY*) and 85 *WRKY* genes in Mung bean (*VrWRKY*). Based on the phylogenetic analysis, *VaWRKY* genes were classified into three groups with 15 members in Group I, 56 members in Group II, and 13 members in Group III, which was comparable to *VrWRKY* distribution in Mung bean, 16, 56 and 13 members in Group I, II and III, respectively. The few tandem and segmental duplication events suggested that recent duplication plays no prominent role in the expansion *VaWRKY* and *VrWRKY* genes. The illustration of gene-structure and their encoded protein-domains further revealed the nature of WRKY proteins. Moreover, the identification of abiotic or biotic stress-responsive *cis*-regulatory elements in the promoter regions of some *WRKY* genes provides fundamental insights for their further implementation in stress-tolerance and genetic improvement of agronomic traits.

## Introduction

Mung bean (*Vigna radiata*) is an annual, warm-season legume crop belonging to papilionoid subfamily of the Fabaceae. India is the leading producer of Mung bean in the world, followed by China and Myanmar^1^. It holds notable economic and health benefits. Mung bean seeds are a rich source of folate, iron and high-quality proteins^2^. It can revamp soil quality when grown in rotation with cereals^3^. Being a legume crop, intercropping of Mung bean with cereals have been reported to augment the crop yield as well as to diminish pest attacks^4,5^. Furthermore, genes of interest from wild species have been employed by plant breeders for crop improvement. For instance, resistance against bruchid beetles which cause considerable damage to the seeds during storage, was developed using a gene pool of a resistant Mung bean variety^6–8^. But, on the other hand, Mung bean is susceptible to the usual array of legume pathogens such as white mold, bacterial rots, *phytophthora* sp. and *Rhizoctonia* sp^9,10^.

Adzuki bean (*Vigna angularis*) is a close relative of Mung bean, cultivated widely in Japan, China, Korea and India^11^. It is the second most important legume in Japan, after soybean^12^. Moreover, it is mainly consumed as bean sprouts due to its high protein content (25%) in U.S. Although, the crop is somewhat drought tolerant, but it cannot tolerate water-logged soils as well as frost conditions. It is susceptible to stem rot caused by *Rhizoctonia solani* and, *Phytophthora* sp. and other bean diseases^12,13^. Due to its remarkable trait of drought-tolerance, Adzuki bean can be a good candidate to be explored for the gene network and control mechanism involved in the drought-resistance and exploiting for agronomical benefits. On the other hand, improved varieties are required to combat against various biotic and abiotic stresses influencing the crop cultivation.

Regardless of the immense economic importance of the above mentioned legume crops, the genomic studies of these crops are still inadequate. The sole reason behind was the unavailability of the full sequenced genome. The recent release of the draft of whole genome sequence of *Vigna* *angularis* and *Vigna* *radiata* in 2014 and 2015^14,15^, respectively, has paved the way for the genomic and functional studies of these crops.

Plants employ diverse approaches to acclimatize to environmental cues. Tuning the expression of genes deploying transcriptional regulators in respond to disparate environmental and physiological stimuli is one of the ways employed by eukaryotic organisms^16,17^. Plants dedicate approximately 7% of its genome to encode vital transcriptional regulators called transcription factors (TF), which facilitates sequence-specific binding to candidate genes through their conserved DNA-binding domains^18^. Adzuki bean genome and Mung bean genome allocates 2269 genes and 1850 genes encoding transcription factors, respectively^14,15^. Despite of their significance, none of the transcription factor family in these legumes crops has been explored yet.

WRKY transcription factor family is one such family known to endow their contribution in diverse biological phenomena in plants including defense responses^19^. Initially WRKY TFs were thought to be involved particularly, in plant-pathogen interaction, but recent functional studies implicate their gaining importance in abiotic stress responses as well^20^. WRKY TFs can act as positive as well as negative regulators of stress responses. For instance, one of the soy bean (*Glycine max*) WRKY genes, *GmWRKY21* impart tolerance against cold stress in *Arabidopsis thaliana*, while *GmWRKY54* confers salt and drought tolerance^21^. In rice (*Oryza sativa*), *OsWRKY72* confers tolerance towards high salt and drought via ABA signaling^22^. *OsWRKY8* provides osmotic stress tolerance^23^. *OsWRKY11* induced by heat provides heat and drought tolerance^24^. *AtWRKY26* is activated by a heat-induced ethylene-dependent response imparts heat resistance^25^. On the other hand, some WRKY proteins confer sensitivity in plants towards stress. Like, *AtWRKY2* induced by NaCl and mannitol and *AtWRKY40* induced by ABA have been proposed to act as a negative regulator of ABA-induced seed dormancy^26,27^. Similarly, overexpression of *AtWRKY18* and *AtWRKY60* make the plant more susceptible towards salt and osmotic stresses^28^. Moreover, a crosstalk between biotic and abiotic stress response components is reported to exist. Apart from its previously mentioned role, *AtWRKY18* is induced by ABA as well as salicylic acid and pathogens^26,29^. *OsWRKY89* induced by salinity, ABA and UV-B and also confers disease resistance^26,30^. *AtWRKY53* which is a negative regulator drought tolerance, involved in senescence, also plays role in basal defense in plants^31–33^. *AtWRKY7*0 is involved in disease resistance, basal defense as well as in senescence^34,35^. *OsWRKY45* from rice enhances disease resistance, but also provides drought and cold tolerance in *Arabidopsis*^36,37^.

The WRKY family proteins contains one or two highly conserved domains of around 60 amino acids, comprising of a hallmark heptapeptide WRKYGQK at N-terminal, and a signature C-terminal zinc-finger motif^38,39^. They bind to the W box and a sugar-responsive *cis*-element SURE in the promoter of the target gene^40^. All known members of WRKY family can be classified into three groups viz. group I, II, and III, based on the number of occurrence of WRKY domains and the types of zinc finger motif. In the group I proteins, two WRKY domains can be found, whereas the group II and the group III possess only a single domain^38,39^. Group II is further divided into several subgroups based on the phylogenetic clades^19^.

In this study, we conducted a genome-wide survey to identify and designate nomenclature to WRKY TF family in Adzuki bean (VaWRKY) and Mung bean (VrWRKY) by implementing bioinformatics approach on the publicly available database of sequenced genome. We identified a comprehensive and non-redundant set of 84 *VaWRKY* genes and 85 *VrWRKY* genes encoding the WRKY transcription factor family in Adzuki bean and Mung bean, respectively and manually curated them. Subsequently, gene classification, exon–intron organization, chromosome distribution, gene duplication events, phylogenetic relationships, and conserved motifs were also studied, which lay a firm foundation for further comparative genomics studies. We also carried out promoter analysis to investigate stress-responsive *cis*-regulatory elements in 17 *VaWRKY* genes and 18 *VrWRKY* genes orthologous to strongly reported stress-responsive WRKY proteins of *Arabidopsis*, Rice and Soybean. Our results may provide a subset of potential candidates to be explored for stress-tolerance and genetic improvement of agronomic traits in Adzuki bean and Mung bean, as well as other related crops.

## Results

### Identification and classification of VaWRKY and VrWRKY protein family based on the WRKY domain

In this study, 91 proteins in Adzuki bean were identified possessing WRKY DNA-binding domain, whereas a total of 85 WRKY proteins were found in Mung bean (Supplementary Table I). Out of these, 78 VaWRKY and 68 VrWRKY proteins contain full-length WRKY domains of around 60 amino acids, while the remaining members possess partial WRKY domains. In case of VaWRKY proteins, six members have truncated N-terminal WRKY domain, while seven members have truncated C- terminal WRKY domain. Whereas, in case of VrWRKY proteins, six members lack intact N-terminal WRKY domain, whereas eleven members lack full length C- terminal WRKY domain (Table I). But as these proteins significantly possess typical conserved sequences, they were retained for their further analysis considering their putative functional roles. In case of Adzuki bean, seven proteins were isoforms, but no isoforms for WRKY proteins were observed in Mung bean. In total, 84 non-redundant *VaWRKY* genes and 85 *VrWRKY* genes were identified encoding the WRKY family of Adzuki bean and Mung bean, respectively. Although the presence partial proteins suggested the occurrence of pseudogenes encoding truncated WRKY protein. In total, 84 non-redundant putative *VaWRKY* genes and 85 putative *VrWRKY* genes were identified encoding the WRKY family of Adzuki bean and Mung bean, respectively. They were designated as *VaWRKY1-84* and *VrWRKY1-85*, representing the WRKY members of Adzuki bean and Mung bean, respectively in Table I.

The length of the proteins along with their theoretical pI (isoelectric point) and molecular weight were indicated in Supplementary Table I. The pI value ranged from 4.96 (VaWRKY73) to 9.99 (VaWRKY57) in Adzuki bean, and from 4.74 (VrWRKY55) to 9.81 (VrWRKY57) in Mung bean. Also, the average length of VaWRKY and VrWRKY proteins was found approximately 340 amino acid residues, with the longest protein of 746 amino acid residues (VrWRKY7).

**Figure 1:**
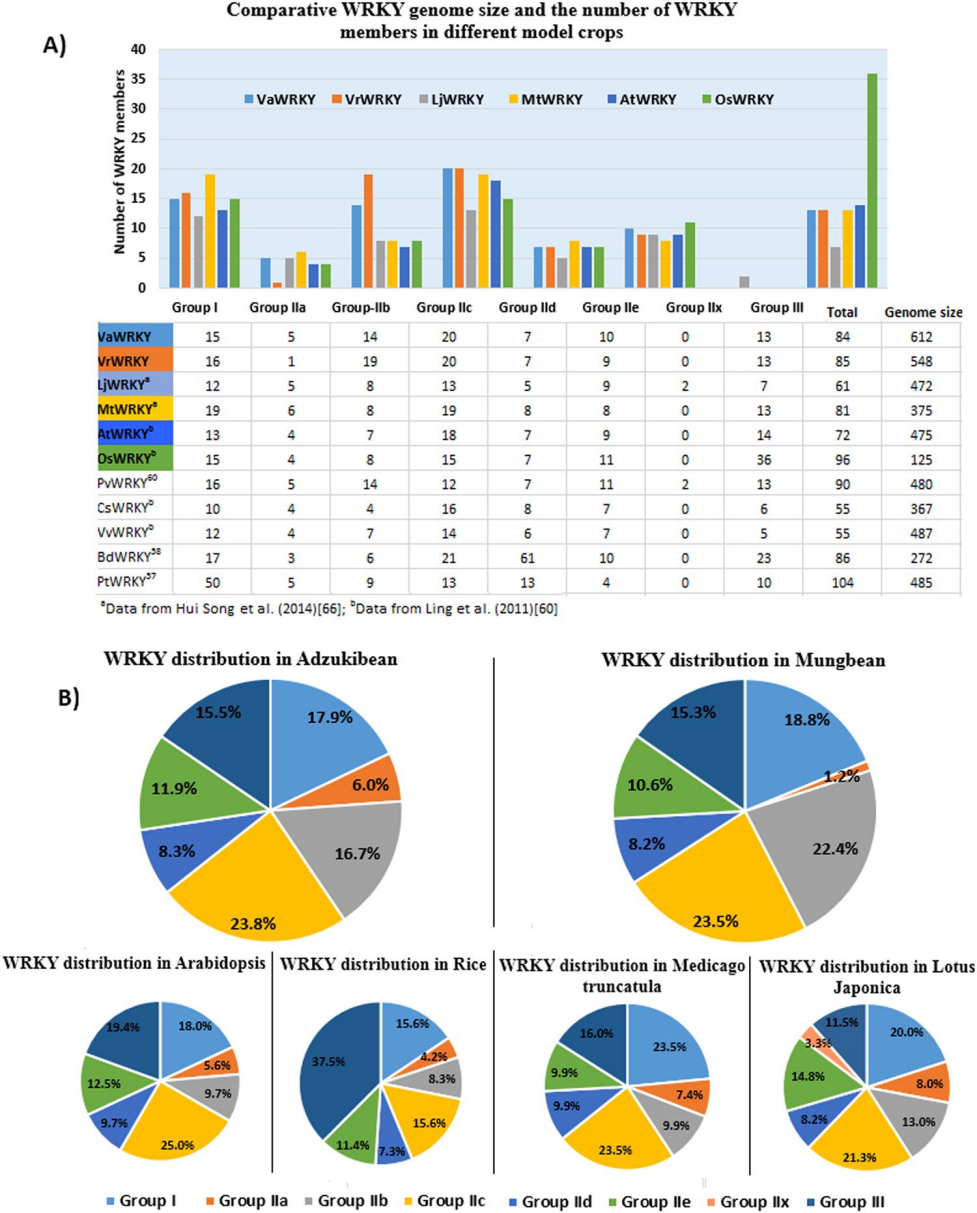
(**A**) Number *WRKY* genes in Adzuki bean, Mung bean and various model legume crops and *Arabidopsis*, (**B**) Percentage distribution of *WRKY* members in Adzuki bean, Mung bean, *Arabidopsis* and various model legume crops.

Generally, the WRKY proteins are classified in three groups and 5 sub-groups, depending on the number and nature of the WRKY domain, they possess. The members possessing two WRKY domains at N-terminal and C-terminal (Group-I NTWD and Group-I CTWD), were grouped into Group I, while the members having only one WRKY domain belonged to Group II and Group III. In order to further classify the WRKY proteins, we analyzed multiple sequence alignment of domain sequences, and constructed a phylogenetic of VaWRKY and VrWRKY proteins, with WRKY sequences of a model legume crop, *L*. *japonica*, as reference, representing each group and subgroup. Moreover, a phylogenetic tree of WRKY domains, excluding the members with truncated domains was also constructed for further supporting the classification (Supplementary Fig. S2). The phylogenies of the domains also indicate the consequence of the evolutionary selection and suggest their origins. As per our findings, the VaWRKY protein family have 17 members in Group I; five members in Group IIa, fourteen members in Group IIb, 24 members in Group IIc, seven members in Group IId and eleven members in Group IIe; and thirteen members in Group III (Table 1). The distribution of genes encoding these 91 proteins, considering seven isoforms were as follows: fifteen members in Group I; five members in Group Ia, fourteen members in Group IIb, twenty members in Group IIc, seven members in Group IId and ten members in Group IIe; and thirteen members in Group III (Fig. 1A). While in Mung bean, where no isoforms were observed, we found sixteen genes in Group I; 1 gene in Group Ia, nineteen genes in Group IIb, twenty genes in Group IIc, seven genes in Group IId and nine genes in Group IIe; and thirteen genes encoding VrWRKY proteins in Group III. The distribution of *WRKY* genes in Mung bean was comparable to that of Adzuki bean (Fig. 1B). The distribution of Group I, Group IIc, Group IId, Group IIe and Group III was similar in Adzuki bean and Mung bean (17.9% and 18.8%, 23.8% and 23.5%, 8.3% and 8.2%, 11.9% and 10.6% and 15.5% and 15.3%, respectively). Although the numbers of Group IIa and Group IIb members differs between Adzuki bean and Mung bean, the combined composition of Group IIa and Group IIb was similar in both the crops, which is approximately 23%.

**Table 1:** Classification of VaWRKY and VrWRKY proteins.

Protein name	Protein ID	WRKY Domain			No. of domains	Group	Protein name	Protein ID	WRKY Domain			No. of domains	Group
		Conserved heptapeptide	Zinc-finger	Zinc-finger type					Conserved heptapeptide	Zinc-finger	Zinc-finger type		
VaWRKY1	Vang09g01780.1	WRKYGQK/WRKYGQK	C_2_H_2_/C_2_H_2_	CX_4_CX_22_HXH/CX_4_CX_23_HXH	2	I	VrWRKY1	Vradi03g09710.1	WRKYGQK/WRKYGQK	C_2_H_2_/C_2_H_2_	CX_4_CX_22_HXH/CX_4_CX_23_HXH	2	I
VaWRKY2	Vang11g11810.1	WRKYGQK/WRKYGQK	C_2_H_2_/–	CX_4_CX_22_HXH/ CX_4_CX_23_	2	I	VrWRKY2	Vradi07g21330.1	WRKYGQK/WRKYGQK	C_2_H_2_/–	CX_4_CX_22_HXH/–	2	I
VaWRKY3	Vang06g11490.1	WRKYGQK/WRKYGQK	–/C_2_H_2_	CX_4_CX_23_ /CX_4_CX_23_HXH	2	I	VrWRKY3	Vradi05g10960.1	WRKYGQK/WKKYGQK	C_2_H_2_/C_2_H_2_	CX_5_CX_23_HXH/CX_5_CX_23_HXH	2	I
VaWRKY4	Vang0103s00010.1	WRKYGEK/WRKYGQK	C_2_H_2_/C_2_H_2_	CX_4_CX_22_HXH/CX_4_CX_23_HXH	2	I	VrWRKY4	Vradi08g13900.1	WRKYGQK/WRKYGQK	–/C_2_H_2_	–/CX_4_CX_23_HXH	2	I
VaWRKY5	Vang04g08110.1	WRKYGQK/WRKYGQK	C_2_H_2_/C_2_H_2_	CX_4_CX_22_HXH/CX_4_CX_23_HXH	2	I	VrWRKY5	Vradi06g01200.1	WRKYGQK/WRKYGQK	C_2_H_2_/C_2_H_2_	CX_4_CX_22_HXH/CX_4_CX_23_HXH	2	I
VaWRKY6	Vang03g12780.1	WRKYGQK/–	C_2_H_2_/C_2_H_2_	CX_4_CX_22_HXH/CX_4_CX_23_HXH	2	I	VrWRKY6	Vradi10g02560.1	WRKYGQK/WRKYGQK	C_2_H_2_/C_2_H_2_	CX_4_CX_22_HXH/CX_4_CX_23_HXH	2	I
VaWRKY7	Vang01g03800.1	WRKYGQK/WRKYGQK	C_2_H_2_/C_2_H_2_	CX_4_CX_22_HXH/CX_4_CX_23_HXH	2	I	VrWRKY7	Vradi05g21980.1	WRKYGQK/WRKYGQK	C_2_H_2_/C_2_H_2_	CX_4_CX_22_HXH/CX_4_CX_23_HXH	2	I
VaWRKY8	Vang04g14650.1	WRKYGQK/WRKYGQK	C_2_H_2_/C_2_H_2_	CX_4_CX_22_HXH/CX_4_CX_23_HXH	2	I	VrWRKY8	Vradi07g06660.1	WRKYGQK/WRKYGQK	C_2_H_2_/C_2_H_2_	CX_4_CX_22_HXH/CX_4_CX_23_HXH	2	I
VaWRKY9	Vang04g04330.1	WRKYGQK/WRKYGQK	C_2_H_2_/C_2_H_2_	CX_4_CX_22_HXH/CX_4_CX_23_HXH	2	I	VrWRKY9	Vradi0100s00250.1	WRKYGQK/WRKYGQK	–/C_2_H_2_	–/CX_4_CX_23_HXH	2	I
VaWRKY10	Vang01g01490.1	WRKYGQK/WRKYGQK	C_2_H_2_/C_2_H_2_	CX_4_CX_22_HXH/CX_4_CX_23_HXH	2	I	VrWRKY10	Vradi04g09020.1	WRKYGQK/WRKYGQK	C_2_H_2_/C_2_H_2_	CX_4_CX_22_HXH/CX_4_CX_23_HXH	2	I
VaWRKY11	Vang03g14810.1	WRKYGQK/WRKYGQK	C_2_H_2_/C_2_H_2_	CX_4_CX_22_HXH/CX_4_CX_23_HXH	2	I	VrWRKY11	Vradi06g06770.1	WRKYGQK/WRKYGQK	C_2_H_2_/C_2_H_2_	CX_4_CX_22_HXH/CX_4_CX_23_HXH	2	I
VaWRKY12	Vang0039ss00390.1	WRKYGQK/WRKYGQK	C_2_H_2_/C_2_H_2_	CX_4_CX_22_HXH/CX_4_CX_23_HXH	2	I	VrWRKY12	Vradi0158s00480.1	WRKYGQK/WRKYGQK	C_2_H_2_/C_2_H_2_	CX_4_CX_22_HXH/CX_4_CX_23_HXH	2	I
VaWRKY12	Vang0039ss00390.2	WRKYGQK/WRKYGQK	C_2_H_2_/C_2_H_2_	CX_4_CX_22_HXH/CX_4_CX_23_HXH	2	I	VrWRKY13	Vradi0261s00010.1	WRKYGEK/WRKYGQK	C_2_H_2_/C_2_H_2_	CX_4_CX_22_HXH/CX_4_CX_23_HXH	2	I
VaWRKY13	Vang0397s00030.1	WRKYGQK/WRKYGQK	C_2_H_2_/C_2_H_2_	CX_4_CX_22_HXH/CX_4_CX_23_HXH	2	I	VrWRKY14	Vradi0417s00050.1	WRKYGQK/WRKYGQK	C_2_H_2_/C_2_H_2_	CX_4_CX_22_HXH/CX_4_CX_23_HXH	2	I
VaWRKY14	Vang04g00270.1	WRKYGQK/WRKYGQK	C_2_H_2_/C_2_H_2_	CX_4_CX_22_HXH/CX_4_CX_23_HXH	2	I	VrWRKY15	Vradi03g03190.1	WRKYGQK/WRKYGQK	C_2_H_2_/C_2_H_2_	CX_4_CX_22_HXH/CX_4_CX_23_HXH	2	I
VaWRKY15	Vang01g00540.1	WRKYGQK/WRKYGQK	C_2_H_2_/C_2_H_2_	CX_4_CX_22_HXH/CX_4_CX_23_HXH	2	I	VrWRKY16	Vradi06g00500.1	WRKYGQK/WRKYGQK	C_2_H_2_/C_2_H_2_	CX_4_CX_22_HXH/CX_4_CX_23_HXH	2	I
VaWRKY15	Vang01g00540.2	WRKYGQK/WRKYGQK	C_2_H_2_/C_2_H_2_	CX_4_CX_22_HXH/CX_4_CX_23_HXH	2	I	VrWRKY17	Vradi06g13520.1	WRKYGQK	C_2_H_2_	CX_5_CX_23_HXH	1	IIa
VaWRKY16	Vang0027ss00340.1	WRKYGQK	C_2_H_2_	CX_5_CX_23_HXH	1	IIa	VrWRKY18	Vradi07g15410.1	WRKYGQK	C_2_H_2_	CX_5_CX_23_HXH	1	IIb
VaWRKY17	Vang01g15570.1	—	C_2_H_2_	CX_5_CX_23_HXH	1	IIa	VrWRKY19	Vradi0349s00020.1	WRKYGQK	C_2_H_2_	CX_5_CX_23_HXH	1	IIb
VaWRKY18	Vang05g09440.1	WRKYGQK	C_2_H_2_	CX_5_CX_23_HXH	1	IIa	VrWRKY20	Vradi11g10580.1	WRKYGQK	C_2_H_2_	CX_5_CX_23_HXH	1	IIb
VaWRKY19	Vang01g15560.1	WRKYGQK	C_2_H_2_	CX_5_CX_23_HXH	1	IIa	VrWRKY21	Vradi08g16000.1	WRKYGQK	C_2_H_2_	CX_5_CX_23_HXH	1	IIb
VaWRKY20	Vang05g09490.1	WRKYGQK	C_2_H_2_	CX_5_CX_23_HXH	1	IIa	VrWRKY22	Vradi06g11150.1	WRKYGQK	C_2_H_2_	CX_5_CX_23_HXH	1	IIb
VaWRKY21	Vang0033ss01130.1	WRKYGQK	C_2_H_2_	CX_5_CX_23_HXH	1	IIb	VrWRKY23	Vradi06g07040.1	WRKYGQK	C_2_H_2_	CX_5_CX_23_HXH	1	IIb
VaWRKY22	Vang0065s00590.1	WRKYGQK	—	CX_5_CX_23_	1	IIb	VrWRKY24	Vradi07g05680.1	WRKYGQK	C_2_H_2_	CX_5_CX_23_HXH	1	IIb
VaWRKY23	Vang1037s00010.1	WRKYGQK	C_2_H_2_	CX_5_CX_30_HXH	1	IIb	VrWRKY25	Vradi0401s00040.1	WRKYGQK	C_2_H_2_	CX_5_CX_23_HXH	1	IIb
VaWRKY24	Vang04g05450.1	WRKYGQK	C_2_H_2_	CX_5_CX_23_HXH	1	IIb	VrWRKY26	Vradi07g23970.1	WRKYGQK	C_2_H_2_	CX_5_CX_23_HXH	1	IIb
VaWRKY25	Vang11g15810.1	WRKYGQK	C_2_H_2_	CX_5_CX_23_HXH	1	IIb	VrWRKY27	Vradi0335s00020.1	WRKYGQK	C_2_H_2_	CX_5_CX_23_HXH	1	IIb
VaWRKY26	Vang09g05580.1	WRKYGQK	C_2_H_2_	CX_5_CX_23_HXH	1	IIb	VrWRKY28	Vradi06g01560.1	WRKYGQK	C_2_H_2_	CX_5_CX_23_HXH	1	IIb
VaWRKY27	Vang0318s00160.1	WRKYGQK	C_2_H_2_	CX_5_CX_23_HXH	1	IIb	VrWRKY29	Vradi0222s00030.1	—	C_2_H_2_	CX_5_CX_23_HXH	1	IIb
VaWRKY28	Vang01g02960.1	WRKYGQK	C_2_H_2_	CX_5_CX_23_HXH	1	IIb	VrWRKY30	Vradi05g22430.1	WRKYGQK	C_2_H_2_	CX_5_CX_23_HXH	1	IIb
VaWRKY29	Vang03g07430.1	WRKYGQK	C_2_H_2_	CX_5_CX_23_HXH	1	IIb	VrWRKY31	Vradi02g07100.1	WRKYGQK	C_2_H_2_	CX_5_CX_23_HXH	1	IIb
VaWRKY30	Vang0032ss02430.1	WRKYGQK	C_2_H_2_	CX_5_CX_23_HXH	1	IIb	VrWRKY32	Vradi0048s00100.1	WRKYGQK	C_2_H_2_	CX_5_CX_23_HXH	1	IIb
VaWRKY31	Vang09g01900.1	WRKYGQK	C_2_H_2_	CX_5_CX_23_HXH	1	IIb	VrWRKY33	Vradi04g08580.1	WRKYGQK	C_2_H_2_	CX_5_CX_23_HXH	1	IIb
VaWRKY32	Vang04g17360.1	WRKYGQK	C_2_H_2_	CX_5_CX_23_HXH	1	IIb	VrWRKY34	Vradi01g11520.1	WRKYGQK	C_2_H_2_	CX_5_CX_23_HXH	1	IIb
VaWRKY33	Vang06g15530.1	WRKYGQK	C_2_H_2_	CX_5_CX_23_HXH	1	IIb	VrWRKY35	Vradi0273s00140.1	WRKYGQK	C_2_H_2_	CX_5_CX_23_HXH	1	IIb
VaWRKY34	Vang0322s00110.1	WRKYGQK	C_2_H_2_	CX_5_CX_23_HXH	1	IIb	VrWRKY36	Vradi0111s00350.1	WRKYGQK	—	—	1	IIb
VaWRKY35	Vang0051s00140.1	WRKYGQK	C_2_H_2_	CX_4_CX_23_HXH	1	IIc	VrWRKY37	Vradi07g24510.1	—	C_2_H_2_	CX_4_CX_23_HXH	1	IIc
VaWRKY36	Vang08g01570.1	SRKYGQK	C_2_H_2_	CX_4_CX_22_HXH	1	IIc	VrWRKY38	Vradi0043s00750.1	WRKYGKK	C_2_H_2_	CX_4_CX_23_HXH	1	IIc
VaWRKY37	Vang04g03920.1	WRKYGQK	C_2_H_2_	CX_4_CX_23_HXH	1	IIc	VrWRKY39	Vradi07g30190.1	—	C_2_H_2_	CX_4_CX_23_HXH	1	IIc
VaWRKY38	Vang08g00900.1	WRKYGQK	C_2_H_2_	CX_4_CX_23_HXH	1	IIc	VrWRKY40	Vradi05g05410.1	WRKYGKK	C_2_H_2_	CX_4_CX_23_HXH	1	IIc
VaWRKY39	Vang04g12730.1	WRKYGQK	C_2_H_2_	CX_4_CX_23_HXH	1	IIc	VrWRKY41	Vradi06g07670.1	—	C_2_H_2_	CX_4_CX_23_HXH	1	IIc
VaWRKY40	Vang0173s00160.1	WRKYGKK	C_2_H_2_	CX_4_CX_22_HXH	1	IIc	VrWRKY42	Vradi04g07740.1	WRKYGQK	C_2_H_2_	CX_4_CX_23_HXH	1	IIc
VaWRKY41	Vang10g07150.1	WRKYGEK	C_2_H_2_	CX_4_CX_23_HXH	1	IIc	VrWRKY43	Vradi10g06370.1	WRKYGQK	—	—	1	IIc
VaWRKY42	Vang0005s00450.1	WRKYGQK	C_2_H_2_	CX_4_CX_23_HXH	1	IIc	VrWRKY44	Vradi09g05960.1	WRKYGQK	—	—	1	IIc
VaWRKY43	Vang05g03980.1	WRKYGKK	—	—	1	IIc	VrWRKY45	Vradi01g10680.1	WRKYGQK	C_2_H_2_	CX_4_CX_23_HXH	1	IIc
VaWRKY44	Vang01g17410.1	WRKYGKK	C_2_H_2_	CX_4_CX_23_HXH	1	IIc	VrWRKY46	Vradi0043s00400.1	WRKYGQK	C_2_H_2_	CX_4_CX_23_HXH	1	IIc
VaWRKY45	Vang0333s00130.1	WRKYGQK	C_2_H_2_	CX_4_CX_23_HXH	1	IIc	VrWRKY47	Vradi03g06620.1	WRKYGQK	C_2_H_2_	CX_4_CX_23_HXH	1	IIc
VaWRKY46	Vang01g02180.1	WRKYGQK	C_2_H_2_	CX_4_CX_23_HXH	1	IIc	VrWRKY48	Vradi0100s00500.1	WRKYGQK	C_2_H_2_	CX_4_CX_23_HXH	1	IIc
VaWRKY47	Vang0005s00190.1	WRKYGQK	C_2_H_2_	CX_4_CX_23_HXH	1	IIc	VrWRKY49	Vradi0048s00470.1	WRKYGQK	C_2_H_2_	CX_4_CX_23_HXH	1	IIc
VaWRKY48	Vang04g17060.1	WRKYGQK	C_2_H_2_	CX_4_CX_23_HXH	1	IIc	VrWRKY50	Vradi04g07130.1	WRKYGQK	C_2_H_2_	CX_4_CX_23_HXH	1	IIc
VaWRKY49	Vang11g16350.1	WRKYGQK	C_2_H_2_	CX_4_CX_23_HXH	1	IIc	VrWRKY51	Vradi09g03960.1	WRKYGQK	C_2_H_2_	CX_4_CX_23_HXH	1	IIc
VaWRKY50	Vang10g04840.1	WRKYGQK	C_2_H_2_	CX_4_CX_23_HXH	1	IIc	VrWRKY52	Vradi0146s00260.1	WRKYGQK	C_2_H_2_	CX_4_CX_23_HXH	1	IIc
VaWRKY51	Vang0942s00010.1	WRKYGQK	C_2_H_2_	CX_4_CX_23_HXH	1	IIc	VrWRKY53	Vradi06g02270.1	WRKYGQK	C_2_H_2_	CX_4_CX_23_HXH	1	IIc
VaWRKY51	Vang0942s00010.2	WRKYGQK	C_2_H_2_	CX_4_CX_23_HXH	1	IIc	VrWRKY54	Vradi01g10590.1	WRKYGKK	C_2_H_2_	CX_4_CX_23_HXH	1	IIc
VaWRKY52	Vang08g06450.1	WRKYGQK	C_2_H_2_	CX_4_CX_23_HXH	1	IIc	VrWRKY55	Vradi06g13730.1	WRKYGKK	—	—	1	IIc
VaWRKY52	Vang08g06450.2	WRKYGQK	C_2_H_2_	CX_4_CX_23_HXH	1	IIc	VrWRKY56	Vradi05g11580.1	WRKYGKK	—	CX_4_CX_23_	1	IIc
VaWRKY52	Vang08g06450.3	WRKYGQK	C_2_H_2_	CX_4_CX_23_HXH	1	IIc	VrWRKY57	Vradi08g08840.1	WRKYGQK	C_2_H_2_	CX_5_CX_23_HXH	1	IId
VaWRKY52	Vang08g06450.4	WRKYGQK	C_2_H_2_	CX_4_CX_23_HXH	1	IIc	VrWRKY58	Vradi11g04520.1	WRKYGQK	C_2_H_2_	CX_5_CX_23_HXH	1	IId
VaWRKY53	Vang10g03000.1	WRKYGKK	C_2_H_2_	CX_4_CX_23_HXH	1	IIc	VrWRKY59	Vradi07g10760.1	WRKYGQK	C_2_H_2_	CX_5_CX_23_HXH	1	IId
VaWRKY54	Vang0605s00070.1	WRKYGKK	C_2_H_2_	CX_4_CX_23_HXH	1	IIc	VrWRKY60	Vradi07g22750.1	WRKYGQK	C_2_H_2_	CX_5_CX_23_HXH	1	IId
VaWRKY55	Vang07g02340.1	WRKYGQK	C_2_H_2_	CX_5_CX_23_HXH	1	IId	VrWRKY61	Vradi05g02540.1	WRKYGQK	C_2_H_2_	CX_5_CX_23_HXH	1	IId
VaWRKY56	Vang11g04110.1	WRKYGQK	C_2_H_2_	CX_5_CX_23_HXH	1	IId	VrWRKY62	Vradi06g12190.1	WRKYGQK	C_2_H_2_	CX_5_CX_23_HXH	1	IId
VaWRKY57	Vang11g13040.1	WRKYGQK	C_2_H_2_	CX_5_CX_23_HXH	1	IId	VrWRKY63	Vradi05g09450.1	WRKYGQK	C_2_H_2_	CX_5_CX_23_HXH	1	IId
VaWRKY58	Vang09g06970.1	WRKYGQK	C_2_H_2_	CX_5_CX_23_HXH	1	IId	VrWRKY64	Vradi01g14060.1	WRKYGQK	C_2_H_2_	CX_5_CX_23_HXH	1	IIe
VaWRKY59	Vang05g08140.1	WRKYGQK	C_2_H_2_	CX_5_CX_23_HXH	1	IId	VrWRKY65	Vradi04g07100.1	—	C_2_H_2_	CX_5_CX_23_HXH	1	IIe
VaWRKY60	Vang01g12760.1	WRKYGQK	C_2_H_2_	CX_5_CX_23_HXH	1	IId	VrWRKY66	Vradi09g04070.1	WRKYGQK	C_2_H_2_	CX_5_CX_23_HXH	1	IIe
VaWRKY61	Vang06g17510.1	WRKYGQK	C_2_H_2_	CX_5_CX_23_HXH	1	IId	VrWRKY67	Vradi0048s00350.1	WRKYGQK	—	CX_5_CX_23_	1	IIe
VaWRKY62	Vang0013ss00970.1	WRKYGQK	—	—	1	IIe	VrWRKY68	Vradi10g06090.1	WRKYGQK	C_2_H_2_	CX_5_CX_23_HXH	1	IIe
VaWRKY62	Vang0013ss00970.2	WRKYGQK	—	—	1	IIe	VrWRKY69	Vradi07g30880.1	WRKYGQK	C_2_H_2_	CX_5_CX_23_HXH	1	IIe
VaWRKY63	Vang0304s00120.1	WRKYGQK	C_2_H_2_	CX_5_CX_23_HXH	1	IIe	VrWRKY70	Vradi0023s00350.1	WRKYGQK	—	CX_5_CX_23_	1	IIe
VaWRKY64	Vang07g06530.1	WRKYGQK	C_2_H_2_	CX_5_CX_23_HXH	1	IIe	VrWRKY71	Vradi0161s00550.1	WRKYGQK	—	—	1	IIe
VaWRKY65	Vang10g06430.1	WRKYGQK	C_2_H_2_	CX_5_CX_23_HXH	1	IIe	VrWRKY72	Vradi03g06560.1	WRKYGQK	C_2_H_2_	CX_5_CX_23_HXH	1	IIe
VaWRKY66	Vang06g08640.1	WRKYGQK	—	—	1	IIe	VrWRKY73	Vradi11g01720.1	WRKYGQK	C_2_HC	CX_7_CX_23_HXC	1	III
VaWRKY67	Vang02g05830.1	WRKYGQK	C_2_H_2_	CX_5_CX_23_HXH	1	IIe	VrWRKY74	Vradi07g29640.1	WRKYGQK	C_2_HC	CX_7_CX_23_HXC	1	III
VaWRKY68	Vang08g01000.1	WRKYGQK	C_2_H_2_	CX_5_CX_23_HXH	1	IIe	VrWRKY75	Vradi0183s00040.1	WRKYGQK	C_2_HC	CX_7_CX_23_HXC	1	III
VaWRKY69	Vang0322s00040.1	WRKYGQK	C_2_H_2_	CX_5_CX_23_HXH	1	IIe	VrWRKY76	Vradi0083s00100.1	WRKYGQK	C_2_HC	CX_7_CX_23_HXC	1	III
VaWRKY70	Vang03g08430.1	WRKYGQK	C_2_H_2_	CX_5_CX_23_HXH	1	IIe	VrWRKY77	Vradi09g03480.1	WRKYGQK	C_2_HC	CX_7_CX_23_HXC	1	III
VaWRKY71	Vang04g16950.1	WRKYGQK	C_2_H_2_	CX_5_CX_23_HXH	1	IIe	VrWRKY78	Vradi05g05160.1	WRKYGQK	C_2_HC	CX_7_CX_23_HXC	1	III
VaWRKY72	Vang0228s00230.1	WRKYGQK	C_2_HC	CX_7_CX_23_HXC	1	III	VrWRKY79	Vradi0214s00230.1	WRKYGQK	C_2_HC	CX_7_CX_23_HXC	1	III
VaWRKY73	Vang08g00330.1	WRKYGQK	C_2_HC	CX_7_CX_23_HXC	1	III	VrWRKY80	Vradi0214s00140.1	WRKYGQK	C_2_HC	CX_7_CX_23_HXC	1	III
VaWRKY74	Vang03g09520.1	—	C_2_HC	CX_7_CX_23_HXC	1	III	VrWRKY81	Vradi04g05450.1	WRKYGQK	C_2_HC	**CX**_**6**_ **CX**_**23**_ **HXC**	1	III
VaWRKY75	Vang07g06810.1	WRKYGQK	C_2_HC	CX_7_CX_23_HXC	1	III	VrWRKY82	Vradi09g05200.1	WRKYGQK	C_2_HC	CX_7_CX_23_HXC	1	III
VaWRKY76	Vang10g06010.1	WRKYGQK	C_2_HC	CX_7_CX_23_HXC	1	III	VrWRKY83	Vradi0338s00040.1	WRKYGQK	C_2_HC	CX_7_CX_23_HXC	1	III
VaWRKY77	Vang0340s00050.1	WRKYGQK	C_2_HC	CX_7_CX_23_HXC	1	III	VrWRKY84	Vradi0338s00060.1	—	C_2_HC	CX_7_CX_23_HXC	1	III
VaWRKY78	Vang0228s00170.1	WRKYGQK	C_2_HC	CX_7_CX_23_HXC	1	III	VrWRKY85	Vradi05g05170.1	WRKYGQK	C_2_HC	CX_7_CX_23_HXC	1	III
VaWRKY79	Vang0459s00030.1	WRKYGQK	C_2_HC	CX_7_CX_23_HXC	1	III							
VaWRKY80	Vang0459s00020.1	WRKYGQK	C_2_HC	CX_7_CX_23_HXC	1	III							
VaWRKY81	Vang0352s00010.1	—	C_2_HC	CX_7_CX_23_HXC	1	III							
VaWRKY82	Vang0459s00010.1	WRKYGQK	C_2_HC	CX_7_CX_23_HXC	1	III							
VaWRKY83	Vang04g07160.1	—	C_2_HC	CX_7_CX_23_HXC	1	III							
VaWRKY84	Vang1880s00010.1	—	C_2_HC	CX_7_CX_23_HXC	1	III							

Note: Any variation in the heptapeptide WRKYGQK sequence or zinc finger motif is indicated in ‘Bold’.

### Gene exon-intron structure organization

**Figure 2:**
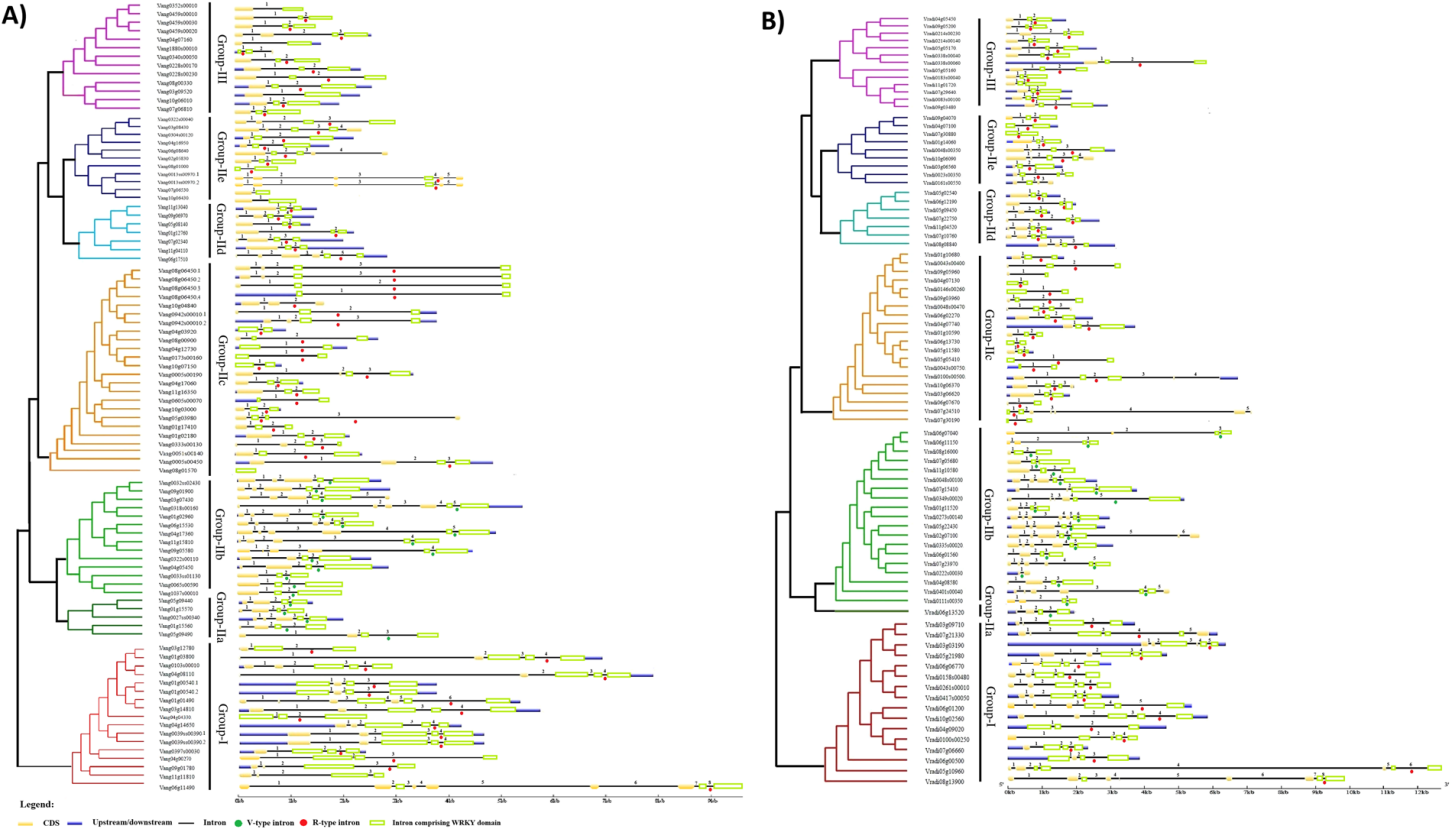
(**A**) Phylogenetic relationship and exon-intron arrangement of *VaWRKY* genes (including isoforms; the digit after the decimal in Gene ID indicates the isoforms). Multiple alignment of *VaWRKY* gene sequences was executed by Clustal W and the phylogenetic tree was created using MEGA 6.0 by the Neighbor-Joining (NJ) method with 1,000 bootstrap replicates. The exon-intron arrangement was executed using Gene Structure Display Server 2.0. The exons and introns were represented by yellow boxes and black lines. The size of introns can be estimated using the scale given at the bottom. The V-type introns in Group IIa and Group IIb genes and R-type introns present in the remaining genes were indicated as green and red closed circles below the introns, respectively. (**B**) Phylogenetic relationship and exon-intron arrangement of *VrWRKY* genes. Multiple alignment of *VrWRKY* gene sequences was executed by Clustal W and the phylogenetic tree was created using MEGA 6.0 by the Neighbor-Joining (NJ) method with 1,000 bootstrap replicates. The exon-intron arrangement was executed using Gene Structure Display Server 2.0. The exons and introns were represented by yellow boxes and black lines. The size of introns can be estimated using the scale given at the bottom. The V-type introns in Group IIa and Group IIb genes and R-type introns present in the remaining genes were indicated as green and red closed circles below the introns, respectively.

The gene structural similarity and diversity plays salient role in the evolution of gene family. To study this, we generated exon-intron map of the *VaWRKY* and *VrWRKY* genes, using the software Gene Structure Display Server. The detailed representation of the coding region, introns and upstream or downstream regions of genes, was provided in Fig. 2. Introns, which are integral elements of eukaryotic genomes, actively participate in the genomic recombination leading to gene rearrangements and evolution. The group I genes have 2 to 8 introns. Most of the Group IIa genes and Group III possess 3 and 2 introns, respectively. Variable number of introns were found in Group IIb genes, 2 to 5 introns in *VaWRKY* genes, and 2 to 6 introns in *VrWRKY* genes. In Group IIc *VaWRKY* members, 0 to 3 introns were found with *VaWRKY36 (Vang08g01570*) possessing no intron, exceptionally. Whereas, 1 to 5 introns were found in case of Group IIc *VrWRKY* genes. In Group IId, the *VaWRKY* genes possess 2 to 5 number of introns, but the *VrWRKY* genes have 2 to 3 introns, showing less variation. Also, in Group IIe members, 1 to 5 and 2 to 4 introns observed in *VaWRKY* and *VrWRKY*, respectively. This variable distribution of introns in Group IIb, IIc and IIe members including Group IId members of Adzuki bean, indicate that both exon loss and gain has occurred during their evolution, which may explain why closely related *WRKY* genes falling in the same group can be diverse in function^31–35^.

Although, most of the *WRKY* genes were rich in introns, but the region encoding N-terminal WRKY domain possess only one intron, with the exception case of that region encoding N-terminal domain in Group I members lack introns (Supplementary Fig. S2). Two types of introns, namely R (Arg)-type and V (Val)-type were found in most of the *VaWRKY* and *VrWRKY* genes (indicated in Fig. 2), discussed later in Section 3.4.

Furthermore, in Adzuki bean, by studying the exon and intron structure of the isoforms, we found that the pairs Vang0942s00010.1 and Vang0942s00010.2 (VaWRKY51) have 2 and 3 introns respectively. The gene structure of the pairs Vang0013ss00970.1 and Vang0013ss00970.2 (VaWRKY62) having a difference of only 9 amino acids, was highly similar with 5 introns. The variants Vang08g06450.1, Vang08g06450.2, Vang08g06450.3 and Vang08g06450.4 (VaWRKY52) have 3, 3, 2 and 1 introns, respectively. Also, their exon-length varies (Fig. 2). The study also revealed that two pairs of VaWRKY TFs, Vang0039ss00390.1 and Vang0039ss00390.2 (VaWRKY12*)*; and Vang01g00540.1 and Vang01g00540.2 (VaWRKY15) may be redundant proteins, as they have no difference in the protein sequence.

### Chromosome location and gene duplication

**Figure 3:**
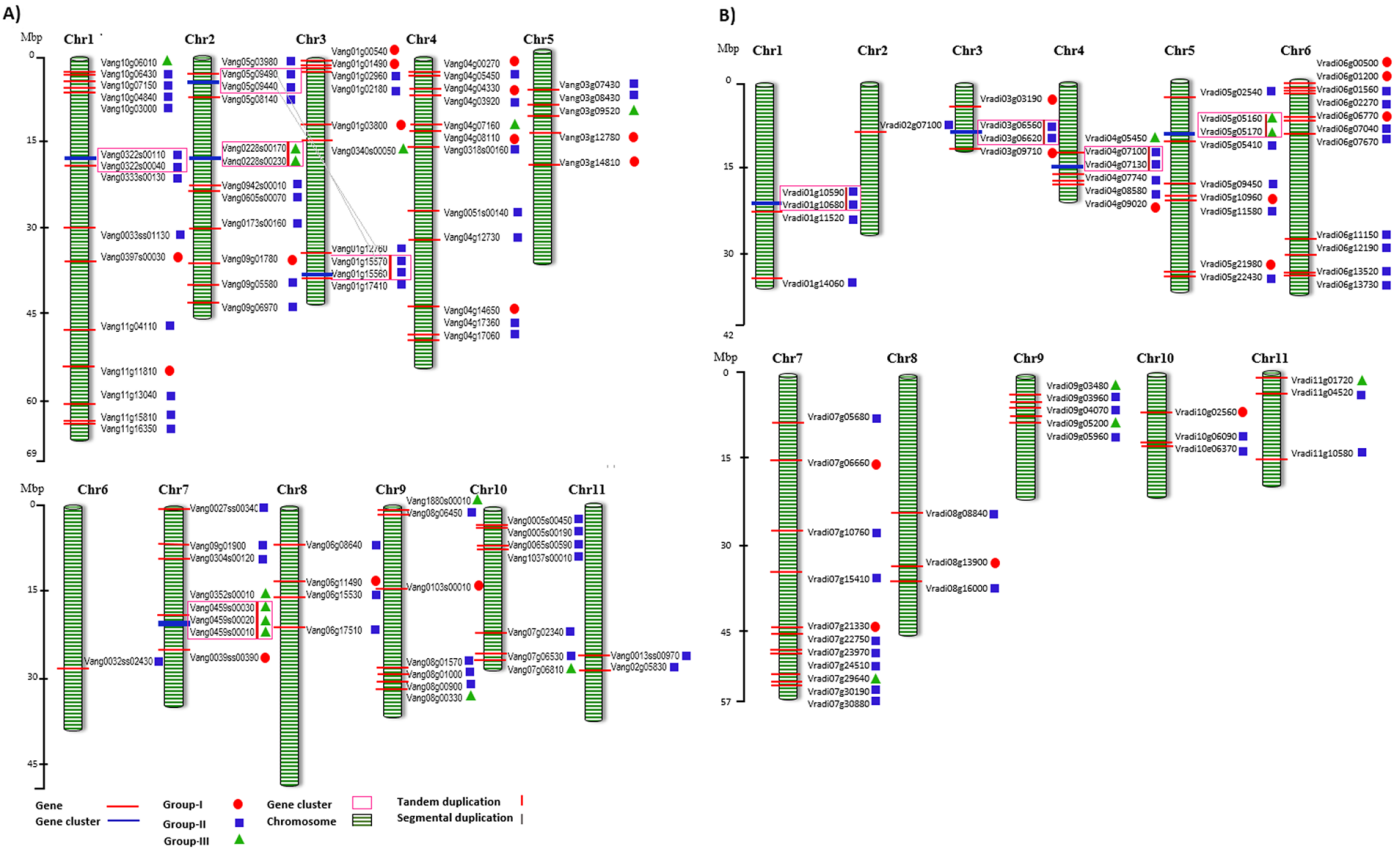
(**A**) Physical mapping of *VaWRKY* genes on chromosome. Distribution of *VaWRKY* genes on the corresponding eleven chromosomes which are represented as green bars, were shown as red lines. The approximate position of these genes can be estimated from the scale given at the left side. The exact position of the genes was mentioned in Supplementary Table S3. The markers next to the gene names indicate the group to which each *WRKY* gene belongs to. The five gene clusters found in chromosome 1, 2, 3 and 7 are depicted as blue horizontal lines with gene names encircled in pink open rectangles. Tandemly duplicated genes were indicated as red vertical lines. The segmental duplications were shown as grey linker lines. (**B**) Physical mapping of *VrWRKY* genes on chromosome. Distribution of *VrWRKY* genes on the corresponding eleven chromosomes which are represented as green bars, were shown as red lines. The approximate position of these genes can be estimated from the scale given at the left side. The exact position of the genes was mentioned in Supplementary Table S3. The markers next to the gene names indicate the group to which each *WRKY* gene belong to. The five gene clusters found in chromosome 1, 3, 4 and 5 are depicted as blue horizontal lines with gene names encircled in pink open rectangles. Tandemly duplicated genes were indicated as red vertical lines.

Out of 84, only 83 of *VaWRKY* gene*s* could be mapped on the chromosome. The precise location of one gene *Vang04g16950* (*VaWRKY71*) could not be determined. As represented in Fig. 3A, most of the genes (about 60%) are located in chromosome 1 to 4, followed by chromosome 7, 9, 10 and 5. Only a few genes are located on chromosome 6, 8 and 11. Fifteen genes (two Group I, twelve Group II and one Group III) were mapped on chromosome 1; twelve genes (one Group I, nine Group II and two Group III) were mapped on chromosome 2; ten genes (three Group I, six Group II and one Group III) were mapped on Chromosome 3; twelve genes (four Group I, seven Group II and one Group III) were mapped on chromosome 4; five genes (two Group I, two Group II and one Group III) mapped on chromosome 5; one gene (Group II) was mapped on chromosome 6; eight genes (one Group I, three Group II and four Group III) were mapped on chromosome 7; four genes (one Group I and three Group II) were mapped on chromosome 8; seven genes (one Group I, four Group II and two Group III) were mapped on chromosome 9; seven genes (six Group II and one Group III) were mapped on chromosome 10; and two genes (Group II) were mapped on chromosome 11.

The locus of 25 *VrWRKY* genes could be documented only on scaffolds, but not on chromosome, due to lack of information. As represented in Fig. 3B, most of the *VrWRKY* genes are located on chromosome 4 to 7. Four genes (Group II) were mapped on chromosome 1; one gene (Group II) was mapped on chromosome 1; four genes (two Group I and two Group II) were mapped on chromosome 3; six genes (one Group I, four Group II and one Group III) were mapped on chromosome 4; nine genes (two Group I, five Group II and two Group III) were mapped on chromosome 5; eleven genes (three Group I and eight Group II) were mapped on chromosome 6; eleven genes (two Group I, eight Group II and one Group III) were mapped on chromosome 7; three genes (one Group I and two Group II) were mapped on chromosome 8; five genes (three Group II and two Group III) were mapped on chromosome 9; three genes (one Group I and two Group II) were mapped on chromosome 10; and three genes (two Group II and one Group III) were mapped on chromosome 11. The detailed description of chromosome location and gene-length is mentioned in Supplementary Table S3.

It is well established that multiple members of WRKY gene family that form a large and extensively complex regulative network to control complicated physiological processes were expanded as a result of the long evolutionary history^40^. The individual genes undergo a series of genomic recombination and amplification during the process of evolution. The major player in this event are recent gene duplications, resulting in many paralogous pairs in different species^41,42^. Tandem duplication is one way of gene duplication, where a set of two or more genes located in the same chromosome within the range of 100-kb distance, separated by zero or few spacer genes^43^. The alternate type of gene duplication is large-scale gene-duplication (whole genome or segmental duplication) where a block of genes is duplicated in a different chromosome. Furthermore, a gene cluster is defined as a chromosome region with two or more genes located within 200 kb sequence.

To unravel the mechanism of *VaWRKY* gene evolution, we explored gene duplication events. We found that, eleven *VaWRKY* genes formed five gene clusters. Chromosome 1, 3 and 7 contains 1 gene cluster. Whereas, two gene clusters were located on chromosome 2. Out of these five gene cluster, three were tandem duplications; the gene pairs *Vang0228s00170* (*VaWRKY78*) and *Vang0228s00230* (*VaWRKY72*) located on chromosome 2 with three spacer genes, *Vang01g15570* (*VaWRKY17*) and *Vang01g15560* (*VaWRKY19*) located on chromosome 3 with zero spacer gene, and *Vang0459s00010* (*VaWRKY82*), Vang0459s00030 (*VaWRKY79*) and *Vang0459s00020* (*VaWRKY80*) located on chromosome 7 with one and zero spacer genes (Fig. 3A). Further analysis of intron lengths and gene structure of the tandem duplicated gene revealed that, recombination might have taken place in addition to duplication event, which explains the variation in the encoded proteins. The gene pairs *Vang05g09440* (*VaWRKY18)* and *Vang05g09490* (*VaWRKY20*) located on chromosome 2 were also observed to be duplicated on chromosome 3 as gene pair *Vang01g15570* (*VaWRKY17*) and *Vang01g15560* (*VaWRKY19*). Similarly, 12 *VrWRKY* genes formed six gene clusters, with one gene cluster located on chromosome 1, 3, 4 and 5 each and two gene clusters located on scaffolds. Four of them were tandem duplications; *Vradi04g07100* (*VrWRKY65*) and *Vradi04g07130* (*VrWRKY50*) located on chromosome 4 with two spacer genes; *Vradi05g05160* (*VrWRKY78)* and *Vradi05g05170* (*VrWRKY85*) located on chromosome 5 with zero spacer genes; *Vradi0214s00140* (*VrWRKY80)* and *Vradi0214s00230* (*VrWRKY79*), and *Vradi0338s00040* (*VrWRKY83*) and *Vradi0338s00060* (*VrWRKY84*) located on scaffolds with zero spacer gene. In case of both Adzuki bean and Mung bean, the tandem and segmental gene duplication events are not that significant, suggesting that these phenomena do not play much significant role in the evolution of *VaWRKY* and *VrWRKY* genes.

### Multiple sequence alignment and phylogenetic tree analysis

The analysis of multiple sequence alignment of VaWRKY and VrWRKY domains revealed that mutations occurred at W, R and Q amino acids in the conserved WRKYGQK heptapeptide (indicated in Table 1). The detailed study showed that the variation arose from amino acid substitutions of R to K to give WKKYGQK, or from Q to E or K to give WRKYGEK or WRKYGKK. In case of Adzuki bean, WRKYGQK residue was substituted by SRKYGQK due to mutation at W position. The WRKYGKK substitutions were most predominant. Interestingly, the 5 VaWRKY proteins and 5 VrWRKY proteins carrying these mutations occur in the same subgroups IIc. The zinc-finger domains in different groups and subgroups also have atypical nature. The N-terminal and C-terminal WRKY domain of Group I proteins have CX_4_CX_22_HXH and CX_4_CX_23_HXH type zinc-finger motif, respectively. The Group II proteins have CX_5_CX_23_HXH type zinc- finger motif, except the Group IIc members which have CX_4_CX_23_HXH type zinc-finger motif. The Group III members have their atypical CX_7_CX_23_HXC type zinc-finger motifs.

**Figure 4:**
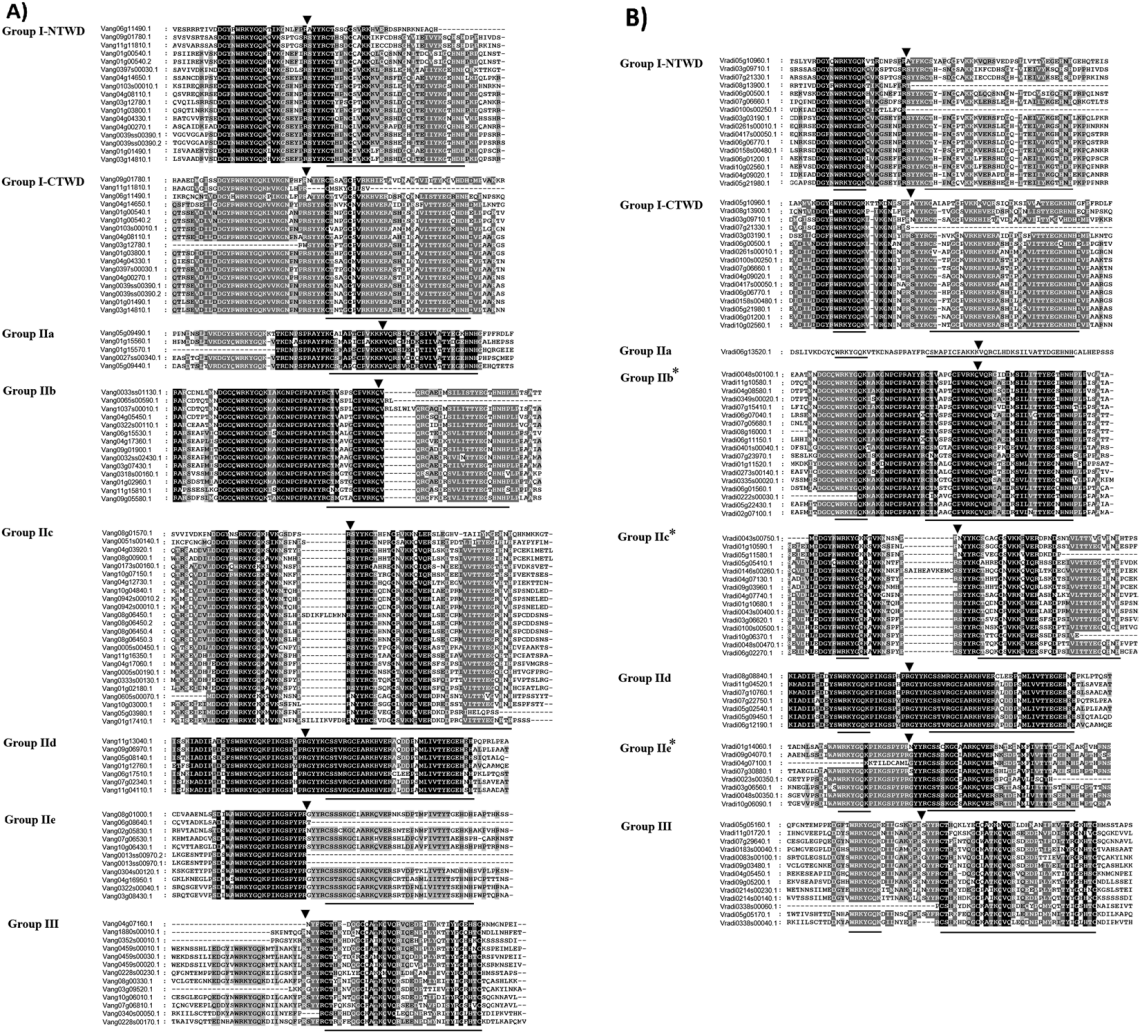
(**A**) Multiple sequence alignment of VaWRKY domains. Alignment was performed using Clustal Omega program and is displayed using GenDoc tool. The amino acid residues which were highly conserved within the major groups/subgroups are indicated in black. The WRKYGQK heptapeptide and the zinc-finger domain were underlined. The position of the conserved introns R-type and V-type intron was indicated by an arrow head. NTWD and CTWD stand for N-terminal WRKY domain and C-terminal WRKY domain, respectively. (**B**) Multiple sequence alignment of VrWRKY domains. Alignment was performed using Clustal Omega program and is displayed using GenDoc tool. The amino acid residues which were highly conserved within the major groups/subgroups are indicated in black. The WRKYGQK heptapeptide and the zinc-finger domain were underlined. The position of the conserved introns R-type and V-type intron was indicated by an arrow head. NTWD and CTWD stand for N-terminal WRKY domain and C-terminal WRKY domain, respectively. (*) Few truncated domains were excluded from the study for better representation.

The two types of introns in WRKY domains are indicated in Fig. 4. The R-type introns, which were phase-2 introns spliced exactly after the R position, before the zinc-finger motif, similar to the splicing position observed in *Arabidopsis*. However, the V-type introns were phase-0 introns, located just before the V position which is the sixth amino acid after the second cysteine residue in the C_2_H_2_ zinc finger motif.

**Figure 5:**
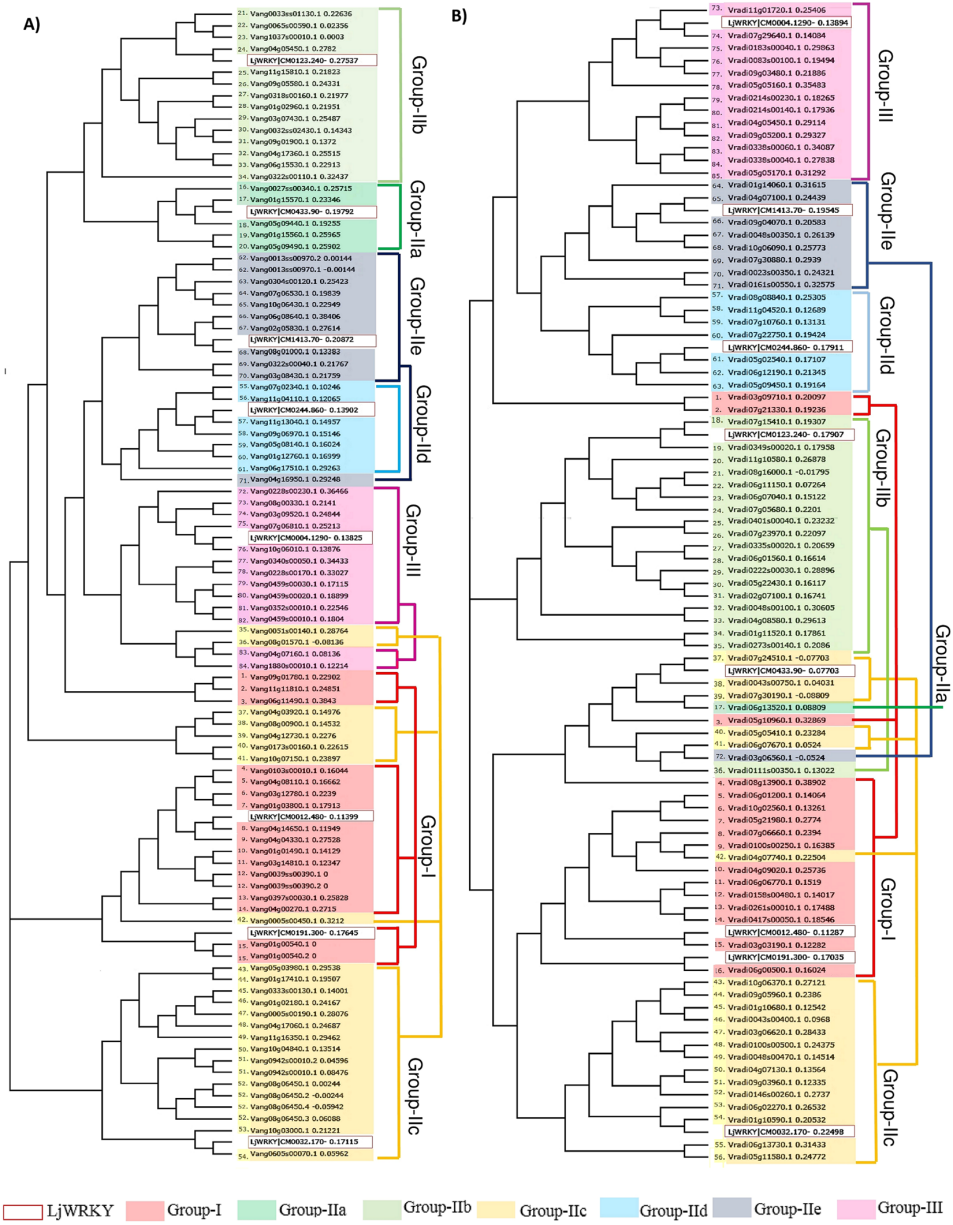
Phylogenetic tree of (**A**) VaWRKY and (**B**) VrWRKY protein family. The phylogenetic tree was created using Clustal Omega with default settings. *Lotus japonica* WRKY proteins from each group/subgroup were used as reference.

Based on the phylogenetic analysis of conserved WRKY domains, the VaWRKY and VrWRKY members were subdivided into 8 clades (Supplementary Fig. S2). In case of Group I members, the two WRKY domains, designated as Group I- NTWD and Group I-CTWD, clustered into two separate clades due to the divergence in their sequences. The clade of Group IIa was close to that of Group IIb, whereas the Group IId and IIe member were clustered, representing their origination from common gene ancestor or evolution under similar selection pressure. The Group III members are more similar to the Group I-NTWD members as compared to any other subgroup, suggesting that may have shared a common ancestor before their divergence into Group I and Group III, or the Group III members have been originated from Group I genes due to mutation in their zinc-finger domain after losing the C-terminal WRKY domain. Similarly, Group IIc members are more closely related to the Group I-CTWD members. This suggests that the Group IIc WRKY proteins might have been originated from the Group I proteins after the loss of the N- terminal domain. Moreover, two Group IIc members, Vang08g01570.1 (VaWRKY36) and Vang0051s00140.1 (VaWRKY35), and one member namely Vang0005s00450.1 (VaWRKY42) have been clustered with Group I-NTWD and Group I-CTWD members respectively, suggesting a common origin of their domains. Also in case of Mung bean, one Group IIc member namely Vradi04g07740.1 (VrWRKY42) clustered with Group I-CTWD member. Both of the two WRKY domains of Group I member Vradi05g10960.1 (VrWRKY3) clustered with the Group IIa member Vradi06g13520.1 (VrWRKY17), suggesting that VrWRKY3 has been originated due the duplication of VrWKY17 domain Supplementary Fig. S2. The phylogenetic relationships of the whole WRKY proteins sequence are illustrated in Fig. 5, where some of the Group IIc members have been clustered in Group I and Group III. This may be due to more sequence similarity in the region outside the domain of respective members.

### Motif analysis

**Figure 6:**
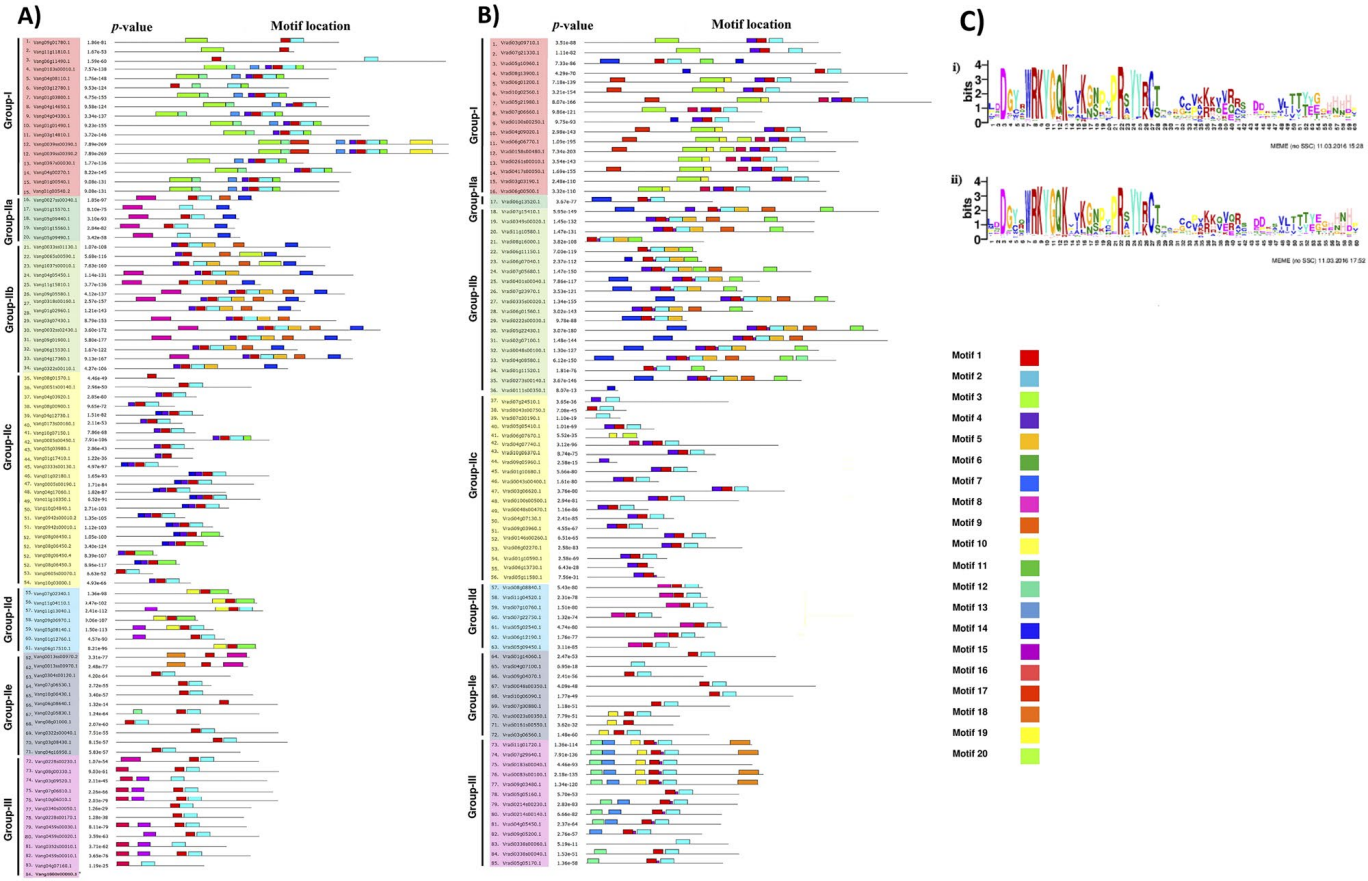
(**A**) Motif analysis of VaWRKY protein family. The distribution of 20 conserved motifs in VaWRKY proteins, identified by MEME program, was shown as colored boxes. The sequences of these conserved motifs were listed in Supplementary Table S4. *No conserved motif could be found in VaWRKY84 protein. (**B**) Motif analysis of VrWRKY protein family. The distribution of 20 conserved motifs in VrWRKY proteins, identified by MEME program, was shown as colored boxes. The sequences of these conserved motifs were listed in Supplementary Table S4. (**C**) Analysis of the consensus sequence of the WRKY domain in (i) VaWRKY family, and (ii) VrWRKY family. Analysis of the 91 VaWRKY proteins and 85 VrWRKY proteins was performed using the MEME suite. The overall height in each stack indicates the sequence conservation at each position. The height of each residue letter is proportional to the relative frequency of the corresponding residue. Amino acids are colored according to their chemical properties: green for polar, non-charged, non-aliphatic residues (N,Q,S,T), magenta for the most acidic residues (D,E), blue for the most hydrophobic residues (A,C,F,I,L,V,W and M), red for positively charged residues (K,R), pink for histidine (H), orange for glycine (G), yellow for proline (P) and turquoise for tyrosine (Y).

Apart from the conserved residue of 60 amino acids, other motifs also lie in the rest of the protein sequence, which may perform unknown functional or structural roles. To investigate further the similarity and diversity of motif, MEME analysis was performed to investigate conserved stretches of amino acids ranging from 6 to 50 amino acids^44^. The distribution of the 20 conserved motifs discovered significantly varied among different WRKY proteins (Fig. 6). In case of VaWRKY, five motifs, motif 1, motif 2, motif 3, motif 4 and motif 6, together comprise the WRKY domain, out of which motif 1 and motif 3 comprise the conserved heptapeptide (Supplementary Table S4). Whereas, six motifs, motif 1, motif 2, motif 3, motif 10, motif 15 and motif 16 makes the VrWRKY domains, with the WRKYGQK heptapeptide located in motif1 and motif 3. Out of the 15 remaining non-redundant motifs in VaWRKY and 14 non-redundant motifs in VrWRKY, the function of the majority of motifs could not be predicted. We found only three functional motifs of plant-zinc cluster domain, bZIP motif and NLS, identified by MEME in both VaWRKY and VrWRKY proteins (Supplementary Fig. S5). The plant-zinc cluster domains of length 29 amino acids, indicated as motif 10 (VaWRKY) and motif 8 (VrWRKY), were explicitly found in Group IId proteins (except in VaWRKY60). The 41–44 amino acid long bZIP like motif denoted as motif 8 in case of VaWRKY, were distributed predominantly in Group IIa and Group IIb, but also in two Group IIe proteins (variants of VaWRKY62) and one Group III protein (VaWRKY72), exceptionally. In case of VrWRKY proteins, bZIP motifs indicated as motif 7 occur predominantly in Groups IIa and Group IIb proteins, with one exception of Group I protein (VrWRKY3). The function of these domains in WRKY proteins is still unclear. Nuclear localization sequences were also identified by MEME analysis, in various Group IIc VaWRKY members, whereas in Group IIe and Group III members, in case of VrWRKY proteins. Motif5 and motif7 are specific and commonly shared by closely related Group IIa and Group IIb members of VaWRKY. Similarly, motif5 and motif6 are unique to Group IIb members in Mung bean. Interestingly, these motifs were similar to what were found in Group IIa and Group IIb members of *L*. *japonica*^45^. Group III WRKY proteins also possess some conserved motifs unique to their members. For instance, motif 15 and motif 16 in VaWRKY proteins and, motif 12 and motif 13 in VrWRKY proteins, are found mainly in Group III members. Moreover, like *Arabidopsis* and Rice, a HARF motif (RTGHARFRR (A/G) P) was also found in five VaWRKYs and three VrWRKYs of Group IId, manually^39^.

### Expression of *WRKY* genes in Adzuki bean and Mung bean

**Figure 7:**
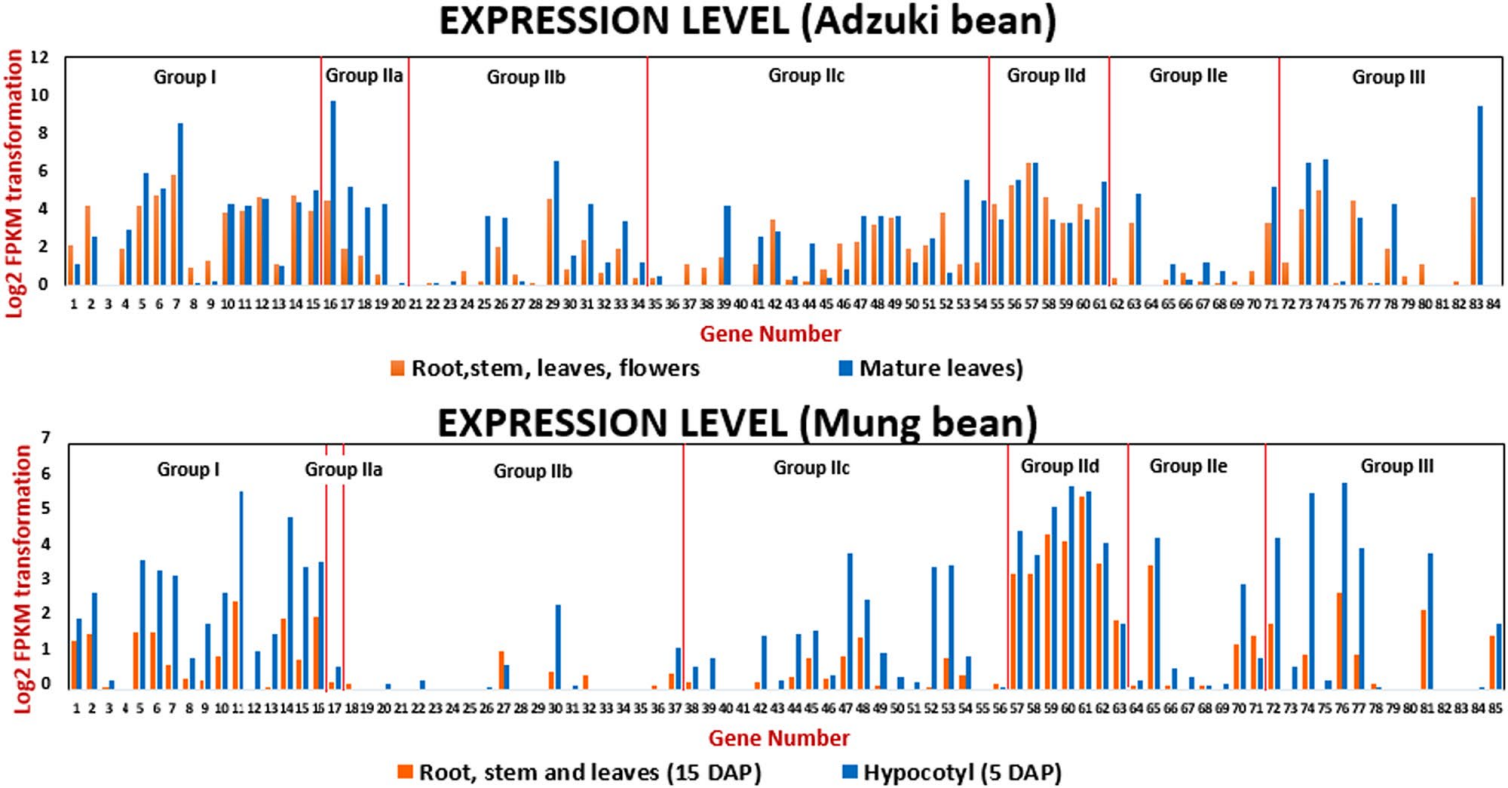
RNA sequence analysis of *VaWRKY* and *VrWRKY* genes. The RNA sequence reads of Adzuki bean and Mung bean were aligned on their genome to obtain the fragments per kilobase of exon per million fragments mapped (FPKM) values. The log2 transformation of the FPKM values given in Supplementary Table S6, were represented as gene expression in the histogram plot.

To explore the expression of *WRKY* genes in Adzuki bean and Mung bean, we analyzed and calculated the RNA sequence data available for some tissues^46–49^. The FPKM value of 0.5 was considered as threshold for the detection of significant expression. 57 of the 84 predicted *VaWRKY* genes and 47 of the 85 predicted *VrWRKY* genes were found to be expressed significantly at least in one of the major tissues of Adzuki bean and Mung bean included in the study. The genes with zero FPKM value are not considered in this study. Perhaps these genes expressed in other tissues, or during a different developmental stage not included in this RNA-seq experiment. This study revealed that *WRKY* genes belonging to same groups/subgroups have differential expression. For instance, most of the Group IId members showed significant expression with high FPKM values. Whereas, the Group IIe and Group IIb expressed very low, in Adzuki bean and Mung bean, respectively. In Group I, some *VaWRKY* genes have low FPKM values like 0.85 (*VaWRKY8*) while some genes have high FPKM value as 56.68 (*VaWRKY7*) (Supplementary Table S6). Such variation in the expression level of *VaWRKY* and *VrWRKY* genes within other subgroups/groups can be easily visualized in the histogram (Fig. 7), suggesting the *WRKY* genes possessing similar domains express differently depending on the functional diversity. We also investigated the expression of closely located *VaWRKY* genes forming gene clusters. Their differential expression suggests their involvement in non-redundant signaling pathways.

### Prediction of putative stress-responsive WRKY in Adzuki bean and Mung bean and their promoter analysis

**Figure 8:**
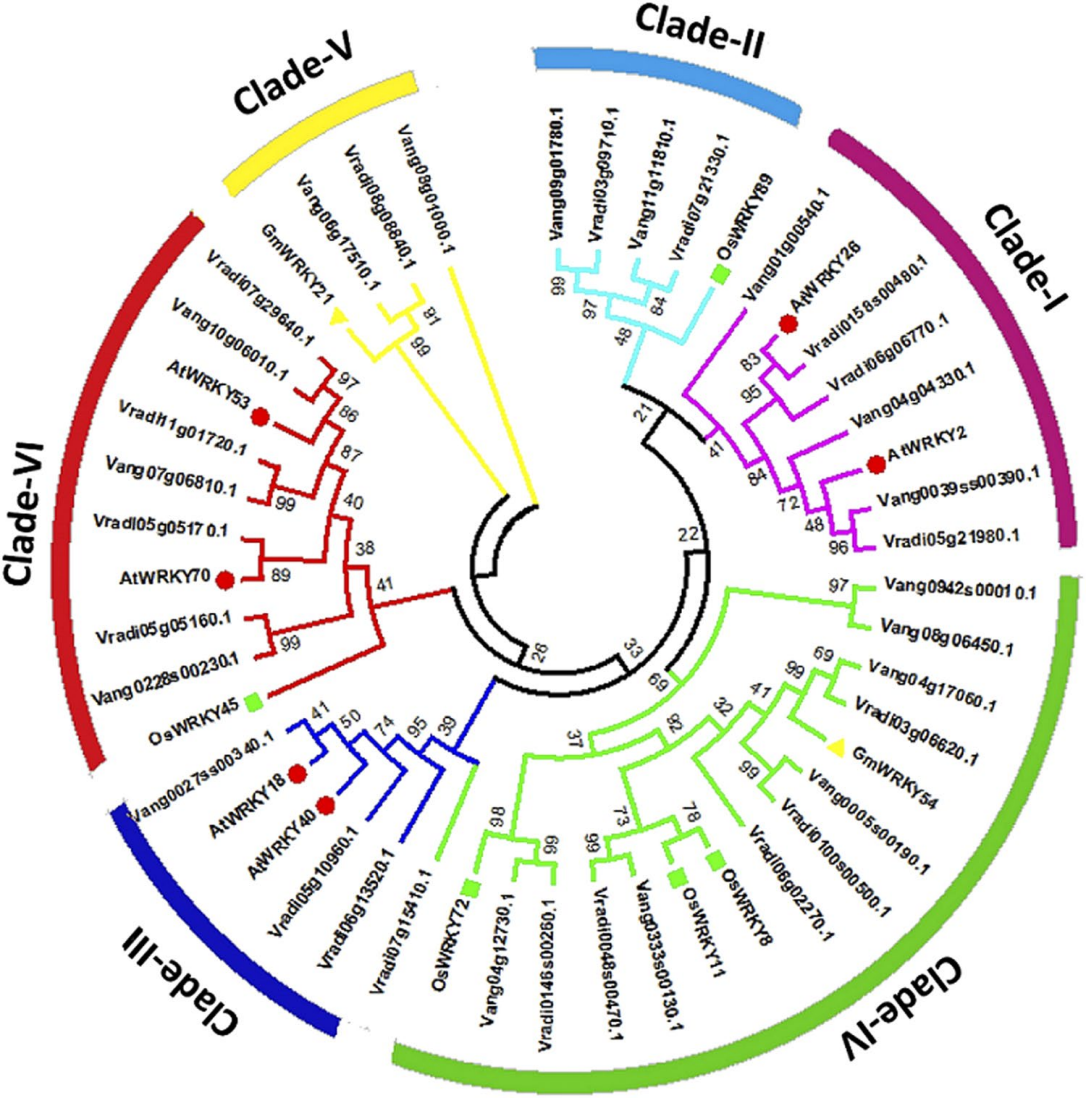
Putative stress-responsive VaWRKY and VrWRKY proteins. The tree was created with MEGA 6.0 tool by the Neighbor-Joining (NJ) method with 1,000 bootstrap replicates. The AtWRKY, OsWRKY and GmWRKY proteins reported to be involved in various stress-responses, used as reference, were indicated by red closed circle, green closed square and yellow closed triangle, respectively. Their respective homologs in VaWRKY and VrWRKY family clustered with them forming distinct clades (indicated in different colors).

The interaction between transcription factor and the stress-inducible *cis*-acting regulatory elements present in the promoter modulates the expression of gene regulatory networks response to the respective physical, environmental and biological stress. As these elements are highly conserved among orthologous or paralogous and co-regulatory genes, we investigated stress-responsive homologs of AtWRKY, OsWRKY and GmWRKY proteins reported to be involved in various abiotic and biotic stresses. Subsequently, the −1.5 kb promoter region of those putative stress-responsive candidates were analyzed using two different promoter analysis tool, PlantCare and PLACE, to identify stress-responsive *cis*-elements. Based on the phylogenetic data (Supplementary Fig. S7), the VaWRKY and VrWRKY members which clustered closest to the reported stress-responsive AtWRKY, OsWRKY and GmWRKY proteins, used as references, were selected as putative homologous stress-responsive candidates (Supplementary Fig. S7 and Table S8). A total of 17 VaWRKY and 18 VrWRKY proteins were chosen, which were also represented as phylogenetic tree along with their respective homolog members in *Arabidopsis*, Rice or Soybean, forming six different clades, depending on their respective groups and subgroups (Table 2 and Fig. 8). The stress-responsive elements recognized in the promoter of these genes are listed Supplementary Table S9. Exceptionally, Vradi07g15410 (VrWRKY18), a putative homolog of OsWRKY72 (Supplementary Fig. S7), clustered in Clade III rather than Clade IV which accommodates rest of the OsWRKY72 homologs. Furthermore, the promoter analysis also revealed intermediate functional similarity with Clade III and Clade IV members.

**Table 2:** Putative stress-responsive VaWRKY and VrWRKY.

VaWRKY		Group	Putative inducer stress	VrWRKY		Group	Putative inducer stress
**1**	Vang0039ss00390	**I**	Heat, drought, senescence and osmotic stress	**1**	Vradi05g21980	**I**	Heat, cold, drought, senescence and osmotic stress
**2**	Vang04g04330	**I**	Heat, cold and senescence	**2**	Vradi0158s00480	**I**	Heat and drought
**3**	Vang04g05450	**I**	Cold, senescence, biotic stress	**3**	Vradi06g06770	**I**	Senescence
**4**	Vang09g01780	**I**	Cold, drought, senescence and biotic stress	**4**	Vradi03g09710	**I**	ABA, heat, cold, drought, senescence and biotic stress
**5**	Vang11g11810	**I**	ABA, drought and biotic stress	**5**	Vradi07g21330	**I**	ABA, cold, drought and biotic stress
**6**	Vang0027ss00340	**IIa**	ABA, heat, cold, drought, salinity, osmotic stress and salicylic acid	**6**	Vradi05g10960	**I**	Drought, senescence, salicylic acid biotic stress
				**7**	Vradi06g13520	**IIa**	ABA, drought, cold, osmotic stress and biotic stress
**7**	Vang0942s00010	**IIc**	ABA, drought, heat, cold and biotic stress	**8**	Vradi07g15410	**IIb**	ABA, drought, osmotic stress and biotic stress
**8**	Vang08g06450	**IIc**	ABA, drought, osmotic stress and biotic stress	**9**	Vradi0146s00260	**IIc**	ABA, cold, senescence and salicylic acid
**9**	Vang0333s00130	**IIc**	ABA, salinity, cold, osmotic stress, biotic stress	**10**	Vradi06g02270	**IIc**	ABA, cold, drought, senescence, osmotic stress, heat and biotic stress
**10**	Vang0005s00190	**IIc**	ABA, drought and senescence	**11**	Vradi0048s00470	**IIc**	ABA, cold, drought, osmotic stress and biotic stress
**11**	Vang04g12730	**IIc**	ABA, cold and senescence	**12**	Vradi0100s00500	**IIc**	ABA, drought and senescence
**12**	Vang04g17060	**IIc**	ABA, drought and osmotic stress	**13**	Vradi03g06620	**IIc**	ABA, drought and salinity
**13**	Vang06g17510	**IId**	ABA, heat, drought, osmotic stress and biotic stress	**14**	Vradi08g08840	**IId**	ABA and drought
**14**	Vang08g01000	**IIe**	Drought, senescence and biotic stress				
**15**	Vang10g06010	**III**	Drought, osmotic stress, cold and biotic stress	**15**	Vradi07g29640	**III**	Drought, osmotic stress and biotic stress
**16**	Vang07g06810	**III**	Drought, heat, cold, biotic stress	**16**	Vradi11g01720	**III**	Osmotic stress and biotic stress
**17**	Vang0228s00230	**III**	ABA, senescence, heat, cold and drought	**17**	Vradi05g05170	**III**	ABA, osmotic stress and biotic stress
				**18**	Vradi05g05160	**III**	Heat, cold and senescence

**Figure 9:**
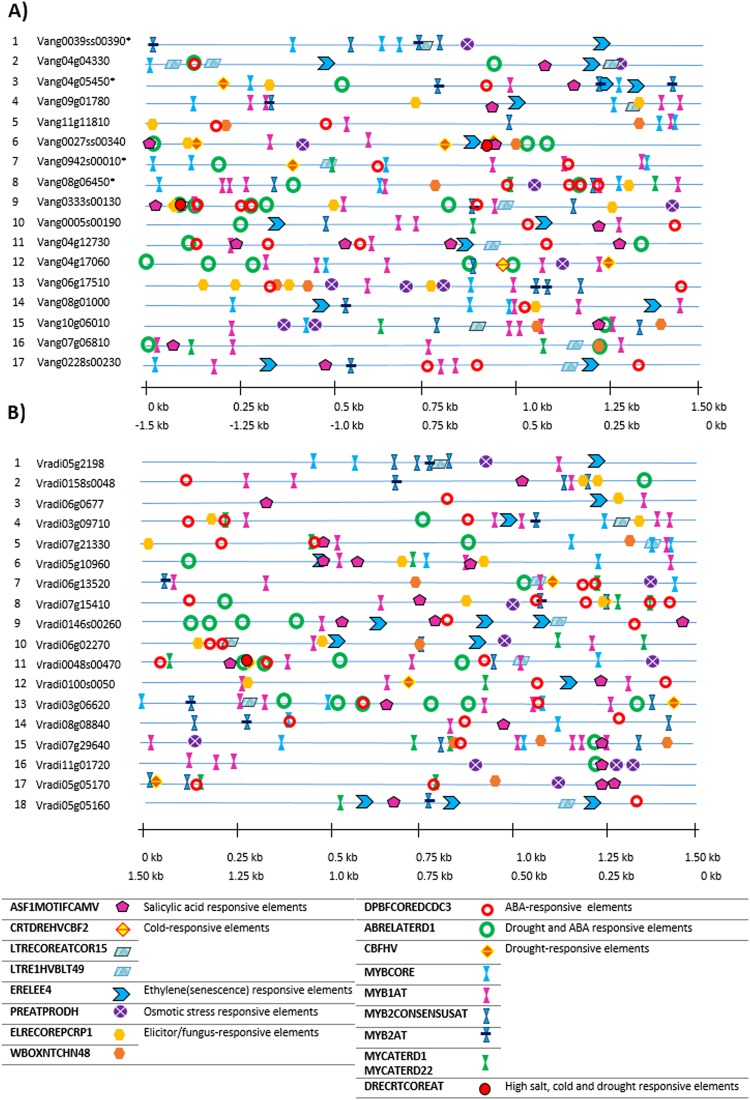
Cis- regulatory stress-responsive elements identified in the 1.5 kb upstream promoter region of (**A**) Stress-responsive *VaWRKY* candidates (**B**) Stress-responsive *VrWRKY* candidates. The elements commonly identified by both PlantCare and PLACE, and those involved in major stresses were chosen for pictorial representation. The sequence and position of these elements and some additional elements, not mentioned here, are described in Supplementary Tables S10 and S11. The first scale represents the location of *cis*-elements mentioned in the promoter analysis data. The second scale denotes the position of those elements from the ‘start codon’ as zero reference point. The candidates possessing isoforms are indicated by an asterisk (*).

We found *cis*-elements involved in various stresses, for instance, ‘DPBFCOREDCDC3’ induced by abscisic acid, ‘ABRELATERD1’ involved in abscisic acid signaling and drought stress, ‘ERELEE4’ responsive to ethylene, ‘PREATPRODH’ involved in osmotic stress, ‘DRECRTCOREAT’ induced by high salt, cold and drought etc. Interestingly, in both the Adzuki bean and Mung bean, we found stress-inducible elements similar to their respective homologs in *Arabidopsis* and Rice (Supplementary Table S8). The location of these elements are depicted in Fig. 9. The detailed interpretation of these stress-responsive elements and their functions are mentioned in the Supplementary Tables S10 and S11.

The clade I members (as indicated in Fig. 8), predominantly consist of *cis*-elements responsive to osmotic stress, drought, and senescence. The clade II members possess elements mainly induced by ABA, drought, and pathogens. Few members also possess cold and senescence responsive elements. The clade III members possess elements generally induced by ABA, salt, salicylic acid including pathogens. The clade IV and V members predominantly possess elements responsive to drought, ABA and biotic stress, which were also found in stress-responsive OsWRKY members. Whereas clade VI containing the Group III members chiefly contained elements involved in the heat, cold, drought, osmotic stress, senescence and biotic stress, similar to as found in *AtWRKY* and *OsWRKY*.

The abundance of ABA-responsive element ‘DPBFCOREDCDC3’ in Vang04g12730 (VaWRKY39), Vang0333s00130 (VaWRKY45), Vang08g06450 (VaWRKY52), Vradi07g15410 (VrWRKY18), and Vradi0048s00470 (VrWRKY49), and the element ‘ABRELATERD1’ in Vang0333s00130 (VaWRKY45), Vang04g17060 (VaWRKY48), Vradi03g06620 (VrWRKY47), Vradi0048s00470 (VrWRKY48), and Vradi06g02270 (VrWRKY53), suggests their significant role in ABA signaling. The osmotic stress-element ‘PREATPRODH’ is more profound in Vang06g17510 (VaWRKY61) and Vradi11g01720 (VrWRKY73). The elements ‘DRECRTCOREAT’ reported to be involved in high salt, drought, and cold is found in three gene promoters, Vang0027ss00340 (VaWRKY16), Vang0333s00130 (VaWRKY45) and Vradi0048s00470 (VrWRKY49). Similarly, Vang11g11810 (VaWRKY2), Vang0333s00130 (VaWRKY45), Vang06g17510 (VaWRKY61), Vradi05g10960 (VrWRKY3), Vradi06g02270 (VrWRKY53) and Vradi07g29640 (VrWRKY74) seemed to play a crucial role in biotic stress due to high occurrence of elicitor or fungal responsive elements.

## Discussion

It is well established that classification of genes is not only essential but also a pre-requisite for the functional analysis of a gene family. In general, the gene families of transcription factors which bind DNA in a sequence-specific manner, contain highly conserved characteristic DNA binding domains or motifs, which are crucial for their biological functions. Furthermore, domain is considered as functional as well as an evolutionary unit of the protein, whose coding sequence can be duplicated and undergo recombination. It makes genome-wide analysis of DNA-binding domains of transcription factor pre-eminent. WRKY TFs are one of the largest families of transcriptional regulators in commonly found in terrestrial plants constitute the signaling cascades modulating many plant processes. Over an investigation of twenty years, we have learned much about WRKY transcription factors. Many *WRKY* genes have been identified, classified and characterized in *A*. *thaliana*, *O*. *sativa*^50^, *Cucumis sativus*^51^, *Gossypium raimondii*^52^, *Solanum lycopersicum*^53^, *Populus trichocarpa*^54^, *Brachypodium distachyon*^55,56^, *Vitis vinifera*^57^, etc. Also, the study of WRKY TFs in legume being gradually revolutionized with the advent of the sequenced genomes. Few model legume crops like *Lotus japonica*, *Medicago truncatula*^45^, and *Phaseolus vulgaris*^58^, have been investigated for WRKY proteins. Recently, the public release of the genome sequence of two major Asian legume crops, Mung bean (*Vigna* *radiata*) and Adzuki bean (*Vigna* *angularis*), released in 2014 and 2015, respectively has made the genomic and functional study of these crops, practicable. In our current study, we analyzed of 91 WRKY proteins in *Vigna angularis* and 85 WRKY proteins in *Vigna radiata*.

It can be elucidated from Fig. 1, that no correlation exists between the number of *WRKY* genes and the size of various crop genome. Although, the number of *WRKY* genes is proportional to the genome size in case of the three legume crops, viz. *V*. *angularis*, *V*. *radiata and L*. *japonica*. Furthermore, there are differences in the distribution of *WRKY* genes in respective groups or subgroup (except subgroup IIx), especially in Group III, among various species (Fig. 1). However, the percentage gene distribution among groups/subgroups, shows that the distribution of *WRKY* genes in closely related *Vigna* species, i.e. Adzuki bean and Mung bean is highly comparable (Fig. 1B). In most of the dicotyledonous legume crops, the Group I or Group IIc hold the most substantial number of members. In case of Adzuki bean and Mung bean, the Group IIc is the largest. However, in rice (monocot), the Group III possessing 36 members represents the largest group, indicating that evolution is more active in Group III and may be the members have more function in monocots. Such variation in the distribution of *WRKY* genes among dicots and monocots suggests that Group III members have been evolved independently after the dissection of monocots and dicots.

The average gene length of *VaWRKY* and *VrWRKY* was found to be 2.8 kb and 3 kb, respectively. Any intron conserved in the gene is considered ancient intron. Most of the WRKY domains contains two types of introns with conserved splicing positions, known as R-type intron and V-type intron. In our study, we found the V-type introns in Group IIa and Group IIb members and the R-type introns in rest of the group members (Supplementary Fig. S2). The above finding indicates that WRKY gene family encoding WRKY domain, was formed due to duplication of ancient genes carrying an intron, followed by divergence instead of formation of similar genes as a result of convergence events. The N-terminal WRKY domain is surprisingly intron-less. Although some other group members also suffered a loss in introns, perhaps during evolution.

Because of their substantial contribution to various physiological processes, it is likely that the WRKY family in angiosperms has expanded dramatically during evolution. Recent gene duplication events have been reported to be more prevalent in the expansion of *WRKY* genes in many crops like *Arabidopsis*, Rice^50^, *Populus trichocarpa*^54^, etc. However, in some cases, for instance, in *Lotus Japonica*^45^, recent duplications seemed to play no significant role in *WRKY* gene expansion. In our case also, we found few tandem and segmental gene duplications in Adzuki bean, but not that significant as compared to *Arabidopsis* and rice. The recent gene duplications still need to be study in Mung bean.

The motif analysis by MEME revealed interesting facts regarding the gene evolution. The conserved motif 4 of Group I proteins, occurring just before the WRKYGQK residue containing motif 1 of the C-terminal WRKY domain, were also found in Group IIc proteins (Fig. 6). It indicates that the Group IIc genes have been originated from the loss of the N-terminal WRKY domain of Group I genes, which is also evident by the phylogeny of WRKY domain regions (Supplementary Fig. S2). Moreover, the typical zinc-finger type Group IIc similar to that of Group I CTWD, and the clustering of the Group I CTWD and Group IIc domains in the same clade, further confirm the evolution pattern. The phylogenetic closeness and the conserved V-type intron of Group Ia and Group IIb indicate their evolution from a common origin. The Group IId and Group IIe also seemed to share a common ancestor. Similarly, the phylogenetic relationships between Group III domains and Group I-NTWD suggest their common origination (Section 3.4). Thus, our data support the theory of evolution of *WRKY* genes that the Group I is the oldest group, and Group II and Group II have been evolved from Group^59^.

The *cis*-acting regulatory elements present in the promoter regions are important molecular switches involved in the transcriptional regulation of a gene via controlling an extensive network of gene involved in various biological phenomenon including stress responses and developmental processes. Furthermore, it is evident that defined *cis*-elements can successfully contribute to the genome-wide screening of ABA and abiotic stress-responsive genes^60^. The identification of prominent *cis*-regulatory elements in the promoter region of Adzuki bean and Mung bean genes suggest the putative involvement of the respective genes in environmental stresses like drought, salinity, heat, osmotic stress, senescence, ABA signaling as well as pathogen resistance. Interestingly, these elements were also found in the *WRKY* genes of *Arabidopsis* and Rice, reported to be involved in various abiotic and biotic stress. In *Arabidopsis* and Rice, fusion genes containing a C-terminal WRKY motif and a NBS-LRR (nucleotide binding site-leucine-rich repeat) motif in the *R* gene were identified^50^. The *R* gene mainly confers resistance against pathogens. However similar to *Lotus Japonica*, no such fusion gene was observed in Adzuki bean, as well as Mung bean.

## Methods

### VaWRKY and VrWRKY family identification in Adzuki bean and Mung bean

#### Data resources

The raw protein sequence file and coding sequence file of Adzuki bean and Mung bean was downloaded from the database of Crop Genomics Lab (http://plantgenomics.snu.ac.kr/). The WRKY domain sequence of *Lotus japonica* was downloaded from Plant Transcription Factor Database 3.0 (http://planttfdb.cbi.pku.edu.cn/)^61^. The WRKY protein sequences of *Arabidopsis*, rice, soy bean and *Lotus japonica*, used as reference in this study, were also retrieved from Plant Transcription Factor Database 3.0.

#### Gene identification

The BLAST 2.2.31+ suite (downloaded from ftp://ftp.ncbi.nlm.nih.gov/blast/executables/blast +/LATEST/)^62^, was employed to search through the raw protein sequence file of Adzuki bean and Mung bean respectively, using a *Lotus japonica* WRKY domain (LjWRKY) sequence as query, to survey putative VaWRKY and VrWRKY protein sequences. Out of those five distinct putative VaWRKY protein sequences (Vang01g03800.1, Vang05g09440.1, Vang04g12730.1, Vang06g17510.1 and Vang10g06010.1) and VrWRKY protein sequences (Vradi06g01200.1, Vradi06g01560.1, Vradi03g06620.1, Vradi10g06090.1 and Vradi0183s00040.1) were further chosen as queries to carry out subsequent searches to ensure the identification of all the possible WRKY members. The non-overlapping sequences obtained from the BLAST+ search were subjected to Pfam database search (http://pfam.xfam.or/) and SMART database search (http://smart.embl-heidelberg.de/) for the confirmation of WRKY domains^63,64^. The sequences having no significant matches with WRKY domain in the Pfam and SMART search were eliminated. Although some members possessing truncated but significant portion of the typical WRKY domain were retained in the study. To estimate the theoretical pI and molecular weight of the VaWRKY and VrWRKY proteins, Compute pI/Mw tool (http://web.expasy.org/compute_pi/) was used, with the resolution set to ‘average’. After the domain identification, the gene sequences encoding the corresponding VaWRKY and VrWRKY proteins were retrieved using the Jbrowse tool of Crop Genomics Lab database.

#### Exon-intron organization and chromosome location

To illustrate the exon-intron organization of the genes, Gene Structure Display Server 2.0 (http://gsds.cbi.pku.edu.cn/) was used^65^. The tool required the gene sequences and corresponding coding sequences as input. The phylogenetic tree of the genes belonging to different groups, created by MEGA 6.0^66^, was also uploaded with the gene and coding sequences, to generate the gene structure. Chromosome location of the *VaWRKY* genes was determined using the Vigna Genome Server (http://viggs.dna.affrc.go.jp/)^67^. The physical location of the genes was documented manually.

#### VaWRKY and VrWRKY protein classification, phylogenetic reconstruction and conserved motif analysis

To classify the VaWRKY and VrWRKY proteins in their respective groups, combination of two approaches, multiple sequence alignment and phylogenetic analysis were employed. First, the domain sequences were aligned by Clustal Omega (http://www.ebi.ac.uk/Tools/ msa/clustalo/) using default settings^68^, and the conserved amino acid residues were displayed with GeneDoc software^69^. The domains were screened manually to classify them in respective groups and subgroups, based on their typical zinc-finger-motif and sequence similarity. Subsequently, the phylogenetic tree of VaWRKY and VrWRKY proteins, along with LjWRKY used as reference, were created using Clustal Omega to confirm the classification^68^. To further support the protein classification, phylogenetic analysis of the WRKY domain sequences was also performed. After classification the WRKY domains were analyzed with MEME suite 4.11.1 (http://meme-suite.org/tools/meme), for the identification of conserved motifs, with optimum search parameters as: minimum motif width = 6; maximum motif width = 50; maximum number of motif = 20; minimum sites per motif = 2; maximum sites per motif-600)^44^.

#### RNA sequence data analysis

The raw RNA sequence data of Adzuki bean and Mung bean (SRR1652394, SRR3406553, SRR1407784 and SRR1653637) were downloaded from NCBI Sequence Read Archive and their SRA files were saved in FASTQ format^46–49^. To acquire quality reads, we performed quality trimming and adapter removal of raw sequencing reads by Trimmomatic 0.32 with the following options: ILLUMINACLIP:adapters.fa: 2:30:10 TRAILING:20 MINLEN:25^70^. The reads passed through above quality filtering steps were aligned on the reference genome of Adzuki bean and Mung bean (downloaded from Crop Genomics Lab database) using TopHat v2.1.0^71^. The aligned reads in each sample were used to provide the fragments per kilobase of exon per million fragments mapped (FPKM) values by Cufflinks v2.2.1^72^. The FPKM values were log2 transformed and represented as expression level in the histogram. All the FPKM values were added with a pseudocount of 1 to avoid the negative log2 transformation values in the histogram.

#### Prediction of putative stress-responsive VaWRKY and VrWRKY and their promoter analysis

Using the reference of six AtWRKY, five OsWRKY and two GmWRKY proteins reported to be involved in various abiotic and biotic stresses^21–37^, phylogenetic trees of VaWRKY and VrWRKY protein sequences were created with Clustal Omega tool. The proteins closely clustered to the reference WRKYs in the tree, were selected as stress-responsive homologs in Adzuki bean and Mung bean. The 1.5 kb upstream promoter region sequence from the transcription start site of the homologous *VaWRKY* and *VrWRKY* genes were retrieved using the ‘Jbrowse’ tool of Crop Genomics Lab database (http://plantgenomics.snu.ac.kr/) in order to perform promoter analysis. To investigate the stress-responsive *cis*-elements present in the 1.5 kb upstream region of the promoters, two different tools PlantCare (http://bioinformatics.psb.ugent.be/webtools/plantcare/html/) and PLACE (https://sogo.dna.affrc.go.jp/cgi-bin/sogo.cgi) were used^73,74^. The *cis*-elements identified were then mapped at their respective positions manually.

## Electronic supplementary material

S1- S11

## Data Availability

All the raw sequences analyzed in this study were retrieved from the database of Crop Genomics Lab (http://plantgenomics.snu.ac.kr/). The sequence names (*Vang###*) and (*Vradi###*) have been replaced with the nomenclature *VaWRKY##* and *VrWRKY##* respectively. The description of promoter *cis*-elements mentioned in this paper are available in the database of PlantCare (http://bioinformatics.psb.ugent.be/webtools/plantcare/html/) and PLACE (https://sogo.dna.affrc.go.jp/cgi-bin/sogo.cgi), and in Supplementary Information available.
