# Supplementary material for: Comparative genome-wide analysis of WRKY transcription factors in two Asian legume crops: Adzuki bean and Mung bean
Source: Sci Rep. 2018 Nov 19;8:16971. doi: 10.1038/s41598-018-34920-8 (PMC6243003; doi:10.1038/s41598-018-34920-8)
Supplement: Supplementary file 1 — S1- S11 [file 41598_2018_34920_MOESM1_ESM.doc]

**SUPPLEMENTARY FILES**

**Comparative genome-wide analysis of WRKY transcription factors in two Asian legume crops: Adzuki bean and Mung bean**

Richa Srivastava^1^, Sanjeev Kumar^1^, Yasufumi Kobayashi^2^, Kazutaka Kusunoki^3^, Prateek Tripathi^4^, Yuriko Kobayashi^3^, Hiroyuki Koyama^3^, and Lingaraj Sahoo^1*^

^1^Department of Biosciences and Bioengineering, Indian Institute of Technology Guwahati, Guwahati, 781039, India,

^2^Japan International Research Center for Agricultural Sciences Biological Resources Post-Harvest Division, 1-1 Ohwashi, Tsukuba, Ibaraki, 305-8686, Japan,

^3^Faculty of Applied Biological Sciences, Gifu University, 1-1, Yanagido, 501-1193, Gifu, Japan,

^4^Department of Cell and Molecular Biology, The Scripps Research Institute, La Jolla, CA USA, 92037

* Tel: +91361-258-2204. Fax: +91361-258-2249. Email: ls@iitg.ernet.in

| **Supplementary Table S1| List of VaWRKY and VrWRKY proteins and their physical properties** | | | | | | | | | | | | | | | |
| --- | --- | --- | --- | --- | --- | --- | --- | --- | --- | --- | --- | --- | --- | --- | --- |
| **Protein name** | **Protein ID** | **Domain identification** | | | **Physical properties** | | |  |  | **Domain identification** | | | **Physical properties** | | |
|  |  | **Pfam Clan: CL0274** | | **SMART ID: SM000774** | **pI** | **Mol Wt. (Da)** | **Length (aa)** | Protein name | **Protein ID** | **Pfam Clan: CL0274** | | **SMART ID: SM000774** | **pI** | **Mol Wt. (Da)** | **Length (aa)** |
|  |  | **Start-End Position** | **Bit score** | **Start-End Position** |  |  |  |  |  | **Start-End Position** | **Bit score** | **Start-End Position** |  |  |  |
| **VaWRKY1** | Vang09g01780.1 | 158-213  368-423 | 61.3  80.4 | 156-214  366-425 | 8.71 | 55473.35 | 499 | **VrWRKY1** | Vradi03g09710.1 | 150-205  359-404 | 63.6  80.3 | 148-206  357-416 | 8.61 | 54500.13 | 490 |
| **VaWRKY2** | Vang11g11810.1 | 194-249  366-387 | 63.3  30.0 | 192-250  364-399 | 9.27 | 43873.30 | 399 | **VrWRKY2** | Vradi07g21330.1 | 194-249  366-385  418-455 | 62.7  27.9  40.2 | 192-250  364-457 | 8.98 | 58069.26 | 537 |
| **VaWRKY3** | Vang06g11490.1 | 214-255  532-589 | 67.6  81.8 | 213-268  531-598 | 6.37 | 83627.68 | 737 | **VrWRKY3** | Vradi05g10960.1 | 146-202  319-377 | 87.4  76.1 | 144-204  318-379 | 7.11 | 54000.89 | 485 |
| **VaWRKY4** | Vang0103s00010.1 | 199-255  349-406 | 84.4  88.4 | 198-256  348-407 | 6.46 | 54939.11 | 494 | **VrWRKY4** | Vradi08g13900.1 | 208-229  480-537 | 30.1  82.0 | 207-241  479-528 | 6.38 | 77028.82 | 676 |
| **VaWRKY5** | Vang04g08110.1 | 137-193  306-363 | 88.0  85.3 | 136-194  305-364 | 8.30 | 53345.08 | 476 | **VrWRKY5** | Vradi06g01200.1 | 242-300  374-433 | 85.0  91.6 | 243-299  375-432 | 8.24 | 59920.00 | 552 |
| **VaWRKY6** | Vang03g12780.1 | 122-178  246-284 | 89.2  47.0 | 121-179  302-321 | 6.68 | 42771.38 | 388 | **VrWRKY6** | Vradi10g02560.1 | 227-283  351-408 | 85.3  91.4 | 226-284  350-409 | 7.68 | 58012.46 | 533 |
| **VaWRKY7** | Vang01g03800.1 | 139-195  311-368 | 90.2  91.2 | 138-196  310-369 | 8.09 | 52996.02 | 479 | **VrWRKY7** | Vradi05g21980.1 | 320-376  536-593 | 86.9  87.0 | 319-377  535-594 | 5.83 | 80934.65 | 746 |
| **VaWRKY8** | Vang04g14650.1 | 198-252  395-452 | 88.5  89.0 | 196-254  394-453 | 8.68 | 52701.63 | 476 | **VrWRKY8** | Vradi07g06660.1 | 122-178  263-320 | 83.0  92.1 | 121-179  262-321 | 6.77 | 41310.70 | 372 |
| **VaWRKY9** | Vang04g04330.1 | 91-146  303-306 | 86.6  86.3 | 90-148  302-361 | 6.36 | 62756.96 | 567 | **VrWRKY9** | Vradi0100s00250.1 | 183-203  245-302 | 24.9  92.1 | 182-237  244-303 | 8.83 | 38709.06 | 357 |
| **VaWRKY10** | Vang01g01490.1 | 213-269  389-226 | 84.9  91.6 | 212-270  388-447 | 6.69 | 61416.04 | 566 | **VrWRKY10** | Vradi04g09020.1 | 224-280  395-452 | 87.2  92.7 | 223-281  394-453 | 8.54 | 55439.94 | 508 |
| **VaWRKY11** | Vang03g14810.1 | 248-298  366-423 | 85.3  91.3 | 241-299  365-424 | 8.05 | 59895.82 | 548 | **VrWRKY11** | Vradi06g06770.1 | 233-289  405-462 | 89.8  90.8 | 232-290  404-463 | 7.23 | 62664.69 | 573 |
| **VaWRKY12** | Vang0039ss00390.1* | 320-376  536-593 | 86.9  87.0 | 319-377  535-594 | 5.83 | 80893.60 | 746 | **VrWRKY12** | Vradi0158s00480.1 | 206-262  365-422 | 88.6  90.7 | 205-263  364-423 | 6.66 | 58010.30 | 526 |
| **VaWRKY12** | Vang0039ss00390.2* | 320-376  536-593 | 86.9  87.0 | 319-377  535-594 | 5.83 | 80893.60 | 746 | **VrWRKY13** | Vradi0261s00010.1 | 197-253  346-403 | 84.4  88.4 | 196-254  345-404 | 6.13 | 54652.80 | 491 |
| **VaWRKY13** | Vang0397s00030.1 | 162-288  311-368 | 80.8  91.8 | 161-219  310-369 | 7.00 | 46218.09 | 420 | **VrWRKY14** | Vradi0417s00050.1 | 200-256  369-426 | 87.8  85.1 | 199-257  368-427 | 7.21 | 59517.7 | 533 |
| **VaWRKY14** | Vang04g00270.1 | 224-280  413-470 | 87.1  92.6 | 223-281  412-471 | 7.70 | 57590.23 | 526 | **VrWRKY15** | Vradi03g03190.1 | 196-250  412-468 | 88.5  89.0 | 194-252  410-469 | 8.54 | 54202.20 | 492 |
| **VaWRKY15** | Vang01g00540.1* | 122-178  297-353 | 75.0  90.0 | 121-179  296-355 | 8.29 | 54607.49 | 500 | **VrWRKY16** | Vradi06g00500.1 | 114-170  289-345 | 75.0  90.0 | 113-171  288-347 | 6.97 | 55207.02 | 506 |
| **VaWRKY15** | Vang01g00540.2* | 122-178  297-353 | 75.0  90.0 | 121-179  296-355 | 8.29 | 54607.49 | 500 | **VrWRKY17** | Vradi06g13520.1 | 108-165 | 81.1 | 107-167 | 8.61 | 30421.71 | 268 |
| **VaWRKY16** | Vang0027ss00340.1 | 161-219 | 86.0 | 160-220 | 9.08 | 34336.59 | 307 | **VrWRKY18** | Vradi07g15410.1 | 255-312 | 88.6 | 254-314 | 5.49 | 66806.65 | 615 |
| **VaWRKY17** | Vang01g15570.1 | 113-176 | 54.0 | 131-278 | 7.56 | 28918.60 | 259 | **VrWRKY19** | Vradi0349s00020.1 | 146-204 | 88.0 | 145-205 | 8.02 | 51905.52 | 481 |
| **VaWRKY18** | Vang05g09440.1 | 146-202 | 86.9 | 144-204 | 7.10 | 31013.74 | 277 | **VrWRKY20** | Vradi11g10580.1 | 161-219 | 88.8 | 160-220 | 7.62 | 57647.56 | 526 |
| **VaWRKY19** | Vang01g15560.1 | 108-165 | 81.1 | 107-167 | 8.32 | 30370.59 | 268 | **VrWRKY21** | Vradi08g16000.1 | 2-60 | 89.2 | 1-61 | 9.28 | 28534.22 | 249 |
| **VaWRKY20** | Vang05g09490.1 | 113-172 | 76.3 | 112-173 | 8.40 | 31518.35 | 279 | **VrWRKY22** | Vradi06g11150.1 | 108-166 | 85.9 | 107-167 | 7.63 | 25443.77 | 234 |
| **VaWRKY21** | Vang0033ss01130.1 | 140-198 | 88.8 | 139-199 | 8.20 | 52742.22 | 480 | **VrWRKY23** | Vradi06g07040.1 | 119-177 | 83.8 | 118-178 | 8.96 | 27404.81 | 245 |
| **VaWRKY22** | Vang0065s00590.1 | 149-188 | 60.9 | 148-212 | 8.46 | 46844.40 | 425 | **VrWRKY24** | Vradi07g05680.1 | 137-195 | 88.8 | 136-196 | 7.98 | 52287.33 | 474 |
| **VaWRKY23** | Vang1037s00010.1 | 143-208 | 82.1 | 142-209 | 8.21 | 51440.84 | 468 | **VrWRKY25** | Vradi0401s00040.1 | 202-259 | 87.1 | 200-260 | 8.49 | 40272.65 | 366 |
| **VaWRKY24** | Vang04g05450.1 | 192-250 | 90.2 | 191-255 | 7.70 | 58279.23 | 531 | **VrWRKY26** | Vradi07g23970.1 | 157-213 | 85.0 | 155-215 | 7.60 | 36282.91 | 330 |
| **VaWRKY25** | Vang11g15810.1 | 142-198 | 85.1 | 140-200 | 8.12 | 36090.70 | 325 | **VrWRKY27** | Vradi0335s00020.1 | 304-360 | 91.5 | 302-362 | 6.12 | 58166.46 | 524 |
| **VaWRKY26** | Vang09g05580.1 | 269-326 | 86.4 | 267-327 | 8.87 | 56539.24 | 512 | **VrWRKY28** | Vradi06g01560.1 | 118-175 | 93.5 | 116-176 | 6.60 | 37912.49 | 352 |
| **VaWRKY27** | Vang0318s00160.1 | 206-262 | 92.0 | 204-262 | 8.43 | 46700.62 | 524 | **VrWRKY29** | Vradi0222s00030.1 | 126-174 | 62.8 | 126-175 | 9.60 | 24004.07 | 213 |
| **VaWRKY28** | Vang01g02960.1 | 180-237 | 93.2 | 178-238 | 5.30 | 45073.92 | 414 | **VrWRKY30** | Vradi05g22430.1 | 359-416 | 92.0 | 357-417 | 6.07 | 66569.63 | 614 |
| **VaWRKY29** | Vang03g07430.1 | 263-320 | 90.4 | 261-321 | 7.62 | 53754.20 | 493 | **VrWRKY31** | Vradi02g07100.1 | 330-387 | 90.5 | 328-388 | 6.40 | 69831.72 | 634 |
| **VaWRKY30** | Vang0032ss02430.1 | 332-389 | 90.3 | 330-390 | 5.82 | 64350.32 | 591 | **VrWRKY32** | Vradi0048s00100.1 | 250-308 | 92.6 | 249-309 | 5.86 | 54091.20 | 490 |
| **VaWRKY31** | Vang09g01900.1 | 272-329 | 93.0 | 270-330 | 5.69 | 57010.23 | 527 | **VrWRKY33** | Vradi04g08580.1 | 189-247 | 89.9 | 188-248 | 7.62 | 57647.56 | 526 |
| **VaWRKY32** | Vang04g17360.1 | 300-357 | 89.7 | 298-358 | 6.75 | 57320.35 | 530 | **VrWRKY34** | Vradi01g11520.1 | 121-178 | 89.3 | 119-179 | 8.53 | 30420.70 | 277 |
| **VaWRKY33** | Vang06g15530.1 | 188-245 | 88.3 | 186-246 | 7.69 | 44380.94 | 407 | **VrWRKY35** | Vradi0273s00140.1 | 234-291 | 88.1 | 232-292 | 8.34 | 50185.59 | 454 |
| **VaWRKY34** | Vang0322s00110.1 | 145-203 | 93.1 | 144-204 | 6.47 | 42272.92 | 385 | **VrWRKY36** | Vradi0111s00350.1 | 49-69 | 32.1 | 49-69 | 9.37 | 7853.09 | 69 |
| **VaWRKY35** | Vang0051s00140.1 | 140-197 | 83.2 | 139-199 | 5.49 | 34495.70 | 310 | **VrWRKY37** | Vradi07g24510.1 | 34-72 | 49.1 | 30-73 | 5.48 | 34357.45 | 300 |
| **VaWRKY36** | Vang08g01570.1 | 69-125 | 75.7 | 68-126 | 9.45 | 9989.06 | 86 | **VrWRKY38** | Vradi0043s00750.1 | 2-59 | 80.0 | 1-60 | 9.45 | 10013.04 | 86 |
| **VaWRKY37** | Vang04g03920.1 | 105-162 | 89.7 | 104-163 | 9.30 | 21053.94 | 183 | **VrWRKY39** | Vradi07g30190.1 | 16-52 | 59.9 | 9-53 | 6.96 | 8651.79 | 74 |
| **VaWRKY38** | Vang08g00900.1 | 44-104 | 91.2 | 43-102 | 7.00 | 15368.30 | 134 | **VrWRKY40** | Vradi05g05410.1 | 58-115 | 89.4 | 57-116 | 7.83 | 16593.50 | 144 |
| **VaWRKY39** | Vang04g12730.1 | 117-174 | 92.8 | 116-175 | 9.38 | 21610.09 | 195 | **VrWRKY41** | Vradi06g07670.1 | 20-56 | 41.2 | 14-57 | 8.91 | 12094.73 | 109 |
| **VaWRKY40** | Vang0173s00160.1 | 68-124 | 90.9 | 67-125 | 8.82 | 17238.29 | 148 | **VrWRKY42** | Vradi04g07740.1 | 138-195 | 86.9 | 137-196 | 5.90 | 44323.40 | 403 |
| **VaWRKY41** | Vang10g07150.1 | 98-155 | 90.2 | 97-156 | 9.53 | 21151.29 | 177 | **VrWRKY43** | Vradi10g06370.1 | 190-237 | 74.7 | 189-240 | 5.31 | 30179.09 | 271 |
| **VaWRKY42** | Vang0005s00450.1 | 229-286 | 92.2 | 228-287 | 8.46 | 36846.84 | 341 | **VrWRKY44** | Vradi09g05960.1 | 45-64 | 28.7 | 45-64 | 5.13 | 7417.18 | 64 |
| **VaWRKY43** | Vang05g03980.1 | 101-146 | 66.7 | 100-150 | 5.28 | 20072.94 | 176 | **VrWRKY45** | Vradi01g10680.1 | 156-212 | 88.5 | 155-214 | 9.34 | 26552.91 | 232 |
| **VaWRKY44** | Vang01g17410.1 | 103-170 | 67.5 | 102-171 | 5.14 | 20274.13 | 173 | **VrWRKY46** | Vradi0043s00400.1 | 77-133 | 89.7 | 76-135 | 9.54 | 17953.54 | 153 |
| **VaWRKY45** | Vang0333s00130.1 | 43-100 | 93.1 | 42-101 | 9.54 | 16379.90 | 141 | **VrWRKY47** | Vradi03g06620.1 | 282-339 | 91.8 | 281-340 | 8.31 | 47530.93 | 415 |
| **VaWRKY46** | Vang01g02180.1 | 193-250 | 91.7 | 192-251 | 7.40 | 39175.18 | 343 | **VrWRKY48** | Vradi0100s00500.1 | 148-205 | 90.2 | 147-206 | 6.67 | 35418.50 | 319 |
| **VaWRKY47** | Vang0005s00190.1 | 157-214 | 90.3 | 156-215 | 6.11 | 33730.52 | 305 | **VrWRKY49** | Vradi0048s00470.1 | 43-100 | 93.4 | 42-101 | 9.63 | 15074.42 | 130 |
| **VaWRKY48** | Vang04g17060.1 | 113-170 | 90.3 | 112-171 | 8.90 | 28384.59 | 247 | **VrWRKY50** | Vradi04g07130.1 | 105-162 | 89.7 | 104-163 | 9.19 | 20953.74 | 183 |
| **VaWRKY49** | Vang11g16350.1 | 194-251 | 94.6 | 193-252 | 6.06 | 35014.65 | 323 | **VrWRKY51** | Vradi09g03960.1 | 62-118 | 80.3 | 61-119 | 9.57 | 17453.87 | 151 |
| **VaWRKY50** | Vang10g04840.1 | 175-231 | 88.3 | 174-233 | 9.36 | 28733.48 | 251 | **VrWRKY52** | Vradi0146s00260.1 | 181-249 | 69.8 | 180-250 | 9.53 | 30417.47 | 270 |
| **VaWRKY51** | Vang0942s00010.1* | 139-195 | 88.7 | 138-197 | 8.48 | 25131.58 | 215 | **VrWRKY53** | Vradi06g02270.1 | 175-232 | 91.8 | 173-233 | 7.69 | 37068.81 | 325 |
| **VaWRKY51** | Vang0942s00010.2* | 77-133 | 89.7 | 78-135 | 9.54 | 17953.54 | 153 | **VrWRKY54** | Vradi01g10590.1 | 88-145 | 79.8 | 87-146 | 8.47 | 18720.55 | 167 |
| **VaWRKY52** | Vang08g06450.1* | 156-222 | 69.3 | 155-224 | 7.63 | 27175.87 | 239 | **VrWRKY55** | Vradi06g13730.1 | 103-126 | 32.2 | 102-138 | 4.74 | 16749.43 | 139 |
| **VaWRKY52** | Vang08g06450.2* | 130-186 | 88.1 | 129-188 | 8.86 | 23092.34 | 203 | **VrWRKY56** | Vradi05g11580.1 | 116-159 | 65.2 | 115-162 | 5.41 | 18564.44 | 162 |
| **VaWRKY52** | Vang08g06450.3* | 69-125 | 89.2 | 68-127 | 8.75 | 16848.83 | 142 | **VrWRKY57** | Vradi08g08840.1 | 178-235 | 95.6 | 176-236 | 9.81 | 27128.89 | 244 |
| **VaWRKY53** | Vang08g06450.4* | 18-74 | 90.7 | 17-76 | 8.82 | 10950.21 | 91 | **VrWRKY58** | Vradi11g04520.1 | 182-238 | 95.8 | 180-240 | 9.65 | 27829.53 | 254 |
| **VaWRKY53** | Vang10g03000.1 | 91-148 | 79.3 | 90-149 | 9.54 | 16379.90 | 141 | **VrWRKY59** | Vradi07g10760.1 | 195-252 | 93.5 | 194-254 | 9.40 | 28869.65 | 267 |
| **VaWRKY54** | Vang0605s00070.1 | 2-59 | 80.0 | 1-60 | 9.45 | 9989.06 | 86 | **VrWRKY60** | Vradi07g22750.1 | 81-138 | 97.7 | 79-139 | 9.18 | 17617.12 | 158 |
| **VaWRKY55** | Vang07g02340.1 | 189-245 | 95.7 | 187-247 | 9.85 | 28680.65 | 261 | **VrWRKY61** | Vradi05g02540.1 | 225-281 | 95.1 | 223-283 | 9.77 | 32281.86 | 295 |
| **VaWRKY56** | Vang11g04110.1 | 246-302 | 90.3 | 244-303 | 9.61 | 34057.86 | 317 | **VrWRKY62** | Vradi06g12190.1 | 171-227 | 94.1 | 169-229 | 9.72 | 27201.29 | 248 |
| **VaWRKY57** | Vang11g13040.1 | 252-309 | 95.8 | 250-310 | 9.99 | 35541.24 | 329 | **VrWRKY63** | Vradi05g09450.1 | 117-173 | 95.9 | 115-175 | 9.30 | 20961.79 | 191 |
| **VaWRKY58** | Vang09g06970.1 | 114-170 | 96.6 | 112-172 | 9.66 | 19952.80 | 184 | **VrWRKY64** | Vradi01g14060.1 | 212-268 | 88.8 | 210-270 | 6.49 | 43306.57 | 397 |
| **VaWRKY59** | Vang05g08140.1 | 143-199 | 95.0 | 141-201 | 9.72 | 24229.76 | 218 | **VrWRKY65** | Vradi04g07100.1 | 76-123 | 50.1 | 75-124 | 5.68 | 27497.98 | 253 |
| **VaWRKY60** | Vang01g12760.1 | 167-223 | 94.1 | 165-225 | 8.80 | 26762.54 | 244 | **VrWRKY66** | Vradi09g04070.1 | 79-136 | 93.3 | 77-137 | 5.01 | 26512.03 | 245 |
| **VaWRKY61** | Vang06g17510.1 | 248-305 | 95.0 | 246-306 | 9.90 | 34924.74 | 314 | **VrWRKY67** | Vradi0048s00350.1 | 236-293 | 90.0 | 234-294 | 5.96 | 51217.14 | 480 |
| **VaWRKY62** | Vang0013ss00970.1* | 189-208 | 26.2 | 187-230 | 9.96 | 33074.96 | 294 | **VrWRKY68** | Vradi10g06090.1 | 257-314 | 90.2 | 255-315 | 5.78 | 47228.04 | 433 |
| **VaWRKY62** | Vang0013ss00970.2* | 198-217 | 26.2 | 196-239 | 9.90 | 34052.11 | 303 | **VrWRKY69** | Vradi07g30880.1 | 134-192 | 90.2 | 133-193 | 5.60 | 34103.09 | 301 |
| **VaWRKY63** | Vang0304s00120.1 | 78-134 | 89.5 | 76-136 | 5.65 | 28092.38 | 255 | **VrWRKY70** | Vradi0023s00350.1 | 78-129 | 72.9 | 76-136 | 6.19 | 21733.34 | 196 |
| **VaWRKY64** | Vang07g06530.1 | 137-192 | 84.1 | 135-196 | 9.31 | 23770.18 | 211 | **VrWRKY71** | Vradi0161s00550.1 | 77-95 | 27.1 | 75-118 | 9.22 | 20362.99 | 182 |
| **VaWRKY65** | Vang10g06430.1 | 138-196 | 90.2 | 137-197 | 5.60 | 34368.47 | 304 | **VrWRKY72** | Vradi03g06560.1 | 57-114 | 88.6 | 55-115 | 4.96 | 29537.60 | 258 |
| **VaWRKY66** | Vang06g08640.1 | 228-248 | 24.6 | 226-268 | 8.68 | 40630.75 | 359 | **VrWRKY73** | Vradi11g01720.1 | 119-178 | 84.7 | 118-180 | 6.20 | 39511.18 | 347 |
| **VaWRKY67** | Vang02g05830.1 | 133-189 | 88.1 | 131-191 | 7.89 | 34727.11 | 318 | **VrWRKY74** | Vradi07g29640.1 | 133-192 | 93.3 | 132-194 | 5.40 | 41060.74 | 361 |
| **VaWRKY68** | Vang08g01000.1 | 18-75 | 92.9 | 16-76 | 5.16 | 20286.25 | 185 | **VrWRKY75** | Vradi0183s00040.1 | 130-189 | 90.4 | 129-191 | 5.62 | 38778.31 | 348 |
| **VaWRKY69** | Vang0322s00040.1 | 165-222 | 90.6 | 163-223 | 5.22 | 39853.11 | 361 | **VrWRKY76** | Vradi0083s00100.1 | 129-188 | 93.2 | 128-190 | 5.31 | 41819.66 | 371 |
| **VaWRKY70** | Vang03g08430.1 | 214-271 | 90.5 | 212-272 | 5.34 | 41838.43 | 381 | **VrWRKY77** | Vradi09g03480.1 | 129-188 | 91.3 | 128-190 | 4.95 | 40538.34 | 360 |
| **VaWRKY71** | Vang04g16950.1 | 78-135 | 88.5 | 76-136 | 5.29 | 31615.19 | 376 | **VrWRKY78** | Vradi05g05160.1 | 163-223 | 88.3 | 162-224 | 5.94 | 35663.76 | 320 |
| **VaWRKY72** | Vang0228s00230.1 | 166-221 | 88.3 | 160-222 | 6.00 | 35370.44 | 318 | **VrWRKY79** | Vradi0214s00230.1 | 152-212 | 80.4 | 151-213 | 6.52 | 36546.06 | 317 |
| **VaWRKY73** | Vang08g00330.1 | 129-188 | 91.2 | 128-190 | 4.96 | 40697.43 | 362 | **VrWRKY80** | Vradi0214s00140.1 | 136-196 | 80.0 | 135-197 | 6.82 | 32525.80 | 284 |
| **VaWRKY74** | Vang03g09520.1 | 113-154 | 51.5 | 106-156 | 5.02 | 37915.24 | 337 | **VrWRKY81** | Vradi04g05450.1 | 114-173 | 89.5 | 113-174 | 5.46 | 32088.81 | 282 |
| **VaWRKY75** | Vang07g06810.1 | 126-185 | 82.4 | 125-187 | 5.74 | 39408.84 | 350 | **VrWRKY82** | Vradi09g05200.1 | 75-134 | 80.2 | 74-136 | 5.46 | 28110.04 | 242 |
| **VaWRKY76** | Vang10g06010.1 | 133-192 | 93.3 | 132-194 | 5.39 | 41007.61 | 361 | **VrWRKY83** | Vradi0338s00040.1 | 138-193 | 62.5 | 137-195 | 6.27 | 36062.45 | 320 |
| **VaWRKY77** | Vang0340s00050.1 | 118-171 | 59.4 | 117-175 | 7.15 | 33830.77 | 300 | **VrWRKY84** | Vradi0338s00060.1 | 181-216 | 32.0 | 182-218 | 4.89 | 33210.32 | 298 |
| **VaWRKY78** | Vang0228s00170.1 | 124-184 | 84.4 | 123-185 | 5.10 | 32536.22 | 285 | **VrWRKY85** | Vradi05g05170.1 | 124-184 | 84.8 | 123-185 | 5.82 | 32879.66 | 286 |
| **VaWRKY79** | Vang0459s00030.1 | 128-187 | 78.5 | 127-189 | 6.51 | 33463.63 | 290 |  | | | | | | | |
| **VaWRKY80** | Vang0459s00020.1 | 152-212 | 79.1 | 151-213 | 7.60 | 36249.94 | 318 |  |  |  |  |  |  |  |  |
| **VaWRKY81** | Vang0352s00010.1 | 108-157 | 45.4 | 108-158 | 6.63 | 28054.80 | 245 |  |  |  |  |  |  |  |  |
| **VaWRKY82** | Vang0459s00010.1 | 133-193 | 78.1 | 132-194 | 5.74 | 33922.91 | 299 |  |  |  |  |  |  |  |  |
| **VaWRKY83** | Vang04g07160.1 | 51-89 | 45.6 | 42-90 | 5.04 | 22024.55 | 196 |  |  |  |  |  |  |  |  |
| **VaWRKY84** | Vang1880s00010.1 | 16-62 | 51.8 | 14-63 | 9.30 | 8841.97 | 75 |  |  |  |  |  |  |  |  |
| ***Splice-variants** | | | | | | | | | | | | | | | |

**
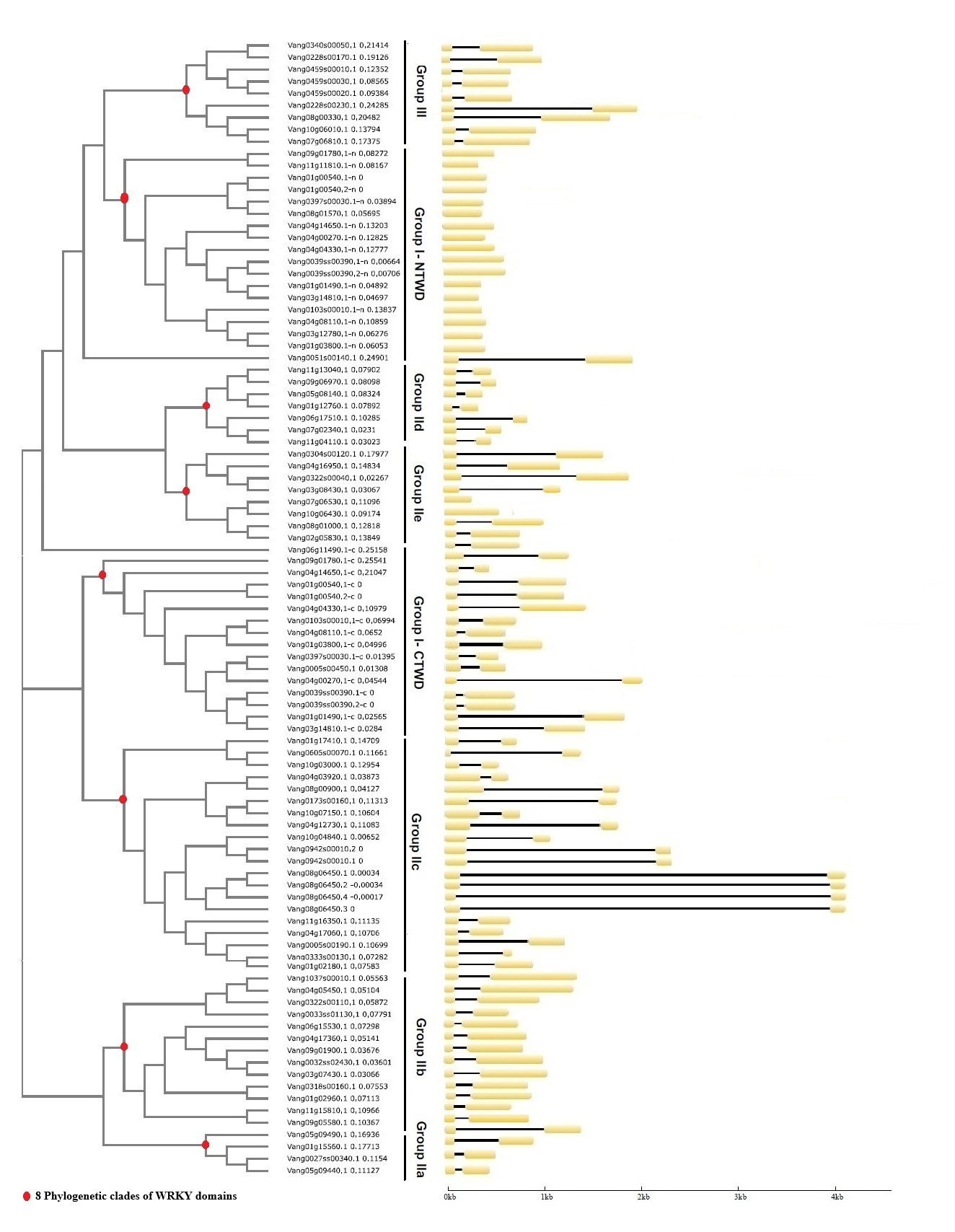
**

**Supplementary Fig. S2a| Phylogenetic relationship and exon-intron structure of VaWRKY domains.** The multiple sequence alignment and the tree construction of the VaWRKY domains (approx. 60 aa) were performed using Clustal Omega. The arrangement of the coding region and introns of each gene was displayed using Gene Structure and Display Server 2.0 program. The truncated domains have been excluded from this study.

**
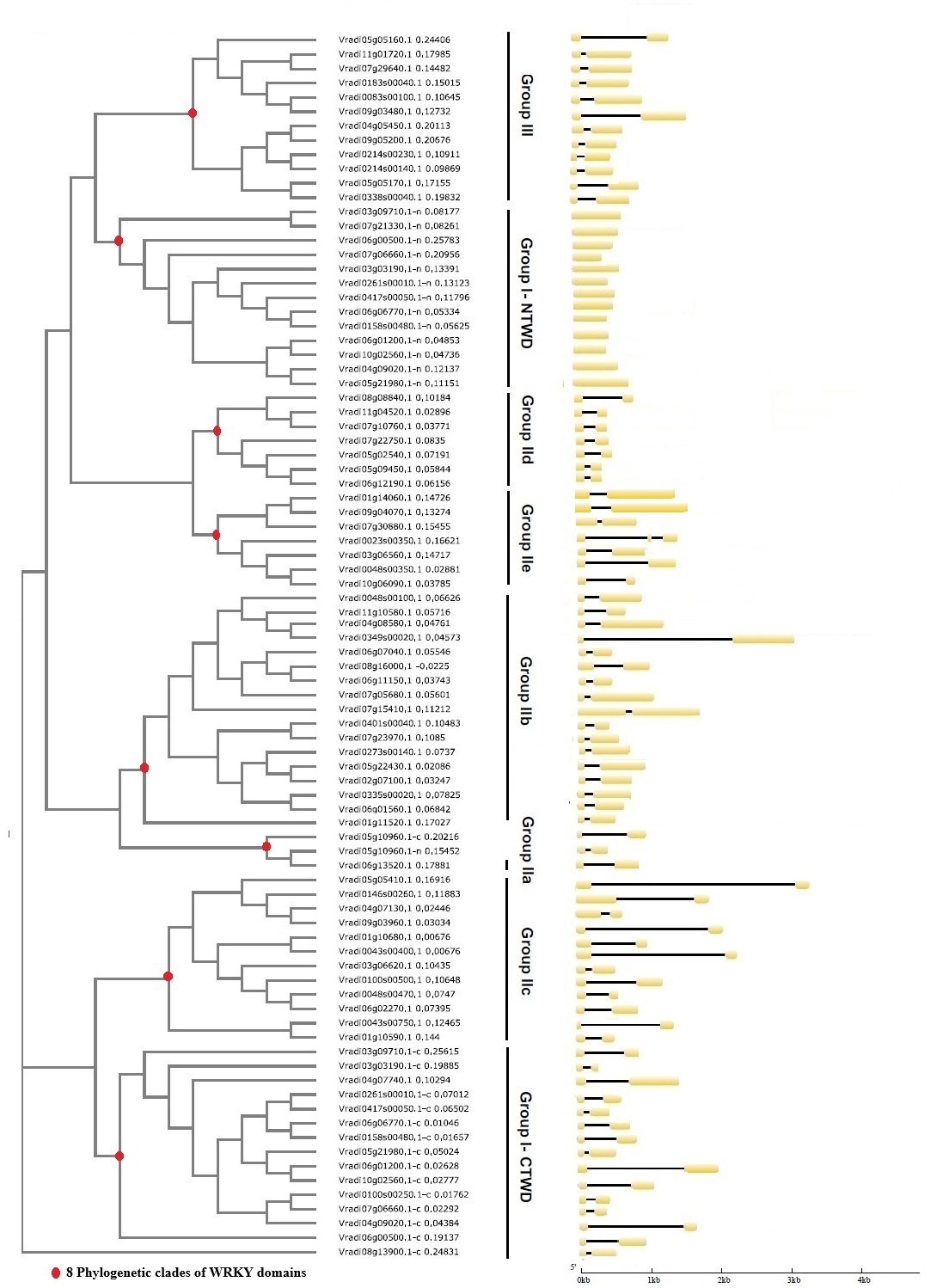
**

**Supplementary Fig. S2b| Phylogenetic relationship and exon-intron structure of VrWRKY domains. The multiple sequence alignment and the tree construction of the VrWRKY domains (approx. 60 aa) were performed using Clustal Omega. The arrangement of the coding region and introns of each gene was displayed using Gene Structure and Display Server 2.0 program. The truncated domains have been excluded from this study.**

| **Supplementary Table S3| Chromosome location of *VaWRKY* and *VrWRKY* genes** | | | | | | | | | |
| --- | --- | --- | --- | --- | --- | --- | --- | --- | --- |
| ***VaWRKY* genes** | | | | | ***VrWRKY* genes** | | | | |
| **Gene name** | **Gene ID** | **Gene locus** | **Intron** | **Gene size (bp)** | **Gene name** | **Gene ID** | **Gene locus** | **Intron** | **Gene size (bp)** |
| **VaWRKY1** | Vang09g01780 | Chr02:37218309..37221695 | 3 | 3389 | **VrWRKY1** | Vradi03g09710 | Chr03:12049994..12053704 | 3 | 3711 |
| **VaWRKY2** | Vang11g11810 | Chr01:55102773..55105558 | 3 | 2786 | **VrWRKY2** | Vradi07g21330 | Chr07:44013217..44019345 | 5 | 6129 |
| **VaWRKY3** | Vang06g11490 | Chr08:13743972..13748760 | 8 | 9828 | **VrWRKY3** | Vradi05g10960 | Chr05:19884728..19897401 | 6 | 12674 |
| **VaWRKY4** | Vang0103s00010 | Chr09:14442219..14439275 | 4 | 2945 | **VrWRKY4** | Vradi08g13900 | Chr08:33603447..33613285 | 8 | 9839 |
| **VaWRKY5** | Vang04g08110 | Chr04:14666001..14673965 | 4 | 7965 | **VrWRKY5** | Vradi06g01200 | Chr06:1177998..1183457 | 5 | 5460 |
| **VaWRKY6** | Vang03g12780 | Chr05:14714865..14717101 | 2 | 2237 | **VrWRKY6** | Vradi10g02560 | Chr10:7173020..7178856 | 4 | 5837 |
| **VaWRKY7** | Vang01g03800 | Chr03:12032243..12039220 | 4 | 6978 | **VrWRKY7** | Vradi05g21980 | Chr05:33473431..33478079 | 4 | 4649 |
| **VaWRKY8** | Vang04g14650 | Chr04:44768498..44764194 | 3 | 6645 | **VrWRKY8** | Vradi07g06660 | Chr07:15154448..15156783 | 3 | 2336 |
| **VaWRKY9** | Vang04g04330 | Chr04:6570518..6571501 | 2 | 3230 | **VrWRKY9** | Vradi0100s00250 | Scaffold100:1199068..1202996 | 4 | 3929 |
| **VaWRKY10** | Vang01g01490 | Chr03:1671516..1666113 | 6 | 5404 | **VrWRKY10** | Vradi04g09020 | Chr04:17736178..17740801 | 2 | 4624 |
| **VaWRKY11** | Vang03g14810 | Chr05:19194928..19189136 | 4 | 5793 | **VrWRKY11** | Vradi06g06770 | Chr06:9218315..9221669 | 4 | 3355 |
| **VaWRKY12** | Vang0039ss00390 | Chr07:24981801..24986508 | 4 | 4690 | **VrWRKY12** | Vradi0158s00480 | Scaffold158:335835..338533 | 4 | 2699 |
| **VaWRKY13** | Vang0397s00030 | Chr01:36073989..36076406 | 3 | 2418 | **VrWRKY13** | Vradi0261s00010 | Scaffold261:13921..16901 | 4 | 2981 |
| **VaWRKY14** | Vang04g00270 | Chr04:4134919..4135542 | 3 | 4947 | **VrWRKY14** | Vradi0417s00050 | Scaffold417:62626..65852 | 4 | 3227 |
| **VaWRKY15** | Vang01g00540 | Chr03:752636..756435 | 3 | 3800 | **VrWRKY15** | Vradi03g03190 | Chr03:4464565..4470921 | 5 | 6357 |
| **VaWRKY16** | Vang0027ss00340 | Chr07:409010..410992 | 4 | 1983 | **VrWRKY16** | Vradi06g00500 | Chr06:450619..454469 | 3 | 3851 |
| **VaWRKY17** | Vang01g15570 | Chr03:38218876..38220114 | 3 | 1239 | **VrWRKY17** | Vradi06g13520 | Chr06:32662124..32664123 | 3 | 2000 |
| **VaWRKY18** | Vang05g09440 | Chr02:5572194..5570792 | 3 | 1403 | **VrWRKY18** | Vradi07g15410 | Chr07:35704217..35708114 | 3 | 3898 |
| **VaWRKY19** | Vang01g15560 | Chr03:38262187..38263833 | 3 | 1647 | **VrWRKY19** | Vradi0349s00020 | Scaffold349:184970..190152 | 5 | 5183 |
| **VaWRKY20** | Vang05g09490 | Chr02:5408957..5405172 | 3 | 3786 | **VrWRKY20** | Vradi11g10580 | Chr11:14939425..14941436 | 2 | 2012 |
| **VaWRKY21** | Vang0033ss01130 | Chr01:30022266..30023645 | 2 | 1947 | **VrWRKY21** | Vradi08g16000 | Chr08:36347891..36349133 | 2 | 1243 |
| **VaWRKY22** | Vang0065s00590 | Chr10:7762855..7760841 | 2 | 2033 | **VrWRKY22** | Vradi06g11150 | Chr06:26619353..26622182 | 3 | 2830 |
| **VaWRKY23** | Vang1037s00010 | Chr10:7893875..7891860 | 2 | 2016 | **VrWRKY23** | Vradi06g07040 | Chr06:9935525..9942117 | 3 | 6593 |
| **VaWRKY24** | Vang04g05450 | Chr04:4854359..4851439 | 3 | 2921 | **VrWRKY24** | Vradi07g05680 | Chr07:12051816..12053641 | 2 | 1826 |
| **VaWRKY25** | Vang11g15810 | Chr01:63160173..63164052 | 4 | 3880 | **VrWRKY25** | Vradi0401s00040 | Scaffold401:45116..49870 | 5 | 4755 |
| **VaWRKY26** | Vang09g05580 | Chr02:40755744..40752943 | 4 | 4788 | **VrWRKY26** | Vradi07g23970 | Chr07:47172847..47175859 | 5 | 3013 |
| **VaWRKY27** | Vang0318s00160 | Chr04:17401097..17406594 | 5 | 5507 | **VrWRKY27** | Vradi0335s00020 | Scaffold335:80281..83380 | 5 | 3100 |
| **VaWRKY28** | Vang01g02960 | Chr03:2193972..2191664 | 4 | 2309 | **VrWRKY28** | Vradi06g01560 | Chr06:1619826..162164 | 3 | 1818 |
| **VaWRKY29** | Vang03g07430 | Chr05:6515638..6512696 | 5 | 2943 | **VrWRKY29** | Vradi0222s00030 | Scaffold222:338915..340669 | 3 | 1755 |
| **VaWRKY30** | Vang0032ss02430 | Chr06:28579675..28576904 | 4 | 2772 | **VrWRKY30** | Vradi05g22430 | Chr05:34422469..34425333 | 4 | 2865 |
| **VaWRKY31** | Vang09g01900 | Chr07:6905048..6907991 | 4 | 2944 | **VrWRKY31** | Vradi02g07100 | Chr02:8383911..8389543 | 6 | 5633 |
| **VaWRKY32** | Vang04g17360 | Chr04:49894390..49899379 | 5 | 4990 | **VrWRKY32** | Vradi0048s00100 | Scaffold048:607031..609659 | 4 | 2629 |
| **VaWRKY33** | Vang06g15530 | Chr08:16809249..16811865 | 5 | 2617 | **VrWRKY33** | Vradi04g08580 | Chr04:17085203..17087730 | 3 | 2528 |
| **VaWRKY34** | Vang0322s00110 | Chr01:18058741..18061318 | 3 | 2578 | **VrWRKY34** | Vradi01g11520 | Chr01:22564179..22565404 | 3 | 1226 |
| **VaWRKY35** | Vang0051s00140 | Chr04:27052399..27050079 | 2 | 2321 | **VrWRKY35** | Vradi0273s00140 | Scaffold273:323293..326284 | 6 | 2992 |
| **VaWRKY36** | Vang08g01570 | Chr09:28492030..28492428 | 0 | 399 | **VrWRKY36** | Vradi0111s00350 | Scaffold111:1254943..1255494 | 1 | 552 |
| **VaWRKY37** | Vang04g03920 | Chr04:7021772..7020805 | 1 | 968 | **VrWRKY37** | Vradi07g24510 | Chr07:47830812..47838404 | 5 | 7593 |
| **VaWRKY38** | Vang08g00900 | Chr09:30120184..30122880 | 2 | 2725 | **VrWRKY38** | Vradi0048s00470 | Scaffold048:1766809..1768816 | 3 | 2008 |
| **VaWRKY39** | Vang04g12730 | Chr04:32781295..32783427 | 1 | 2133 | **VrWRKY39** | Vradi07g30190 | Chr07:54050988..54051787 | 1 | 800 |
| **VaWRKY40** | Vang0173s00160 | Chr02:31042460..31044220 | 1 | 1760 | **VrWRKY40** | Vradi05g05410 | Chr05:10149687..10153024 | 1 | 3338 |
| **VaWRKY41** | Vang10g07150 | Chr01:4382510..4381631 | 1 | 880 | **VrWRKY41** | Vradi06g07670 | Chr06:11859953..11861072 | 1 | 1120 |
| **VaWRKY42** | Vang0005s00450 | Chr10:4569588..4566259 | 3 | 4904 | **VrWRKY42** | Vradi04g07740 | Chr04:15972873..15976848 | 2 | 3976 |
| **VaWRKY43** | Vang05g03980 | Chr02:4701884..4697600 | 3 | 4289 | **VrWRKY43** | Vradi10g06370 | Chr10:13652131..13654202 | 3 | 2072 |
| **VaWRKY44** | Vang01g17410 | Chr03:38555446..38554340 | 2 | 1107 | **VrWRKY44** | Vradi09g05960 | Chr09:8593651..8594931 | 1 | 1281 |
| **VaWRKY45** | Vang0333s00130 | Chr01:19723269..19725302 | 3 | 2034 | **VrWRKY45** | Vradi01g10680 | Chr01:21055071..21056836 | 1 | 1766 |
| **VaWRKY46** | Vang01g02180 | Chr03:2842709..2844884 | 2 | 2176 | **VrWRKY46** | Vradi0043s00400 | Scaffold043:1035360..1038892 | 2 | 3533 |
| **VaWRKY47** | Vang0005s00190 | Chr10:4995043..4998436 | 3 | 6710 | **VrWRKY47** | Vradi03g06620 | Chr03:8043878..8045831 | 2 | 1954 |
| **VaWRKY48** | Vang04g17060 | Chr04:50275622..50276916 | 2 | 1295 | **VrWRKY48** | Vradi0100s00500 | Scaffold100:808511..815675 | 4 | 7165 |
| **VaWRKY49** | Vang11g16350 | Chr01:63883293..63881680 | 2 | 1614 | **VrWRKY49** | Vradi0043s00750 | Scaffold043:938987..940837 | 1 | 1851 |
| **VaWRKY50** | Vang10g04840 | Chr01:6378783..6380480 | 2 | 1698 | **VrWRKY50** | Vradi04g07130 | Chr04:15114825..15115494 | 1 | 670 |
| **VaWRKY51** | Vang0942s00010* | Chr02:23475172..23479007 | 2 or 3 | 3836 | **VrWRKY51** | Vradi09g03960 | Chr09:5364043..5366405 | 2 | 2363 |
| **VaWRKY52** | Vang08g06450* | Chr09:1784046..1789294 | 1,2,or 3 | 5249 | **VrWRKY52** | Vradi0146s00260 | Scaffold146:180462..182378 | 1 | 1917 |
| **VaWRKY53** | Vang10g03000 | Chr01:6512026..6512895 | 2 | 870 | **VrWRKY53** | Vradi06g02270 | Chr06:2215784..2218450 | 2 | 2667 |
| **VaWRKY54** | Vang0605s00070 | Chr02:24445184..24446924 | 1 | 1793 | **VrWRKY54** | Vradi01g10590 | Chr01:20930755..20931865 | 2 | 1111 |
| **VaWRKY55** | Vang07g02340 | Chr10:23636706..23634474 | 3 | 2233 | **VrWRKY55** | Vradi06g13730 | Chr06:32944162..32944765 | 1 | 604 |
| **VaWRKY56** | Vang11g04110 | Chr01:48536123..48533468 | 2 | 2656 | **VrWRKY56** | Vradi05g11580 | Chr05:20480493..20481310 | 2 | 818 |
| **VaWRKY57** | Vang11g13040 | Chr01:61492703..61494387 | 2 | 1685 | **VrWRKY57** | Vradi08g08840 | Chr08:24882292..24885829 | 3 | 3538 |
| **VaWRKY58** | Vang09g06970 | Chr02:42398095..42396472 | 4 | 1624 | **VrWRKY58** | Vradi11g04520 | Chr11:4295522..4297007 | 3 | 1486 |
| **VaWRKY59** | Vang05g08140 | Chr02:7317175..7315630 | 2 | 1547 | **VrWRKY59** | Vradi07g10760 | Chr07:27970345..27972550 | 3 | 2206 |
| **VaWRKY60** | Vang01g12760 | Chr03:35112687..35115136 | 2 | 2450 | **VrWRKY60** | Vradi07g22750 | Chr07:45789294..45792328 | 3 | 3035 |
| **VaWRKY61** | Vang06g17510 | Chr08:21125636..21128781 | 5 | 3146 | **VrWRKY61** | Vradi05g02540 | Chr05:3081327..3083090 | 2 | 1764 |
| **VaWRKY62** | Vang0013ss00970 | Chr11:26716267..26715368 | 5 | 4746 | **VrWRKY62** | Vradi06g12190 | Chr06:29446169..29448444 | 2 | 2276 |
| **VaWRKY63** | Vang0304s00120 | Chr07:9797344..9799760 | 2 | 2417 | **VrWRKY63** | Vradi05g09450 | Chr05:17522271..17523696 | 3 | 1426 |
| **VaWRKY64** | Vang07g06530 | Chr10:26780200..26780918 | 1 | 719 | **VrWRKY64** | Vradi01g14060 | Chr01:34519445..34521140 | 2 | 1696 |
| **VaWRKY65** | Vang10g06430 | Chr01:3601365..3600124 | 1 | 1242 | **VrWRKY65** | Vradi04g07100 | Chr04:15092625..15094230 | 1 | 1606 |
| **VaWRKY66** | Vang06g08640 | Chr08:6848635..6851749 | 4 | 3115 | **VrWRKY66** | Vradi09g04070 | Chr09:5658865..5660183 | 2 | 1319 |
| **VaWRKY67** | Vang02g05830 | Chr11:28396822..28398059 | 2 | 1238 | **VrWRKY67** | Vradi0048s00350 | Scaffold048:756629..768671 | 3 | 12043 |
| **VaWRKY68** | Vang08g01000 | Chr09:29961948..29962815 | 1 | 868 | **VrWRKY68** | Vradi10g06090 | Chr10:13282065..13284775 | 4 | 2711 |
| **VaWRKY69** | Vang0322s00040 | Chr01:18249106..18252371 | 3 | 3266 | **VrWRKY69** | Vradi07g30880 | Chr07:54761861..54762847 | 1 | 987 |
| **VaWRKY70** | Vang03g08430 | Chr05:8879659..8882240 | 4 | 2582 | **VrWRKY70** | Vradi0023s00350 | Scaffold023:1135923..1138000 | 3 | 2078 |
| **VaWRKY71** | Vang04g16950 | Not determined | 2 | 1923 | **VrWRKY71** | Vradi0161s00550 | Scaffold161:221123..222584 | 3 | 1462 |
| **VaWRKY72** | Vang0228s00230 | Chr02:18468542..18471380 | 2 | 2839 | **VrWRKY72** | Vradi03g06560 | Chr03:7968517..7970262 | 2 | 1746 |
| **VaWRKY73** | Vang08g00330 | Chr09:31044242..31041682 | 2 | 2561 | **VrWRKY73** | Vradi11g01720 | Chr11:1556171..1557365 | 2 | 1195 |
| **VaWRKY74** | Vang03g09520 | Chr05:10291037..10293378 | 1 | 2342 | **VrWRKY74** | Vradi07g29640 | Chr07:53352986..53354945 | 2 | 1960 |
| **VaWRKY75** | Vang07g06810 | Chr10:27012541..27013761 | 2 | 1221 | **VrWRKY75** | Vradi0183s00040 | Scaffold183:292529..293754 | 2 | 1226 |
| **VaWRKY76** | Vang10g06010 | Chr01:3221913..3223866 | 2 | 1954 | **VrWRKY76** | Vradi0083s00100 | Scaffold083:606706..608633 | 2 | 1928 |
| **VaWRKY77** | Vang0340s00050 | Chr03:16320836..16322444 | 2 | 1609 | **VrWRKY77** | Vradi09g03480 | Chr09:4431363..4434359 | 2 | 2997 |
| **VaWRKY78** | Vang0228s00170 | Chr02:18419490..18421844 | 2 | 2357 | **VrWRKY78** | Vradi05g05160 | Chr05:9437732..9440109 | 2 | 2378 |
| **VaWRKY79** | Vang0459s00030 | Chr07:20028820..20027306 | 2 | 1515 | **VrWRKY79** | Vradi0214s00230 | Scaffold214:538619..541393 | 3 | 2775 |
| **VaWRKY80** | Vang0459s00020 | Chr07:20045985..20048538 | 3 | 2554 | **VrWRKY80** | Vradi0214s00140 | Scaffold214:522372..523657 | 2 | 1286 |
| **VaWRKY81** | Vang0352s00010 | Chr07:19894462..19893183 | 1 | 1280 | **VrWRKY81** | Vradi04g05450 | Chr04:11816882..11818656 | 2 | 1775 |
| **VaWRKY82** | Vang0459s00010 | Chr07:20063297..20065122 | 2 | 1826 | **VrWRKY82** | Vradi09g05200 | Chr09:7543813..7545037 | 2 | 1225 |
| **VaWRKY83** | Vang04g07160 | Chr04:12760917..12759301 | 1 | 1615 | **VrWRKY83** | Vradi0338s00040 | Scaffold338:234378..236249 | 2 | 1872 |
| **VaWRKY84** | Vang1880s00010 | Chr09:342728..343448 | 2 | 721 | **VrWRKY84** | Vradi0338s00060 | Scaffold338:242969..248877 | 2 | 5909 |
|  |  |  |  |  | **VrWRKY85** | Vradi05g05170 | Chr05:9448831..9451500 | 2 | 2670 |
| **NOTE***   - **Splice variant with variable number of introns** - **Digits preceding the letter ‘g’ in Gene ID stands for maker no** - **Digits preceding the letter ‘s’ and ‘ss’ in Gene ID stands for scaffold no** | | | | | | | | |  |

| **Supplementary Table S4| Conserved regions in VaWRKY and VrWRKY domains** | | | | | | | |
| --- | --- | --- | --- | --- | --- | --- | --- |
| **VaWRKY domains** | | | | **VrWRKY domains** | | | |
| **Motif** | **Width** | **Site Count** | **Consensus Sequence** | **Motif** | **Width** | **Site Count** | **Consensus Sequence** |
| **Motif 1^1^** | 21 | 85 | YRWRKYGQKVVKGNPYPRSYY | **Motif 1^1^** | 21 | 78 | YRWRKYGQKVVKGNPYPRSYY |
| **Motif 2^1^** | 30 | 75 | CTNAGCPVRKHVERASHDPKAVITTYEGKH | **Motif 2^1^** | 29 | 79 | GCPVRKQVQRCAEDRSILITTYEGNHNHP |
| **Motif 3^1^** | 50 | 15 | DGYNWRKYGQKQVKGSEYPRSYYKCTHPNCQVKKKVERSHEGHITEIIYK | **Motif 3^1^** | 50 | 13 | DDGYNWRKYGQKQVKGSEYPRSYYKCTHPNCQVKKKVERSHEGHITEIIY |
| **Motif 4^1^** | 15 | 47 | RFCFQTRSDVDVLDD | **Motif 4** | 21 | 44 | TVREPRVVVQTTSEIDILDDG |
| **Motif 5** | 29 | 13 | NHPLPPAAMAMASTTTAAASMLLSGSMSS | **Motif 5** | 29 | 17 | LPPAAIAMAQTTSAAARMLLSGSMSSADG |
| **Motif 6^1^** | 41 | 8 | RCTHNNCRVKKRVERLSEDCRMVITTYEGRHNHSPCDDSNS | **Motif 6** | 28 | 14 | TVSAATAAIAADPNFTAALAAAITSIIG |
| **Motif 7** | 29 | 18 | DTVSAATAAIAADPNFTAALAAAITSIIG | **Motif 7^2^** | 41 | 12 | EMVVLQAELERMKVENHRLRNMLDQVNTNYNALQMHLVSIM |
| **Motif 8^2^** | 44 | 16 | ENKLAVLQSELERMKRENQKLRDALHQVNVNYDALQMHFMNLMQ | **Motif 8^3^** | 29 | 7 | CSKKRKSRVKQSIRVPAISSKIADIPPD |
| **Motif 9** | 25 | 13 | QNMATLSASAPFPTITLDLTQNPTN | **Motif 9** | 28 | 10 | PCSQNMATLSASAPFPTITLDLTQNPTN |
| **Motif 10^3^** | 29 | 6 | HCSKRRKSRMKRMIRVPAISSKIADIPAD | **Motif 10^1^** | 16 | 11 | KGSHNHPKPQANKRNS |
| **Motif 11** | 17 | 14 | NHDVPAARASSHVNANA | **Motif 11** | 29 | 4 | VNSDTVPLLSSNNVLYNLNALNFTSSYHH |
| **Motif 12** | 19 | 14 | GTHDHPKPQPSRRYSSGTI | **Motif 12** | 25 | 11 | KAIEEELIKGRDIANQLLEVLVLKS |
| **Motif 13** | 20 | 11 | DEDNLGPEAKRWKGDNENDD | **Motif 13** | 26 | 9 | MLPFAEDLVRKVLCSFTNTLLLLNTD |
| **Motif 14^4^** | 15 | 12 | MKMKKMKARRKVREP | **Motif 14** | 17 | 5 | IVDDGYRWRKYGQKTIK |
| **Motif 15** | 28 | 10 | LPFVEDLVRKVLCSFTNTLLLLNSTNDV | **Motif 15^1^** | 6 | 31 | RCTVAP |
| **Motif 16** | 28 | 8 | MVNQPSTERRAMEEELVKGRDIANQLLE | **Motif 16^1^** | 21 | 8 | GDEFDNDEPDAKRWRLEGENE |
| **Motif 17** | 41 | 2 | TDMQVDNPEHVELHNGGDGDIGWGNVQKGNISGAANWKHE | **Motif 17** | 31 | 7 | YFAIPPGLSPAELLDSPVLLNSSNILPSPTT |
| **Motif 18** | 41 | 2 | ENLEAREANENRKSARDSDMRYICIFQFLSYVDGGFAYHVA | **Motif 18** | 42 | 4 | ITETFSAPTSVTNSPILDLDILLHKGDFDTDFPFNNPDFF |
| **Motif 19** | 50 | 2 | FSFGMNQQMLSNLAMSGLGHMQAKLPVMPVHPFLAQQQQQSPSNDFGFMM | **Motif 19^4^** | 20 | 8 | KKRKKMPKWKEQVRVKIHNG |
| **Motif 20** | 50 | 2 | WSSYSSYFNSGPLTHKRNHGDYLLNTGHQNQPYLEHLHHPICTSNNNFSQ | **Motif 20** | 29 | 3 | EITQATHHMDSVATPENSSISMEDDDFGQ |
| ^1^Conserved sequences within the WRKY domain, ^2^bZIP motif, ^3^Plant zinc-cluster domain, ^4^NLS | | | | | | | |

**
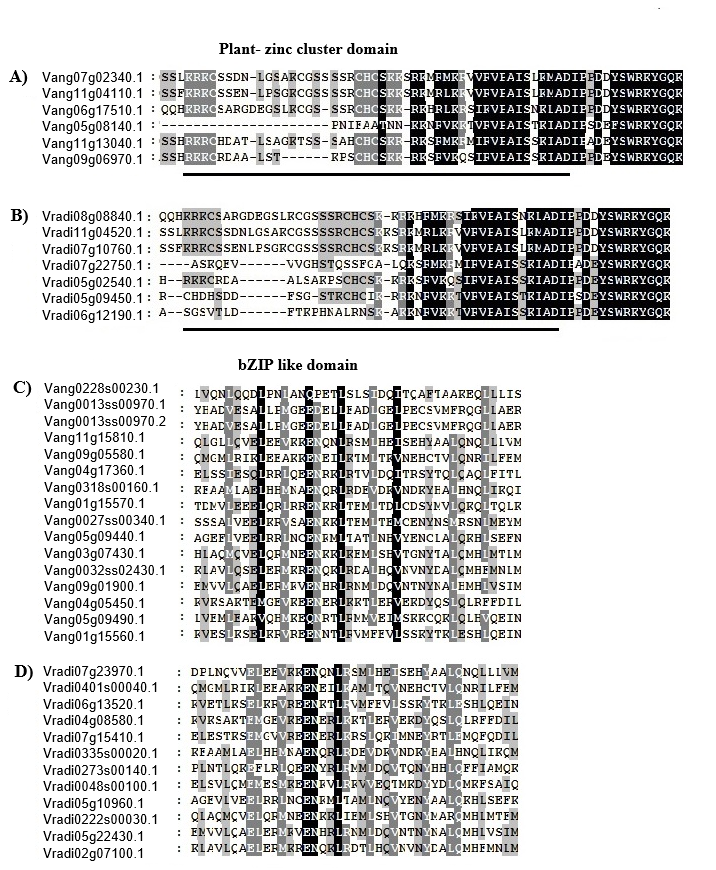
**

**Supplementary Fig. S5| (A) Plant-zinc cluster domain and bZIP-like domain identified in VaWRKY proteins; (B) Plant-zinc cluster domain and bZIP-like domain identified in VrWRKY proteins**. The protein sequences were aligned by clustal omega and the conserved amino acids were represented by GenDoc tool.

| **Supplementary Table S6|** RNA sequence analysis of *VaWRKY* and *VrWRKY* genes | | | | | | | | | | | |
| --- | --- | --- | --- | --- | --- | --- | --- | --- | --- | --- | --- |
|  | **Gene ID** | **FPKM value (Biosample: Root, stem, leaves and flowers)** | | **FPKM value (Biosample: Mature leaves)** | **Group** |  | **Gene ID** | | **FPKM value (Biosample: Root, leaf, stem-15 days)** | **FPKM value (Biosample:**  **Hypocotyl- 5 days**) | **Group** |
| **1** | Vang09g01780 | 3.22 | 1.10 | | **I** | **1** | | Vradi03g09710 | 1.61 | 3.06 | **I** |
| **2** | Vang11g11810 | 17.61 | 4.70 | | **I** | **2** | | Vradi07g21330 | 2.03 | 5.83 | **I** |
| **3** | Vang06g11490 | 0.00 | 0.00 | | **I** | **3** | | Vradi05g10960 | 0.07 | 0.19 | **I** |
| **4** | Vang0103s00010 | 2.83 | 6.65 | | **I** | **4** | | Vradi08g13900 | 0.00 | 0.00 | **I** |
| **5** | Vang04g08110 | 17.10 | 60.29 | | **I** | **5** | | Vradi06g01200 | 2.07 | 12.01 | **I** |
| **6** | Vang03g12780 | 24.75 | 33.23 | | **I** | **6** | | Vradi10g02560 | 2.13 | 9.44 | **I** |
| **7** | Vang01g03800 | 56.68 | 361.33 | | **I** | **7** | | Vradi05g21980 | 0.64 | 8.58 | **I** |
| **8** | Vang04g14650 | 0.85 | 0.06 | | **I** | **8** | | Vradi07g06660 | 0.25 | 0.87 | **I** |
| **9** | Vang04g04330 | 1.36 | 0.12 | | **I** | **9** | | Vradi0100s00250 | 0.21 | 2.73 | **I** |
| **10** | Vang01g01490 | 13.37 | 18.13 | | **I** | **10** | | Vradi04g09020 | 0.92 | 5.79 | **I** |
| **11** | Vang03g14810 | 14.34 | 17.00 | | **I** | **11** | | Vradi06g06770 | 4.84 | 49.97 | **I** |
| **12** | Vang0039ss00390 | 23.12 | 22.74 | | **I** | **12** | | Vradi0158s00480 | 0.03 | 1.16 | **I** |
| **13** | Vang0397s00030 | 1.20 | 1.03 | | **I** | **13** | | Vradi0261s00010 | 0.06 | 2.03 | **I** |
| **14** | Vang04g00270 | 24.75 | 19.04 | | **I** | **14** | | Vradi0417s00050 | 3.14 | 29.52 | **I** |
| **15** | Vang01g00540 | 13.89 | 30.60 | | **I** | **15** | | Vradi03g03190 | 0.81 | 10.52 | **I** |
| **16** | Vang0027ss00340 | 20.61 | 853.44 | | **IIa** | **16** | | Vradi06g00500 | 3.18 | 11.45 | **I** |
| **17** | Vang01g15570 | 2.67 | 35.24 | | **IIa** | **17** | | Vradi06g13520 | 0.17 | 0.61 | **IIa** |
| **18** | Vang05g09440 | 1.98 | 16.08 | | **IIa** | **18** | | Vradi07g15410 | 0.15 | 0.02 | **IIb** |
| **19** | Vang01g15560 | 0.49 | 18.91 | | **IIa** | **19** | | Vradi0349s00020 | 0.02 | 0.01 | **IIb** |
| **20** | Vang05g09490 | 0.00 | 0.09 | | **IIa** | **20** | | Vradi11g10580 | 0.00 | 0.13 | **IIb** |
| **21** | Vang0033ss01130 | 0.00 | 0.00 | | **IIb** | **21** | | Vradi08g16000 | 0.00 | 0.00 | **IIb** |
| **22** | Vang0065s00590 | 0.07 | 0.06 | | **IIb** | **22** | | Vradi06g11150 | 0.00 | 0.22 | **IIb** |
| **23** | Vang1037s00010 | 0.00 | 0.15 | | **IIb** | **23** | | Vradi06g07040 | 0.00 | 0.00 | **IIb** |
| **24** | Vang04g05450 | 0.66 | 0.00 | | **IIb** | **24** | | Vradi07g05680 | 0.00 | 0.00 | **IIb** |
| **25** | Vang11g15810 | 0.14 | 11.59 | | **IIb** | **25** | | Vradi0401s00040 | 0.02 | 0.02 | **IIb** |
| **26** | Vang09g05580 | 3.10 | 10.81 | | **IIb** | **26** | | Vradi07g23970 | 0.00 | 0.06 | **IIb** |
| **27** | Vang0318s00160 | 0.45 | 0.10 | | **IIb** | **27** | | Vradi0335s00020 | 1.18 | 0.66 | **IIb** |
| **28** | Vang01g02960 | 0.04 | 0.00 | | **IIb** | **28** | | Vradi06g01560 | 0.00 | 0.00 | **IIb** |
| **29** | Vang03g07430 | 22.72 | 94.45 | | **IIb** | **29** | | Vradi0222s00030 | 0.00 | 0.02 | **IIb** |
| **30** | Vang0032ss02430 | 0.78 | 1.93 | | **IIb** | **30** | | Vradi05g22430 | 0.44 | 4.35 | **IIb** |
| **31** | Vang09g01900 | 4.06 | 18.31 | | **IIb** | **31** | | Vradi02g07100 | 0.00 | 0.09 | **IIb** |
| **32** | Vang06g15530 | 0.59 | 1.22 | | **IIb** | **32** | | Vradi0048s00100 | 0.34 | 0.04 | **IIb** |
| **33** | Vang04g17360 | 2.66 | 9.42 | | **IIb** | **33** | | Vradi04g08580 | 0.03 | 0.00 | **IIb** |
| **34** | Vang0322s00110 | 0.31 | 1.21 | | **IIb** | **34** | | Vradi01g11520 | 0.00 | 0.00 | **IIb** |
| **35** | Vang0051s00140 | 0.28 | 0.37 | | **IIc** | **35** | | Vradi0273s00140 | 0.01 | 0.03 | **IIb** |
| **36** | Vang08g01570 | 0.00 | 0.00 | | **IIc** | **36** | | Vradi0111s00350 | 0.10 | 0.00 | **IIb** |
| **37** | Vang04g03920 | 1.12 | 0.00 | | **IIc** | **37** | | Vradi07g24510 | 0.41 | 1.32 | **IIc** |
| **38** | Vang08g00900 | 0.83 | 0.00 | | **IIc** | **38** | | Vradi0043s00750 | 0.23 | 0.36 | **IIc** |
| **39** | Vang04g12730 | 1.77 | 17.22 | | **IIc** | **39** | | Vradi07g30190 | 0.00 | 0.91 | **IIc** |
| **40** | Vang0173s00160 | 0.00 | 0.00 | | **IIc** | **40** | | Vradi05g05410 | 0.00 | 0.01 | **IIc** |
| **41** | Vang10g07150 | 1.13 | 4.80 | | **IIc** | **41** | | Vradi06g07670 | 0.00 | 0.00 | **IIc** |
| **42** | Vang0005s00450 | 10.22 | 6.19 | | **IIc** | **42** | | Vradi04g07740 | 0.17 | 1.96 | **IIc** |
| **43** | Vang05g03980 | 0.21 | 0.35 | | **IIc** | **43** | | Vradi10g06370 | 0.02 | 0.22 | **IIc** |
| **44** | Vang01g17410 | 0.11 | 3.45 | | **IIc** | **44** | | Vradi09g05960 | 0.28 | 2.07 | **IIc** |
| **45** | Vang0333s00130 | 0.75 | 0.26 | | **IIc** | **45** | | Vradi01g10680 | 0.88 | 2.23 | **IIc** |
| **46** | Vang01g02180 | 3.54 | 0.74 | | **IIc** | **46** | | Vradi0043s00400 | 0.18 | 0.58 | **IIc** |
| **47** | Vang0005s00190 | 3.71 | 11.46 | | **IIc** | **47** | | Vradi03g06620 | 0.97 | 13.87 | **IIc** |
| **48** | Vang04g17060 | 8.30 | 11.48 | | **IIc** | **48** | | Vradi0100s00500 | 1.80 | 4.93 | **IIc** |
| **49** | Vang11g16350 | 10.91 | 11.18 | | **IIc** | **49** | | Vradi0048s00470 | 0.11 | 1.09 | **IIc** |
| **50** | Vang10g04840 | 2.86 | 1.33 | | **IIc** | **50** | | Vradi04g07130 | 0.00 | 0.30 | **IIc** |
| **51** | Vang0942s00010* | 3.38 | 4.46 | | **IIc** | **51** | | Vradi09g03960 | 0.02 | 0.18 | **IIc** |
| **52** | Vang08g06450* | 13.09 | 0.56 | | **IIc** | **52** | | Vradi0146s00260 | 0.07 | 10.41 | **IIc** |
| **53** | Vang10g03000 | 1.11 | 45.19 | | **IIc** | **53** | | Vradi06g02270 | 0.87 | 10.56 | **IIc** |
| **54** | Vang0605s00070 | 1.32 | 20.40 | | **IIc** | **54** | | Vradi01g10590 | 0.33 | 0.96 | **IIc** |
| **55** | Vang07g02340 | 18.60 | 10.13 | | **IId** | **55** | | Vradi06g13730 | 0.00 | 0.00 | **IIc** |
| **56** | Vang11g04110 | 37.95 | 45.87 | | **IId** | **56** | | Vradi05g11580 | 0.12 | 0.05 | **IIc** |
| **57** | Vang11g13040 | 87.20 | 86.04 | | **IId** | **57** | | Vradi08g08840 | 8.80 | 21.99 | **IId** |
| **58** | Vang09g06970 | 24.17 | 9.82 | | **IId** | **58** | | Vradi11g04520 | 8.91 | 13.50 | **IId** |
| **59** | Vang05g08140 | 8.45 | 8.81 | | **IId** | **59** | | Vradi07g10760 | 20.32 | 36.06 | **IId** |
| **60** | Vang01g12760 | 18.88 | 10.25 | | **IId** | **60** | | Vradi07g22750 | 17.82 | 54.67 | **IId** |
| **61** | Vang06g17510 | 16.52 | 43.08 | | **IId** | **61** | | Vradi05g02540 | 43.93 | 50.16 | **IId** |
| **62** | Vang0013ss00970 | 0.27 | 0.00 | | **IIe** | **62** | | Vradi06g12190 | 10.96 | 17.08 | **IId** |
| **63** | Vang0304s00120 | 8.39 | 26.88 | | **IIe** | **63** | | Vradi05g09450 | 2.90 | 2.69 | **IId** |
| **64** | Vang07g06530 | 0.00 | 0.00 | | **IIe** | **64** | | Vradi01g14060 | 0.10 | 0.20 | **IIe** |
| **65** | Vang10g06430 | 0.21 | 1.08 | | **IIe** | **65** | | Vradi04g07100 | 10.59 | 19.50 | **IIe** |
| **66** | Vang06g08640 | 0.60 | 0.20 | | **IIe** | **66** | | Vradi09g04070 | 0.10 | 0.55 | **IIe** |
| **67** | Vang02g05830 | 0.15 | 1.25 | | **IIe** | **67** | | Vradi0048s00350 | 0.03 | 0.27 | **IIe** |
| **68** | Vang08g01000 | 0.10 | 0.65 | | **IIe** | **68** | | Vradi10g06090 | 0.08 | 0.10 | **IIe** |
| **69** | Vang0322s00040 | 0.17 | 0.00 | | **IIe** | **69** | | Vradi07g30880 | 0.00 | 0.11 | **IIe** |
| **70** | Vang03g08430 | 0.68 | 0.00 | | **IIe** | **70** | | Vradi0023s00350 | 1.48 | 7.16 | **IIe** |
| **71** | Vang04g16950 | 8.43 | 35.59 | | **IIe** | **71** | | Vradi0161s00550 | 1.91 | 0.89 | **IIe** |
| **72** | Vang0228s00230 | 1.24 | 0.00 | | **III** | **72** | | Vradi03g06560 | 2.74 | 19.46 | **IIe** |
| **73** | Vang08g00330 | 15.46 | 86.25 | | **III** | **73** | | Vradi11g01720 | 0.00 | 0.56 | **III** |
| **74** | Vang03g09520 | 31.44 | 95.80 | | **III** | **74** | | Vradi07g29640 | 1.03 | 47.53 | **III** |
| **75** | Vang07g06810 | 0.09 | 0.14 | | **III** | **75** | | Vradi0183s00040 | 0.00 | 0.23 | **III** |
| **76** | Vang10g06010 | 21.53 | 10.73 | | **III** | **76** | | Vradi0083s00100 | 5.94 | 58.28 | **III** |
| **77** | Vang0340s00050 | 0.05 | 0.08 | | **III** | **77** | | Vradi09g03480 | 1.01 | 15.57 | **III** |
| **78** | Vang0228s00170 | 2.84 | 18.55 | | **III** | **78** | | Vradi05g05160 | 0.12 | 0.04 | **III** |
| **79** | Vang0459s00030 | 0.33 | 0.00 | | **III** | **79** | | Vradi0214s00230 | 0.00 | 0.00 | **III** |
| **80** | Vang0459s00020 | 1.10 | 0.00 | | **III** | **80** | | Vradi0214s00140 | 0.00 | 0.03 | **III** |
| **81** | Vang0352s00010 | 0.00 | 0.00 | | **III** | **81** | | Vradi04g05450 | 3.86 | 13.97 | **III** |
| **82** | Vang0459s00010 | 0.11 | 0.00 | | **III** | **82** | | Vradi09g05200 | 0.00 | 0.00 | **III** |
| **83** | Vang04g07160 | 23.53 | 686.79 | | **III** | **83** | | Vradi0338s00040 | 0.00 | 0.00 | **III** |
| **84** | Vang1880s00010 | 0.00 | 0.00 | | **III** | **84** | | Vradi0338s00060 | 0.01 | 0.06 | **III** |
|  |  |  |  | |  | **85** | | Vradi05g05170 | 1.89 | 2.66 | **III** |


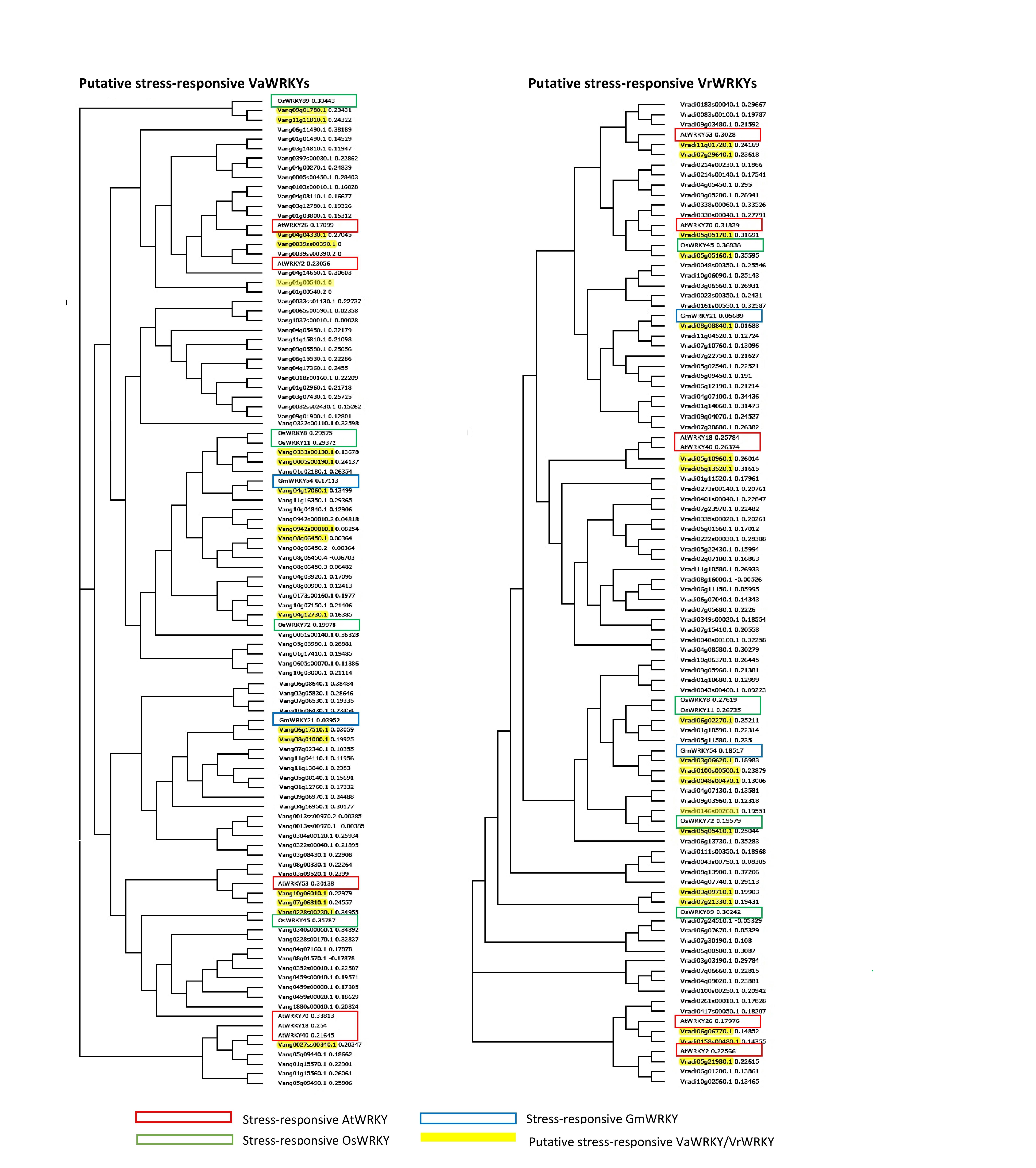


**Supplementary Fig. S7| Selection of putative- stress responsive VaWRKY and VrWRKY candidates homologus to reported stress-responsive AtWRKY, OsWRKY and GmWRKY, based on the phylogenetic relationship.** The tree was created using Clustal Omega. The VaWRKY and VrWRKY members clustered closely to the reference stress-responsive WRKY were chosen as putative homologs in Adzuki bean and Mung bean involved in stress-responses. Additionally, the non-truncated splice- variants of three VaWRKY proteins Vang04g05450, Vang08g06450 and Vang0942s00010 falling in the clade of stress-responsive rich WRKYs were also selected. In total, the subset of 17 *VaWRKY* and *VrWRKY* genes encoding the above selected putative stress-responsive candidates were analyzed for *cis*-regulatory elements in the 1.5 kb upstream promoter region.

| **Supplementary Table S8|** List of abiotic and biotic stress-responsive WRKYs and the stress-responsive *cis*-elements present in 1 kb upstream region of the stress-responsive AtWRKY and OsWRKY | | | | | | |
| --- | --- | --- | --- | --- | --- | --- |
| **Stress-responsive WRKY** | | **Inducer Stress** | **Reported function** | **References** | **Elements predicted by PlantCare** | **Elements predicted by New PLACE (SOGO)** |
| **1** | **AtWRKY2** | NaCl, Mannitol | Negative regulation of ABA signalling | **21, 22** | ARE, ERE, HSE, LTR, MBS**,** TGACG motif | ASF1MOTIFCAMV, ACGTATERD1, ABRELATERD1, DRECRTCOREAT, DPBFCOREDCDC3, MYBCORE, MYB1AT,, LTRE1HVBLT49, LTRECOREATCOR15, WBOXNTERF3 |
| **2** | **AtWRKY26** | Heat, Ethylene | Heat tolerance | **29** | ARE, CGTCA motif, **ERE**, MBS, TCA-element, TGACG motif | ACGTATERD1, ABRELATERD1, CURECORECR, DPBFCOREDCDC3, MYBCORE, MYB1AT, WBOXNTCHN48, WBOXNTERF3 |
| **3** | **OsWRKY89** | Salinity, ABA, and UV-B, Pathogen | Tolerance to UV-B radiation and rice pathogen | **31** | ARE, **CGCTA motif**, HSE, MBS, **TGACG motif** | ACGTATERD1, ASF1MOTIFCAMV, CURECORECR, DPBFCOREDCDC3, MYBCORE, MYB1AT,MYB2AT, MYB2CONSENSUSAT, PREATPRODH, WBOXNTCHN48, WBOXNTERF3 |
| **4** | **AtWRKY18** | ABA, Pathogen | ABA signaling, and negative regulation towards salt and osmotic tolerance, pathogen resistance | **23, 24** | ARE, **BoxW1**, HSE, MBS, TCA element | ACGTATERD1, CURECOREC, ELRECOREPCRP1, LTRECOREATCOR15, MYBCORE, MYB1AT, WBOXNTERF3 |
| **5** | **AtWRKY40** | ABA | Negative regulation of ABA signalling | **21, 22** | **ABRE,** TGACG motif | ACGTATERD1, ABRELATERD1, ASF1MOTIFCAMV, CBFHV, LTRECOREATCOR15, MYCATERD1, MYCATERD22, WBOXNTERF3 |
| **6** | **OsWRKY8** | Drought, Salinity, ABA, Oxidative stress | Tolerance towards osmotic stress | **28** |  |  |
| **7** | **OsWRKY11** | Heat, Drought | Heat and drought tolerance | **30** | ABRE, CGTCA motif, **HSE**, TCA element, TGACG motif | ACGTATERD1, ASF1MOTIFCAMV, CURECOREC,ERELEE4, ELRECOREPCRP1, LTRECOREATCOR15, LTRE1HVBLT49, MYBCORE, MYB1AT,MYB2AT, MYB2CONSENSUSAT, MYCATERD1, MYCATERD22, PREATPRODH, WBOXNTCHN48, WBOXNTERF3 |
| **8** | **OsWRKY72** | ABA | Salt and drought tolerance | **27** | **ABRE**, BoxW1, CGTCA motif, EIRE, LTR, TCA element, TGACG motif | ACGTATERD1, ASF1MOTIFCAMV, CURECORECR, DPBFCOREDCDC3, LTRE1HVBLT49, MYBCORE, MYB1AT,MYB2AT, MYB2CONSENSUSAT, MYCATERD1, MYCATERD22, PREATPRODH, WBOXNTCHN48, WBOXNTERF3 |
| **9** | **GmWRKY54** | Salt, Drought | Salt and drought tolerance | **25** |  |  |
| **10** | **GmWRKY21** | Salt, Drought, Cold | Cold tolerance | **25** |  |  |
| **11** | **AtWRKY53** | NPR1 receptor | Role in senescence, basal defense and negative regulation of drought | **32, 33, 34** | ARE, **BoxW**1,HSE, MBS | CURECOREC, ELRECOREPCRP1, PREATPRODH MYBCORE, MYB1AT, MYB2CONSENSUSAT, MYCATERD1, WBOXNTCHN48, WBOXNTERF3 |
| **12** | **AtWRKY70** | NPR1 receptor | Role in osmotic stress, senescence, and disease resistance | **35, 36** | ARE, ERE, LTR, MBS, TGACG motif ,**Wun motif** | ACGTATERD1, ASF1MOTIFCAMV ,CURECOREC, ERELEE4, PREATPRODH, LTRE1HVBLT49, MYBCORE, MYB1AT, MYB2CONSENSUSAT |
| **13** | **OsWRKY45** | Salt, drought, Cold, Pathogen | Disease resistance, drought and cold tolerance | **26, 37** | ABRE, ARE, **CGTCA motif**, HSE, **MBS**, TCA element, **TGACG motif** | ACGTATERD1,ABRELATERD1, ASF1MOTIFCAMV, CBFHV, CURECORECR, DPBFCOREDCDC3, DRE2COREZMRAB17 , DRECRTCOREAT , LTRECOREATCOR15, MYBCORE, MYB1AT,MYB2AT, MYB2CONSENSUSAT, PREATPRODH, WBOXNTCHN48, WBOXNTERF |
| **Note:** Elements responsive to the reported inducer stress are indicated as **bold** in red font color | | | | | | |

| **Supplementary Table S9|** Stress-responsive *cis* elements present in the promoter region of *VaWRKY* and *VrWRKY* genes | | | | | | | | | | | |
| --- | --- | --- | --- | --- | --- | --- | --- | --- | --- | --- | --- |
| **GeneID** | | **PlantCare** | | **PLACE** | |  | | **PlantCare** | | **PLACE** | |
|  |  | ***Cis*-element** | **Inducer stress** | ***Cis*-element** | **Inducer stress** |  |  | ***Cis*-element** | **Inducer stress** | ***Cis*-element** | **Inducer stress** |
| **1** | **Vang0039ss00390** | ERE  HSE  MBS  TCA-element | Ethylene  Heat-stress  Drought  Salicylic acid | BOXLCOREDCPAL  ERELEE4  MYBCORE, MYB2AT, MYB2CONSENSUSAT  LTRECOREATCOR15  PREATPRODH | UV-B radiation  Ethylene  Drought  Cold-stress  Osmotic stress | 1 | **Vradi05g21980** | ARE  ERE  HSE  LTR  MBS  TCA-element | Oxidative-stress  Ethylene  Heat-stress  Cold  Drought  Salicylic acid | BOXLCOREDCPAL  ERELEE4  MYBCORE, MYB1AT, MYB2AT, MYB2CONSENSUSAT  PREATPRODH  LTRECOREATCOR15 | UV-B radiation  Ethylene  Drought  Osmotic Stress  Cold |
| **2** | **Vang04g04330** | ABRE  CGCTA-motif  TGACG-motif  HSE  LTR  TCA-element | ABA  MeJA  MeJA  Heat stress  Cold  Salicylic acid | ACGTATERD1, MYBCORE  ABRELATERD1  ASF1MOTIFCAMV,  CURECORECR  DPBFCOREDCDC3  ERELEE4  LTRE1HVBLT49  PREATPRODH | Dehydration  Dehydration, ABA  Salicylic acid  Oxygen deficiency  ABA  Ethylene  Cold  Osmotic stress | 2 | **Vradi0158s00480** | ABRE  ARE  Box-W1  MBS  TCA-element  TGACG-motif | ABA  Oxidative-stress  Fungus  Drought  Salicylic acid  MeJA | ACGTATERD1,  ABRELATERD1  ASF1MOTIFCAMV  BOXLCOREDCPAL  CURECORECR  DPBFCOREDCDC3  MYBCORE, MYB1AT, MYB2AT, MYB2CONSENSUSAT  WBOXNTERF3 | Dehydration  Dehydration, ABA  Salicylic acid  UV-B radiation  Oxygen deficiency  ABA  Drought  Wounding |
| **3** | **Vang04g05450** | ABRE  ARE  Box-W1  CGTCA motif  MBS  TCA-element  TGACG-motif | ABA  Oxidative-stress Fungus  MeJA  Drought  Salicylic acid  MeJA | ACGTATERD1, CBFHV  ABRELATERD1, DPBFCOREDCDC3  ASF1MOTIFCAMV  ERELEE4  ELRECOREPCRP1  LTRECOREATCOR15  MYBCORE, MYB1AT, MYB2AT, MYB2CONSENSUSAT | Dehydration  ABA  Salicylic acid  Ethylene  Elicitor  Cold  Drought | 3 | **Vradi06g06770** | ARE  Box-W1  CGCTA-motif  ERE  HSE  MBS  TCA-element  TGACG-motif | Oxidative-stress  Fungus  MeJA  Ethylene  Heat-stress  Drought  Salicylic acid  MeJA | ACGTATERD1  BOXLCOREDCPAL  CURECORECR  DPBFCOREDCDC3  ERELEE4  ELRECOREPCRP1  MYB1AT  WBOXNTERF3 | Dehydration  UV-B radiation  Oxygen deficiency  ABA  Ethylene  Elicitor  Drought  Wounding |
| **4** | **Vang09g01780** | ARE  BoxW1  CGCTA motif  ERE  HSE  MBS  TCA-element  TGACG-motif | Oxidative-stress Fungus  MeJA  Ethylene  Heat  Drought  Salicylic acid  MeJA | ACGTATERD1  ASF1MOTIFCAMV  CURECORECR  ERELEE4  ELRECOREPCRP1  LTRECOREATCOR15  MYBCORE, MYB1AT, MYB2AT, MYB2CONSENSUSAT  WBOXNTERF3 | Dehydration  Salicylic acid  Oxygen deficiency  Ethylene  Elicitor  Cold  Drought  Wounding | 4 | **Vradi03g09710** | ABRE  ARE  Box-W1  EIRE  ERE  HSE  MBS  TCA-element | ABA  Oxidative-stress  Fungus  Elicitor  Ethylene  Heat  Drought  Salicylic acid | ACGTATERD1  ABRELATERD1  DPBFCOREDCDC3  ERELEE4  ELRECOREPCRP1  LTRECOREATCOR15  MYBCORE, MYB1AT, MYB2CONSENSUSAT, MYB2AT  WBOXNTERF3 | Dehydration  Dehydration, ABA  ABA  Ethylene  Elicitor  Cold  Drought  Wounding |
| **5** | **Vang11g11810** | ARE  BoxW1  HSE  LTR  MBS  TCA-element | Oxidative-stress Fungus  Heat  Cold  Drought  Salicylic acid | ACGTATERD1  CURECORECR  DPBFCOREDCDC3  ELRECOREPCRP1  MYBCORE, MYB1AT, MYB2CONSENSUSAT  WBOXNTCHN48  WBOXNTERF3 | Dehydration  Oxygen deficiency  ABA  Elicitor  Drought  Elicitor  Wounding | 5 | **Vradi07g21330** | ABRE  ARE  Box-W1  CGCTA-motif  LTR  TGACG-motif | ABA  Oxidative-stress  Fungus  MeJA  Cold  MeJA | ACGTATERD1  ABRELATERD1  ASF1MOTIFCAMV  CURECORECR  DPBFCOREDCDC3  ELRECOREPCRP1  LTRE1HVBLT49  MYBCORE, MYB1AT, MYCATERD1, MYCATERD22  WBOXNTCHN48  WBOXNTERF3 | Dehydration  Dehydration, ABA  Salicylic acid  Oxidative stress  ABA  Elicitor  Cold  Drought  Elicitor  Wounding |
| **6** | **Vang0027ss00340** | ARE  Box-W1  CGCTA-motif  ERE  TCA-element  TGACG-motif | Oxidative-stress Fungus  MeJA  Ethylene  Salicylic acid  MeJA | ACGTABREMOTIFA2OSEM, DRE2COREZMRAB17  ACGTATERD1, ABRELATERD1, CBFHV  ASF1MOTIFCAMV  CURECORECR  CRTDREHVCBF2, LTRECOREATCOR15, LTREATLTI78  DRECRTCOREAT  ERELEE4  ELRECOREPCRP1  MYB1AT  PREATPRODH  WBOXNTERF3 | ABA  Dehydration  Salicylic acid  Oxygen deficiency  Cold  Drought, salt, cold  Ethylene  Elicitor  Drought  Osmotic stress  Wounding | 6 | **Vradi05g10960** | ABRE  ARE  MBS  TCA element  TGACG-motif | ABA  Oxidative-stress  Drought  Salicylic acid  MeJA | ACGTATERD1, CBFHV  ABRELATERD1  ASF1MOTIFCAMV  BOXLCOREDCPAL  CURECORECR  ERELEE4  ELRECOREPCRP1  MYBCORE, MYB1AT, MYCATERD1, MYCATERD22  WBOXNTERF3 | Dehydration  Dehydration, ABA  Salicylic acid  UV-B radiation  Oxidative stress  Ethylene  Elicitor  Drought  Wounding |
| **7** | **Vang0942s00010** | ABRE  HSE  LTR | ABA  Heat  Cold | ACGTATERD1, ABRELATERD1, CBFHV  BOXLCOREDCPAL  CURECORECR  DPBFCOREDCDC3  LTRE1HVBLT49  MYBCORE, MYB1AT, MYCATRD1, MYCATRD22,  WBOXNTCHN48  WBOXNTERF3 | Dehydration  UV-B radiation  Oxygen deficiency  ABA  Cold  Drought  Elicitor  Wounding | 7 | **Vradi06g13520** | ARE  HSE  MBS  LTR | Oxidative-stress  Heat  Drought  Cold | ACGTATERD1, CBFHV  ABRELATERD1  CURECORECR  DPBFCOREDCDC3  MYBCORE, MYB1AT, MYB2AT, MYCATERD1, MYCATERD22  LTRE1HVBLT49  PREATPRODH  WBOXNTCHN48  WBOXNTERF3 | Dehydration  Dehydration, ABA  Oxidative stress  ABA  Drought  Cold  Osmotic stress  Elicitor  Wounding |
| **8** | **Vang08g06450** | ABRE  ARE  Box-W1  ELI-Box  HSE  MBS  TCA-element  Wun Box | ABA  Oxidative-stress Fungus  Elicitor  Heat  Drought  Salicylic acid  Wounding | ACGTATERD1, ABRELATERD1  CURECORECR  DRE2COREZMRAB17, DPBFCOREDCDC3  ELRECOREPCRP1  MYBCORE, MYB2AT, MYB2CONSENSUSAT, MYCATERD1, MYCATRD22  PREATPRODH  WBOXNTCHN48  WBOXNTERF3 | Dehydration  Oxygen deficiency  ABA  Elicitor  Drought  Osmotic stress  Elicitor  Wounding | 8 | **Vradi07g15410** | ARE  Box-W1  CGCTA motif  HSE  MBS  TCA-element  TGACG-motif | Oxidative-stress  Fungus  MeJA  Heat-stress  Drought  Salicylic acid  MeJA | ACGTATERD1, CBFHV  ASF1MOTIFCAMV  BOXLCOREDCPAL  CRTDREHVCBF2  CURECORECR  DPBFCOREDCDC3  ELRECOREPCRP1  MYBCORE, MYB1AT, MYB2CONSENSUSAT, MYB2AT, MYCATERD1, MYCATERD22  PREATPRODH  WBOXNTERF3 | Dehydration  Salicylic acid  UV-B radiation  Cold  Oxygen deficiency  ABA  Elicitor  Drought  Osmotic stress  Wounding |
| **9** | **Vang0333s00130** | ABRE  ARE  Box-W1  CGCTA-motif  LTR  MBS | ABA  Oxidative-stress Fungus  MeJA  Cold  Drought | ACGTATERD1, ABRELATERD1, CBFHV  ASF1MOTIFCAMV  ACGTABREMOTIFA2OSEM, DPBFCOREDCDC3, ABREATCONSENSUS, ABREATRD22  CURECORECR  DRECRTCOREAT  ELRECOREPCRP1  LTRECOREATCOR15, LTRE1HVBLT49  MYBCORE, MYB2AT, MYB2CONSENSUSAT  PREATPRODH  WBOXNTERF3 | Dehydration  Salicylic acid  ABA  Oxygen deficiency  Drought, salt, cold  Elicitor  Cold  Drought  Osmotic stress  Wounding | 9 | **Vradi0146s00260** | ARE  CGCTA motif  HSE  LTR  TCA-element  TGACG-motif | Oxidative-stress  MeJA  Heat-stress  Cold  Salicylic acid  MeJA | ACGTATERD1, MYB1AT  ABRELATERD1  ASF1MOTIFCAMV  CURECORECR  DPBFCOREDCDC3  ERELEE4  LTRE1HVBLT49 | Dehydration  Dehydration, ABA  Oxidative stress  ABA  Drought  Ethylene  Cold |
| **10** | **Vang0005s00190** | ABRE  ARE  CGTCA motif  ERE  HSE  MBS  TCA-element | ABA  Oxidative-stress MeJA  Ethylene  Heat  Drought  Salicylic acid | ACGTATERD1, ABRELATERD1  ASF1MOTIFCAMV  CURECORECR  DPBFCOREDCDC3  ERELEE4  MYBCORE, MYB1AT, MYB2CONSENSUSAT, MYCATERD1  WBOXNTERF3 | Dehydration  Salicylic acid  Oxygen deficiency  ABA  Ethylene  Drought  Wounding | 10 | **Vradi06g02270** | ABRE  ARE  Box-W1  ERE  HSE  MBS  TCA-element | ABA  Oxidative-stress  Fungus  Ethylene  Heat-stress  Drought  Salicylic acid | ACGTABREMOTIFA2OSEM, DPBFCOREDCDC3  ACGTATERD1  ABRELATERD1  CURECORECR  ERELEE4  ELRECOREPCRP1, WBOXNTCHN48  MYBCORE, MYB1AT, MYB2CONSENSUSAT, MYCATERD1, MYCATERD22  LTRECOREATCOR15  PREATPRODH  WBOXNTERF3 | ABA  Dehydration  Dehydration, ABA  Oxygen deficiency  Ethylene  Elicitor  Drought  Cold  Osmotic stress  Wounding |
| **11** | **Vang04g12730** | ABRE  ARE  CGCTA-motif  HSE  LTR  TCA-element  TGACG-motif | ABA  Oxidative-stress MeJA  Heat  Cold  Salicylic acid  MeJA | ACGTABREMOTIFA2OSEM, DPBFCOREDCDC3  **A**CGTATERD1, ABRELATERD1, MYB1AT  ASF1MOTIFCAMV  CURECORECR  ERELEE4  LTRE1HVBLT49  WBOXNTERF3 | ABA  Dehydration  Salicylic acid  Oxygen deficiency  Ethylene  Cold  Wounding | 11 | **Vradi0048s00470** | ABRE  ARE  Box-W1  CGCTA-motif  LTR  MBS  TCA-element  TGACG-motif | ABA  Oxidative-stress  Fungus  MeJA  Cold  Drought  Salicylic acid  MeJA | ACGTABREMOTIFA2OSEM, ABREATCONSENSUS, ABREATRD22, DPBFCOREDCDC3  ACGTATERD1, CBFHV  ASF1MOTIFCAMV  ABRELATERD1  CURECORECR  DRECRTCOREAT  ELRECOREPCRP1  MYBCORE, MYB1AT, MYB2CONSENSUSAT  MYCATERD1, MYCATERD22  LTRECOREATCOR15, LTRE1HVBLT49  PREATPRODH  WBOXNTERF3 | ABA  Dehydration  Salicylic acid  Dehydration, ABA  Oxygen deficiency  Drought, Salt, Cold  Elicitor  Drought  Cold  Osmotic stress  Wounding |
| **12** | **Vang04g17060** | ABRE  ARE  CE1  HSE  TCA-element | ABA  Oxidative-stress  ABA  Heat  Salicylic acid | ACGTABREMOTIFA2OSEM, ABREATCONSENSUS, ABRECE1HVA22  ACGTATERD1  ABRELATERD1  CURECORECR  CRTDREHVCBF2  MYBCORE, MYB1AT, MYB2CONSENSUSAT, CBFHV  PREATPRODH  WBOXNTERF3 | ABA  Dehydration  Dehydration, ABA  Oxygen deficiency  Cold  Drought  Osmotic stress  Wounding | 12 | **Vradi0100s00500** | ARE  CGCTA-motif  ELI-Box  HSE  TCA-element | Oxidative-stress  MeJA  Elicitor  Heat  Salicylic acid | ACGTATERD1, CBFHV  ASF1MOTIFCAMV  CURECORECR  DPBFCOREDCDC3  ERELEE4  MYB1AT, MYCATERD1, MYCATERD22  WBOXNTERF3 | Dehydration  Salicylic acid  Oxygen deficiency  ABA  Elictor  Drought  Wounding |
| **13** | **Vang06g17510** | ABRE  Box-W1  EIRE  HSE  MBS  Wun Box | ABA  Fungi  Elicitor  Heat stress  Drought  Wounding | ACGTATERD1  CURECORECR  DPBFCOREDCDC3  ELRECOREPCRP1, WBOXNTCHN48  MYBCORE, MYB1AT, MYB2AT, MYB2CONSENSUSAT  PREATPRODH  WBOXNTERF3 | Dehydration  Oxygen deficiency  ABA  Elicitor  Drought  Osmotic stress  Wounding | 13 | **Vradi03g06620** | ABRE  ARE  CGCTA-motif  HSE  MBS  TCA-element  TGACG-motif | ABA  Oxidative-stress  MeJA  Heat  Drought  Salicylic acid  MeJA | ACGTABREMOTIFA2OSEM, DPBFCOREDCDC3, DRE1COREZMRAB17  ACGTATERD1, CBFHV  ABRELATERD1  ASF1MOTIFCAMV  CURECORECR  CRTDREHVCBF2  MYBCORE, MYB1AT, MYB2CONSENSUSAT, MYB2AT  WBOXNTERF3 | ABA  Dehydration  Dehydration, ABA  Salicylic acid  Oxygen deficiency  Cold  Drought  Wounding |
| **14** | **Vang08g01000** | ARE  Box-W1  ERE  MBS  TCA-element | Oxidative stress Fungus  Ethylene  Drought  Salicylic acid | BOXLCOREDCPAL  CURECORECR  ERELEE4  ELRECOREPCRP1  MYBCORE, MYB1AT, MYB2AT, MYB2CONSENSUSAT,  WBOXNTERF3 | UV-B  Oxygen deficiency  Ethylene  Elicitor  Drought  Wounding | 14 | **Vradi08g08840** | ARE  CGCTA-motif  MBS  TCA-element  TGACG-motif | Oxidative-stress  MeJA  Drought  Salicylic acid  MeJA | ACGTATERD1  ASF1MOTIFCAMV  CURECORECR  DPBFCOREDCDC3  MYBCORE, MYB1AT, MYB2CONSENSUSAT, MYB2AT | Dehydration  Salicylic acid  Oxygen deficiency  ABA  Drought |
| **15** | **Vang10g06010** | ARE  CGCTA-motif  HSE  MBS  TCA-element  TGACG-motif | Oxidative-stress  MeJA  Heat  Drought  Salicylic acid  MeJA | ACGTATERD1, ABRELATERD1  ASF1MOTIFCAMV  DPBFCOREDCDC3  MYBCORE, MYB1AT, MYB2CONSENSUSAT, MYCATERD1, MYCATRD22  LTRECOREATCOR15  PREATPRODH  WBOXNTERF3  WBOXNTCHN48 | Dehydration  Salicylic acid  ABA  Drought  Cold  Osmotic stress  Wounding  Elicitor | 15 | **Vradi07g29640** | ARE  HSE  MBS  TCA-element | Oxidative-stress  Heat  Drought  Salicylic acid | ACGTATERD1  ASF1MOTIFCAMV  ABRELATERD1  CURECORECR  DPBFCOREDCDC3  MYBCORE, MYB1AT, MYB2CONSENSUSAT, MYCATERD1, MYCATERD22  PREATPRODH  WBOXNTCHN48  WBOXNTERF3 | Dehydration  Salicylic acid  Dehydration, ABA  Oxygen deficiency  ABA  Drought  Osmotic stress  Elicitor  Wounding |
| 16 | **Vang07g06810** | ARE  CGCTA-motif  HSE  LTR  TCA-element  TGACG-motif | Oxidative-stress MeJA  Heat  Cold  Salicylic acid  MeJA | ACGTABREMOTIFA2OSEM  ACGTATERD1, ABRELATERD1  ASF1MOTIFCAMV  BOXLCOREDCPAL  CURECORECR  MYB1AT, MYCATERD1, MYCATRD22  LTRE1HVBLT49  WBOXNTCHN48  WBOXNTERF3 | ABA  Dehydration  Salicylic acid  UV-B radiation  Oxygen deficiency  Drought  Cold  Elicitor  Wounding | 16 | **Vradi11g01720** | ARE  Box-W1  CGCTA-motif  TCA-element  TGACG-motif | Oxidative-stress  Fungus  MeJA  Salicylic acid  MeJA | ACGTATERD1  ASF1MOTIFCAMV  ABRELATERD1  BOXLCOREDCPAL  CURECORECR  ELRECOREPCRP1  MYB1AT  PREATPRODH  WBOXNTERF3 | Dehydration  Salicylic acid  Dehydration, ABA  UV-B radiation  Oxygen deficiency  Elicitor  Drought  Osmotic stress  Wounding |
| 17 | **Vang0228s00230** | ARE  CGTCA motif  ERE  HSE  LTR  MBS  TCA-element  TGACG-motif | Oxidative-stress MeJA  Ethylene  Heat  Cold  Drought  Salicylic acid  MeJA | ACGTATERD1  ASF1MOTIFCAMV  BOXLCOREDCPAL  CURECORECR  DPBFCOREDCDC3  ERELEE4  MYBCORE, MYB1AT, MYB2AT, MYB2CONSENSUSAT  LTRE1HVBLT49  WBOXNTERF3 | Dehydration  Salicylic acid  Oxygen deficiency  UV-B  ABA  Ethylene  Drought  Cold  Wounding | 17 | **Vradi05g05170** | CGCTA-motif  HSE  MBS  TCA-element  TGACG-motif  Wun Box | MeJA  Heat  Drought  Salicylic acid  MeJA  Wounding | ACGTATERD1, CBFHV  ASF1MOTIFCAMV  ABRELATERD1  CURECORECR  DPBFCOREDCDC3  MYBCORE, MYB2CONSENSUSAT, MYCATERD1, MYCATERD22  PREATPRODH  WBOXNTCHN48  WBOXNTERF3 | Dehydration  Salicylic acid  Dehydration, ABA  Oxygen deficiency  ABA  Drought  Osmotic stress  Elicitor  Wounding |
|  | | | | | | 18 | **Vradi05g05160** | CGCTA-motif  ERE  HSE  LTR  MBS  TCA-element  TGACG-motif | MeJA  Ethylene  Heat  Cold  Drought  Salicylic acid  MeJA | ACGTATERD1  ASF1MOTIFCAMV  BOXLCOREDCPAL  CURECORECR  DPBFCOREDCDC3  ERELEE4  MYBCORE, MYB2CONSENSUSAT, MYB2AT  MYCATERD1, MYCATERD22  PREATPRODH  LTRE1HVBLT49  WBOXNTERF3 | Dehydration  Salicylic acid  UV-B radiation  Oxygen deficiency  ABA  Ethylene  Drought  Osmotic stress  Cold  Wounding |

**Supplementary Table S10:** Positions of stress-responsive cis elements present in the 1.5 kb upstream promoter region of putative stress-responsive (a) *VaWRKY* and (b) *VrWRKY* genes

| **Supplementary Table S10.1a Vang0039ss00390** | | | | | | | |
| --- | --- | --- | --- | --- | --- | --- | --- |
| **PlantCare** | | | | **PLACE** | | | |
|  | ***Cis*-element** | **Sequence** | **Position(strand)** |  | **Cis-element** | **Sequence** | **Position(strand)** |
| **1** | ERE | ATTTCAAA | 1263(-) | **1** | BOXLCOREDCPAL | ACCWWCC | 377(+) |
| **2** | HSE | AAAAAATTTC | 1226(-) | **2** | ERELEE4 | AWTTCAA | 1263(-) |
| **3** | MBS | CAACTG  TAACTG | 650(+)  28(+), 748(+) | **3** | LTRECOREATCOR15 | CCGAC | 759(-) |
|  |  |  |  | **4** | MYBCORE | CNGTTR | 28(-), 414(-), 568(-), 650(-), 709(-), 748(-), 796(-), 799(+) |
| **4** | TCA-element | CCATCTTTTT | 510(-) | **5** | MYB2AT | TAACTG | 28(+), 748(+) |
|  | | | | **6** | MYB2CONSENSUSAT | YAACKG | 28(+), 650(+), 748(+), 799(-) |
|  |  |  |  | **7** | PREATPRODH | ACTCAT | 886(+) |

| **Supplementary Table S10.2a Vang04g04330** | | | | | | | |
| --- | --- | --- | --- | --- | --- | --- | --- |
| **PlantCare** | | | | **PLACE** | | | |
|  | **Cis-element** | **Sequence** | **Position(strand)** |  | **Cis-element** | **Sequence** | **Position(strand)** |
| **1** | ABRE | TACGTG | 967(+) | **1** | ACGTATERD1 | ACGT | 156(-), 156(+), 968(-), 968(+) |
| **2** | CGCTA-motif  TGACG-motif | CGTCA  TGACG | 1091(+)  1091(-) | **2** | ABRELATERD1 | ACGTG | 156(+), 968(+) |
|  |  |  |  | **3** | ASF1MOTIFCAMV | TGACG | 1091(-) |
| **3** | HSE | AAAAAATTTC | 14(-), 489(+) | **4** | CURECORECR | GTAC | 397(-), 397(+), 588(-), 588(+) |
| **4** | LTR | CCGAAA | 77(+), 201(+) | **5** | DPBFCOREDCDC3 | ACACNNG | 157(-) |
| **5** | TCA-element | CCATCTTTTT | 6(+), 469(+) | **6** | ERELEE4 | AWTTCAA | 507(+), 1233(+) |
|  | | | | **7** | MYBCORE | CNGTTR | 3(-) |
|  |  |  |  | **8** | LTRE1HVBLT49 | CCGAAA | 77(+), 201(+) |
|  |  |  |  | **9** | PREATPRODH | ACTCAT | 1308(+) |

| **Supplementary Table S10.3a Vang04g05450** | | | | | | | |
| --- | --- | --- | --- | --- | --- | --- | --- |
| **PlantCare** | | | | **PLACE** | | | |
|  | **Cis-element** | **Sequence** | **Position(strand)** |  | **Cis-element** | **Sequence** | **Position(strand)** |
| **1** | ABRE | CACGTG | 545(+) | **1** | ABRELATERD1 | ACGTG | 545(-), 546(+) |
| **2** | ARE | TGGTTT | 1001(+) | **2** | ACGTATERD1 | ACGT | 440(+),440(-), 546(+),546(-) |
| **3** | Box-W1 | TTGACC | 359(-) | **3** | ASF1MOTIFCAMV | TGACG | 1175(+) |
| **4** | CGTCA motif  TGACG-motif | CGTCA  TGACG | 1175(-)  1175(+) | **4** | CBFHV | RYCGAC | 220(-) |
|  |  |  |  | **5** | DPBFCOREDCDC3 | ACACNNG | 934(-) |
| **5** | MBS | TAACTG | 822(+), 1243(+), 1448(-) | **6** | ELRECOREPCRP1 | TTGACC | 359(-) |
| **6** | TCA-element | CAGAAAAGGA | 254(+), 765(+), 746(-) | **7** | ERELEE4 | AWTTCAA | 1258(+), 1339(-) |
|  | | | | **8** | LTRECOREATCOR15 | CCGAC | 1294(+) |
|  |  |  |  | **9** | MYBCORE | CNGTTR | 306(-), 1316(+), 1448(+), 822(-), 1243(-) |
|  |  |  |  | **10** | MYB2AT | TAACTG | 822(+), 1243(+),1448(-) |
|  |  |  |  | **11** | MYB1AT | WAACCA | 1001(-) |
|  |  |  |  | **12** | MYB2CONSENSUSAT | YAACKG | 822(+), 1243(+),1448(-) |

| **Supplementary Table S10.4a Vang09g01780** | | | | | | | |
| --- | --- | --- | --- | --- | --- | --- | --- |
| **PlantCare** | | | | **PLACE** | | | |
|  | **Cis-element** | **Sequence** | **Position(strand)** |  | **Cis-element** | **Sequence** | **Position(strand)** |
| **1** | ARE | TGGTTT | 1434(-), 1476(-) | **1** | ACGTATERD1 | ACGT | 757(-), 757(+) |
| **2** | BoxW1 | TTGACC | 750(-), 1361(-) | **2** | ASF1MOTIFCAMV | TGACG | 955(+) |
| **3** | CGCTA motif  TGACG-motif | CGCTA  TGACG | 955(-)  955(+) | **3** | CURECORECR | GTAC | 450(-), 459(+), 759(-), 759(+) |
|  |  |  |  | **4** | ERELEE4 | AWTTCAA | 1024(-) |
| **4** | ERE | ATTTCAAA | 1024(-) | **5** | ELRECOREPCRP1 | TTGACC | 750(-), 1361(-) |
| **5** | HSE | AGAAAATTCG | 1445(-) | **6** | LTRECOREATCOR15 | CCGAC | 1327(+) |
| **6** | MBS | TAACTG | 362(-) | **7** | MYBCORE | CNGTTR | 157(+), 362(+), 1279(+), 1286(-) |
| **7** | TCA-element | CCATCTTTTT  CAGAAAAGGA | 374(-)  1438(+) | **8** | MYB2CONSENSUSAT | YAACKG | 362(-) |
|  |  |  |  |  | MYB2AT | TAACTG | 362(-) |
|  | | | | **9** | MYB1AT | WAACCA | 305(-), 344(+), 1434(+), 1476(+) |
|  |  |  |  | **10** | WBOXNTERF3 | TGACY | 382(-), 504(+), 750(-), 801(-), 910(-), 1361(-), 1371(-) |

| **Supplementary Table S10.5a Vang11g11810** | | | | | | | |
| --- | --- | --- | --- | --- | --- | --- | --- |
| **PlantCare** | | | | **PLACE** | | | |
|  | **Cis-element** | **Sequence** | **Position(strand)** |  | **Cis-element** | **Sequence** | **Position(strand)** |
| **1** | ARE | TGGTTT | 1446(-) | **1** | ACGTATERD1 | ACGT | 643(-), 643(+), 1096(-), 1096(+) |
| **2** | BoxW1 | TTGACC | 28(+) | **2** | CURECORECR | GTAC | 212(-), 212(+), 479(-), 479(+), 1017(-), 1017(+) |
| **3** | HSE | AAAAAATTTC  AGAAAATTCG | 17(-), 652(+), 1069(+)  402(-) | **3** | DPBFCOREDCDC3 | ACACNNG | 214(+), 510(+) |
|  |  |  |  | **4** | ELRECOREPCRP1 | TTGACC | 28(+) |
| **4** | LTR | CAACTG | 1418(+) | **5** | MYBCORE | CNGTTR | 977(-), 1396(-), 1453(-) |
| **5** | MBS | TAACTG | 977(+) | **6** | MYB1AT | WAACCA | 572(+), 1446(+) |
| **6** | TCA-element | GAGAAGAATA | 961(-) | **7** | MYB2CONSENSUSAT | YAACKG | 977(+) |
|  | | | | **8** | WBOXNTCHN48 | CTGACY | 285(+), 1344(-) |
|  |  |  |  | **9** | WBOXNTERF3 | TGACY | 29(+), 85(-), 286(+), 396(-), 1087(-), 1334(-), 1344(-) |

| **Supplementary Table S10.6a Vang0027ss00340** | | | | | | | |
| --- | --- | --- | --- | --- | --- | --- | --- |
| **PlantCare** | | | | **PLACE** | | | |
|  | **Cis-element** | **Sequence** | **Position(strand)** |  | **Cis-element** | **Sequence** | **Position(strand)** |
| **1** | ARE | TGGTTT | 624(+) | **1** | ACGTABREMOTIFA2OSEM | ACGTGKC | 1040(-) |
| **2** | Box-W1 | TTGACC | 137(-) | **2** | DRE2COREZMRAB17 | ACCGAC | 949(+) |
| **3** | CGCTA-motif  TGACG-motif | CGTCA  TGACG | 33(-), 964(-), 967(+)  33(+),967(-), 964(+) | **3** | ACGTATERD1 | ACGT | 40(-), 40(+), 289(-), 289(+), 966(-), 966(+), 1043(-), 1043(+), 1107(-), 1107(+) |
|  |  |  |  | **4** | ABRELATERD1 | ACGTG | 40(+), 1042(-), 1107(+) |
| **4** | ERE | ATTTCAAA | 912(-), 1378(+) | **5** | CBFHV | RYCGAC | 155(+), 817(-),817(+), 949(+) |
| **5** | TCA-element | GAGAAGAATA | 1208(-), 1480(-) | **6** | ASF1MOTIFCAMV | TGACG | 33(+), 964(+), 967(-) |
|  | | | | **7** | CURECORECR | GTAC | 700(-), 700(+), 935(-), 935(+) |
|  |  |  |  | **9** | LTRECOREATCOR15  DRECRTCOREAT | CCGAC  RCCGAC | 950(-)  949(+) |
|  |  |  |  | **10** | LTREATLTI78 | ACCGACA | 949(+) |
|  |  |  |  | **11** | ERELEE4 | AWTTCAA | 912(-), 919(-) |
|  |  |  |  | **12** | ELRECOREPCRP1 | TTGACC | 137(-) |
|  |  |  |  | **13** | MYB1AT | WAACCA | 364(-), 624(-), 629(-) |
|  |  |  |  | **14** | PREATPRODH | ACTCAT | 429(+) |
|  |  |  |  | **15** | WBOXNTERF3 | TGACY | 110(+), 137(-), 581(-), 908(+), 1403(+), 1492(+) |

| **Supplementary Table S10.7a Vang0942s00010** | | | | | | | |
| --- | --- | --- | --- | --- | --- | --- | --- |
| **PlantCare** | | | | **PLACE** | | | |
|  | **Cis-element** | **Sequence** | **Position(strand)** |  | **Cis-element** | **Sequence** | **Position(strand)** |
| **1** | ABRE | TACGTG | 177(-) | **1** | ACGTATERD1 | ACGT | 178(-), 178(+) |
| **2** | HSE | AAAAAATTTC | 840(+) | **2** | ABRELATERD1 | ACGTG | 177(-) |
| **3** | LTR | CCGAAA | 509(+) | **3** | CBFHV | RYCGAC | 424(-) |
|  |  |  |  | **4** | BOXLCOREDCPAL | ACCWWCC | 287(-) |
|  | | | | **5** | CURECORECR | GTAC | 92(-), 92(+), 995(-), 995(+) |
|  |  |  |  | **6** | DPBFCOREDCDC3 | ACACNNG | 649(+), 1168(+) |
|  |  |  |  | **7** | LTRE1HVBLT49 | CCGAAA | 509(+) |
|  |  |  |  | **8** | MYBCORE | CNGTTR | 18(-), 138(+), 688(-), 1378(+) |
|  |  |  |  | **9** | MYB1AT | WAACCA | 951(+), 1152(-), 1362(+) |
|  |  |  |  | **10** | MYCATRD1  MYCATRD22 | CATGTG  CACATG | 520(+)  520(-) |
|  |  |  |  | **11** | WBOXNTCHN48 | CTGACY | 1024(-) |
|  |  |  |  | **12** | WBOXNTERF3 | TGACY | 380(-), 923(+), 1024(-) |

| **Supplementary Table S10.8a Vang08g06450** | | | | | | | |
| --- | --- | --- | --- | --- | --- | --- | --- |
| **PlantCare** | | | | **PLACE** | | | |
|  | **Cis-element** | **Sequence** | **Position(strand)** |  | **Cis-element** | **Sequence** | **Position(strand)** |
| **1** | ABRE | TACGTG  CACGTG | 386(-)  1187(-) | **1** | ACGTATERD1 | ACGT | 257(-), 257(+), 387(-), 387(+), 1188(-), 1188(+), |
|  |  |  |  | **2** | ABRELATERD1 | ACGTG | 387(-), 1188(+) |
| **2** | ARE | TGGTTT | 270(-), 1468(+), 278(-) | **3** | CURECORECR | GTAC | 1214(-), 1214(+) |
| **3** | Box-W1 | TTGACC | 1319(+) | **4** | DRE2COREZMRAB17 | ACCGAC | 56(-) |
| **4** | ELI-Box | AAACCAAT | 214(-), 278(+) | **5** | DPBFCOREDCDC3 | ACACNNG | 996(+), 1170(+), 1187(-), 1216(+) |
| **5** | HSE | CNNGAANNTTCNNG | 1399(-) | **6** | ELRECOREPCRP1 | TTGACC | 1319(+) |
| **6** | MBS | CAACTG | 371(+) | **7** | MYBCORE | CNGTTR | 41(+), 371(-), 629(-), 1237(-), 1316(+), |
| **7** | TCA-element | CCATCTTTTT | 1374(+) | **8** | MYB1AT | WAACCA | 217(-), 233(-), 278(+), 654(+), 1241(+), 1468(+) |
| **8** | Wun Box | TCATTACGAA | 825(+) | **9** | MYB2CONSENSUSAT | YAACKG | 371(+), 1237(-) |
|  | | | | **10** | MYCATERD1  MYCATRD22 | CATGTG  CACATG | 997(-), 1391(-)  997(+), 1391(+) |
|  |  |  |  | **11** | PREATPRODH | ACTCAT | 1064(-) |
|  |  |  |  | **12** | WBOXNTCHN48 | CTGACY | 804(-) |
|  |  |  |  | **13** | WBOXNTERF3 | TGACY | 194(-), 804(-), 959(+), 1320(+), 1395(+) |

| **Supplementary Table S10.9a Vang0333s00130** | | | | | | | |
| --- | --- | --- | --- | --- | --- | --- | --- |
| **PlantCare** | | | | **PLACE** | | | |
|  | **Cis-element** | **Sequence** | **Position(strand)** |  | **Cis-element** | **Sequence** | **Position(strand)** |
| **1** | ABRE | ACGTGGC  CACGTG  TACGGTC  TACGTG  CGCACGTGTC | 87(-)  141(+), 89(+)  308(+)  112(+)  817(-), 130(-) | **1** | ACGTATERD1 | ACGT | 44(-), 44(+), 90(-), 90(+), 142(-), 142(+), 309(-), 309(+), 366(-), 366(+), 818(-), 818(+) |
|  |  |  |  | **2** | ABRELATERD1 | ACGTG | 89(-), 90(+), 141(-), 142(+), 308(-), 309(+), 365(-), 366(+), 817(-) |
|  |  |  |  | **3** | CBFHV | RYCGAC | 106(-) |
|  |  |  |  | **4** | ASF1MOTIFCAMV | TGACG | 56(-) |
|  |  |  |  | **5** | ACGTABREMOTIFA2OSEM  ABREATCONSENSUS  ABREATRD22 | ACGTGKC  YACGTGGC  RYACGTGGYR | 87(-), 139(-)  87(-)  86(-) |
| **2** | ARE | TGGTTT | 166(-), 972(+),526(-) |  |  |  |  |
| **3** | Box-W1 | TTGACC | 115(-), 1276(+), 536(-) |  |  |  |  |
| **4** | CGCTA-motif | CGTCA | 42(-), 56(+) | **6** | DPBFCOREDCDC3 | ACACNNG | 140(-), 141(+), 271(+), 307(+), 936(-) |
| **5** | LTR | CCGAAA | 984(-) | **7** | CURECORECR | GTAC | 956(-), 956(+), 1056(-), 1056(+) |
| **6** | MBS | CGGTCA  TAACTG | 114(+)  911(+) | **8** | DRECRTCOREAT | RCCGAC | 106(-) |
|  |  |  |  | **9** | ELRECOREPCRP1 | TTGACC | 115(-), 536(-), 1276(+) |
|  | | | | **10** | LTRECOREATCOR15 | CCGAC | 106(-) |
|  |  |  |  | **11** | LTRE1HVBLT49 | CCGAAA | 984(-) |
|  |  |  |  | **12** | MYBCORE | CNGTTR | 911(-), 1172(+) |
|  |  |  |  | **13** | MYB1AT | WAACCA | 166(+), 526(+), 970(-) |
|  |  |  |  | **14** | MYB2CONSENSUSAT  MYB2AT | YAACKG  TAACTG | 911(+)  911(+) |
|  |  |  |  | **15** | PREATPRODH | ACTCAT | 1466(-) |
|  |  |  |  | **16** | WBOXNTERF3 | TGACY | 115(-), 147(+), 215(+), 536(-), 836(-), 854(+), 1102(-), 1277(+), 1492(+) |

| **Supplementary Table S10.10a Vang0005s00190** | | | | | | | |
| --- | --- | --- | --- | --- | --- | --- | --- |
| **PlantCare** | | | | **PLACE** | | | |
|  | **Cis-element** | **Sequence** | **Position(strand)** |  | **Cis-element** | **Sequence** | **Position(strand)** |
| **1** | ABRE | TACGTG | 264(-) | **1** | ACGTATERD1 | ACGT | 265(-), 265(+), 1443(-), 1443(+) |
| **2** | ARE | TGGTTT | 713(+) | **2** | ABRELATERD1 | ACGTG | 264(-) |
| **3** | CGTCA motif | CGTCA | 1246(-) | **3** | ASF1MOTIFCAMV | TGACG | 1246(+) |
| **4** | ERE | ATTTCAAA | 377(-) | **4** | CURECORECR | GTAC | 1242(-), 1242(+) |
| **5** | HSE | AAAAAATTTC | 526(+), 1205(+), 814(+) | **5** | DPBFCOREDCDC3 | ACCGAC | 1061(-), 1451(-) |
| **6** | TCA-element | GAGAAGAATA  CCATCTTTTT  TCAGAAGAGG | 509(+), 1219(-)  972(-)  1300(-) | **6** | ERELEE4 | AWTTCAA | 377(-), 1166(+) |
|  |  |  |  | **7** | MYBCORE | CNGTTR | 502(-) |
|  |  |  |  | **8** | MYB1AT | WAACCA | 713(-), 768(+). 1308(-) |
|  | | | | **9** | MYB2CONSENSUSAT | YAACKG | 502(+) |
|  |  |  |  | **10** | MYCATERD1 | CATGTG | 911(+) |
|  |  |  |  | **11** | WBOXNTERF3 | TGACY | 261(-), 1067(+) |

| **Supplementary Table S10.11a Vang04g12730** | | | | | | | |
| --- | --- | --- | --- | --- | --- | --- | --- |
| **PlantCare** | | | | **PLACE** | | | |
|  | **Cis-element** | **Sequence** | **Position(strand)** |  | **Cis-element** | **Sequence** | **Position(strand)** |
| **1** | ABRE | CACGTG  ACGTGGC | 136(+)  1345(-) | **1** | ACGTATERD1 | ACGT | 107(-), 107(+), 137(-), 137(+), 1347(-), 1347(+) |
|  |  |  |  | **2** | ACGTABREMOTIFA2OSEM | ACGTGKC | 1345(-) |
| **2** | ARE | TGGTTT | 230(-), 620(+) | **3** | ABRELATERD1 | ACGTG | 136(-), 137(+), 1347(-) |
| **3** | CGCTA-motif | CGTCA | 263(+), 844(-), 543(-), 1303(-) | **4** | DPBFCOREDCDC3 | ACCGAC | 135(+), 385(+), 614(-), 1097(-) |
| **4** | HSE | AAAAAATTTC  AGAAAATTCG | 656(-), 1388(-)  703(+) | **5** | ASF1MOTIFCAMV | TGACG | 263(-), 543(-), 844(+), 1303(+) |
|  |  |  |  | **6** | CURECORECR | GTAC | 611(-), 611(+) |
| **5** | LTR | CCGAAA | 965(+) | **7** | ERELEE4 | AWTTCAA | 880(-) |
| **6** | TCA-element | TCAGAAGAGG | 1055(-) | **9** | MYB1AT | WAACCA | 230(+), 620(-) |
| **7** | TGACG-motif | TGACG | 263(-), 844(+), 543(+), 1303(+) | **10** | LTRE1HVBLT49 | CCGAAA | 965(+) |
|  | | | | **11** | WBOXNTERF3 | TGACY | 90(+), 147(+), 209(+), 252(+), 494(-) |

| **Supplementary Table S10.12a Vang04g17060** | | | | | | | |
| --- | --- | --- | --- | --- | --- | --- | --- |
| **PlantCare** | | | | **PLACE** | | | |
|  | **Cis-element** | **Sequence** | **Position(strand)** |  | **Cis-element** | **Sequence** | **Position(strand)** |
| **1** | ABRE | CACGTG  TACGTG  ACGTGGC | 1(+),  1018(-)  1016(-) | **1** | ACGTABREMOTIFA2OSEM  ABREATCONSENSUS  ABRECE1HVA22 | ACGTGKC  YACGTGGC  TGCCACCGG | 184(+), 293(+), 1016(-)  1016(-)  1005(+) |
| **2** | ARE | TGGTTT | 322(+), 670(-), 470(+) | **2** | ACGTATERD1 | ACGT | 2(-), 2(+), 184(-), 184(+), 293(-), 293(+), 525(-), 525(+), 887(-), 887(+), 976(-), 976(+), 986(-), 986(+), 1019(-), 1019(+) |
| **3** | CE1 | TGCCACCGG | 1005(+) | **3** | ABRELATERD1 | ACGTG | 1(-), 2(+), 184(+), 293(+), 887(+), 1018(-) |
| **4** | HSE | AAAAAATTTC | 711(-) | **4** | CURECORECR | GTAC | 35(-), 35(+), 112(-), 112(+), 433(-), 433(+), 1028(-), 1028(+), |
| **5** | TCA-element | GAGAAGAATA | 10(+), 351(+) | **5** | CRTDREHVCBF2 | GTCGAC | 988(-), 988(+) |
|  | | | | **6** | CBFHV | RYCGAC | 988(-), 988(+), 1263(+), |
|  |  |  |  | **7** | MYBCORE | CNGTTR | 494(+), 899(+), |
|  |  |  |  | **8** | MYB1AT | WAACCA | 322(-), 470(-), 670(+), 1086(+), 1252(+) |
|  |  |  |  | **9** | MYB2CONSENSUSAT | YAACKG | 899(-) |
|  |  |  |  | **10** | PREATPRODH | ACTCAT | 1152(+) |
|  |  |  |  | **11** | WBOXNTERF3 | TGACY | 39(-), 1422(-) |

| **Supplementary Table S10.13a Vang06g17510** | | | | | | | |
| --- | --- | --- | --- | --- | --- | --- | --- |
| **PlantCare** | | | | **PLACE** | | | |
|  | **Cis-element** | **Sequence** | **Position(strand)** |  | **Cis-element** | **Sequence** | **Position(strand)** |
| **1** | ABRE | GCAACGTGTC | 262(+) | **1** | ACGTATERD1 | ACGT | 1471(-) |
| **2** | Box-W1 | TTGACC | 186(-), 498(+), 251(+), 807(-) | **2** | CURECORECR | GTAC | 1263(-), 1263(+), 1461(-), 1461(+) |
| **3** | EIRE | TTCGACC | 409(+), 522+) | **3** | DPBFCOREDCDC3 | ACCGAC | 342(-), 1483(+) |
| **4** | HSE | AAAAAATTTC | 704(-) | **4** | ELRECOREPCRP1 | AWTTCAA | 186(-), 251(+), 408(+), 807(-) |
| **5**  **6** | MBS  Wun Box | TAACTG  TCATTACGAA | 1063(+), 1098(+)  552(-) | **5** | MYBCORE | CNGTTR | 920(-), 1063(-), 1098(-), 1198(-), |
|  |  |  |  | **6** | MYB1AT | WAACCA | 570(+), 1020(+) |
|  | | | | **7** | MYB2CONSENSUSAT  MYB2AT | YAACKG  TAACGTG | 1063(+), 1098(+), 1190(+)  1063(+), 1098(+) |
|  |  |  |  | **8** | PREATPRODH | ACTCAT | 512(+), 715(-), 818(-) |
|  |  |  |  | **9** | WBOXNTERF3 | TGACY | 14(+), 186(-), 252(+), 278(+), 335(+), 369(+), 442(+), 478(+), 499(+), 807(-) |
|  |  |  |  | **10** | WBOXNTCHN48 | CTGACY | 369(+), 441(+) |

| **Supplementary Table S10.14a Vang08g01000** | | | | | | | |
| --- | --- | --- | --- | --- | --- | --- | --- |
| **PlantCare** | | | | **PLACE** | | | |
|  | **Cis-element** | **Sequence** | **Position(strand)** |  | **Cis-element** | **Sequence** | **Position(strand)** |
| **1** | ARE | TGGTTT | 1013(+), 1408(-), 1173(-), 1466(-) | **1** | BOXLCOREDCPAL | ACCWWCC | 1189(+) |
| **2** | Box-W1 | TTGACC | 1087(-) | **2** | CURECORECR | ACCGAC | 885(-), 885(+), 1078(-), 1078(+) |
| **3** | ERE | ATTTCAAA | 528(-) | **3** | ERELEE4 | AWTTCAA | 529(-), 1389(-) |
| **4** | MBS | TAACTG | 547(-) | **4** | ELRECOREPCRP1 | TTGACC | 1087(-) |
| **5** | TCA-element | CCATCTTTTT | 1215(-), 1305(+) | **5** | MYBCORE | CNGTTR | 240(-), 547(-), 914(-), 992(-) |
|  | | | | **6** | MYB1AT | WAACCA | 1014(-), 1174(-), 1467(-) |
|  |  |  |  | **7** | MYB2CONSENSUSAT  MYB2AT | YAACKG  TAACGTG | 547(-)  547(-) |
|  |  |  |  | **8** | WBOXNTERF3 | TGACY | 87(-), 286(-), 560(+), 850(-), 948(-), 1087(-), 1434(+) |

| **Supplementary Table S10.15a Vang10g06010** | | | | | | | |
| --- | --- | --- | --- | --- | --- | --- | --- |
| **PlantCare** | | | | **PLACE** | | | |
|  | **Cis-element** | **Sequence** | **Position(strand)** |  | **Cis-element** | **Sequence** | **Position(strand)** |
| **1** | ARE | TGGTTT | 255(+), 1273(-), 1172(-) | **1** | ACGTATERD1 | ACGT | 758(-), 758(+), 1268(-), 1268(+),1297(-), 1297(+) |
| **2** | CGCTA-motif  TGACG-motif | CGTCA  TGACG | 1269(+)  1269(-) | **2** | ABRELATERD1 | ACGTG | 1267(-) |
|  |  |  |  | **3** | ASF1MOTIFCAMV | TGACG | 1269(-) |
| **3** | HSE | AAAAAATTTC | 62(+), 565(+) | **4** | DPBFCOREDCDC3 | ACCGAC | 1031(+) |
| **4** | MBS | CAACTG  CGGTCA | 802(+), 1366(+)  1045(+) | **5** | MYBCORE | CNGTTR | 511(-), 802(-), 1366(-) |
|  |  |  |  | **6** | MYB1AT | WAACCA | 255(-), 1009(+), 1026(+), 1172(+), 1273(+) |
| **5** | TCA-element | CAGAAAAGGA | 1372(+) | **7** | MYB2CONSENSUSAT | YAACKG | 802(+), 1366(+) |
|  | | | | **8** | MYCATERD1  MYCATRD22 | CATGTG  CACATG | 659(-)  659(+) |
|  |  |  |  | **9** | LTRECOREATCOR15 | CCGAC | 874(-) |
|  |  |  |  | **10** | PREATPRODH | ACTCAT | 386(+), 455(+) |
|  |  |  |  | **11** | WBOXNTERF3 | TGACY | 1078(+), 1118(-), 1305(-), 1421(-) |
|  |  |  |  | **12** | WBOXNTCHN48 | CTGACY | 1077(+), 1421(-) |

| **Supplementary Table S10.16a Vang07g06810** | | | | | | | |
| --- | --- | --- | --- | --- | --- | --- | --- |
| **PlantCare** | | | | **PLACE** | | | |
|  | **Cis-element** | **Sequence** | **Position(strand)** |  | **Cis-element** | **Sequence** | **Position(strand)** |
| **1** | ARE | TGGTTT | 42(+), 1205(+), 245(+) | **1** | ACGTABREMOTIFA2OSEM | ACGTGKC | 16(+) |
| **2** | CGCTA-motif  TGACG-motif | CGTCA  TGACG | 14(-), 1241(-)  14(+), 1241(+) | **2** | ACGTATERD1 | ACGT | 16(-), 16(+), 1243(-), 1243(+) |
|  |  |  |  | **3** | ABRELATERD1 | ACGTG | 16(+), 1243(+) |
| **3** | HSE | AAAAAATTTC | 294(+), 839(+) | **4** | ASF1MOTIFCAMV | TGACG | 14(+), 1241(+) |
| **4** | LTR | CCGAAA | 1167(+) | **5** | BOXLCOREDCPAL | ACCWWCC | 55(-) |
| **5** | TCA-element | GAGAAGAATA | 536(+) | **6** | CURECORECR | ACCGAC | 1247(-), 1247(+) |
|  | | | | **7** | MYB1AT | WAACCA | 42(-), 245(-), 779(-), 1295(-) |
|  |  |  |  | **8** | MYCATERD1 | CATGTG | 120(-), 1100(-) |
|  |  |  |  | **9** | LTRE1HVBLT49 | CCGAAA | 1167(+) |
|  |  |  |  | **10** | WBOXNTCHN48 | CTGACY | 1258(-) |
|  |  |  |  | **11** | WBOXNTERF3 | TGACY | 1258(-), 1316(-), 1326(+) |

| **Supplementary Table S10.17a Vang0228s00230** | | | | | | | |
| --- | --- | --- | --- | --- | --- | --- | --- |
| **PlantCare** | | | | **PLACE** | | | |
|  | **Cis-element** | **Sequence** | **Position(strand)** |  | **Cis-element** | **Sequence** | **Position(strand)** |
| **1** | ARE | TGGTTT | 199(-), 820(-) | **1** | ACGTATERD1 | ACGT | 492(-), 492(+), 683(-), 683(+), 1064(-), 1064(+), 1387(-), 1387(+) |
| **2** | CGTCA motif  TGACG-motif | CGTCA  TGACG | 512(-)  512(+) | **2** | ASF1MOTIFCAMV | TGACG | 512(+) |
|  |  |  |  | **3** | BOXLCOREDCPAL | ACCWWCC | 1281(+), 1402(+) |
| **3** | ERE | ATTTCAAA | 408(+), 1220(-) | **4** | CURECORECR | ACCGAC | 1400(-), 1400(+) |
| **4** | HSE | AAAAAATTTC  AGAAAATTCG | 379(-), 1105(+)  380(-), 1134(+) | **5** | DPBFCOREDCDC3 | ACCGAC | 777(-), 914(-), 1367(-) |
|  |  |  |  | **6** | ERELEE4 | AWTTCAA | 408(+), 1220(-) |
| **5** | LTR | CCGAAA | 1155(+) | **7** | MYBCORE | CNGTTR | 49(-), 581(-) |
| **6** | MBS | TAACTG | 581(+) | **8** | MYB1AT | WAACCA | 199(+), 820(+), 852(-) |
| **7** | TCA-element | AACGAC | 1249(+), 1256(+) | **9** | MYB2CONSENSUSAT  MYB2AT | YAACKG  TAACGTG | 581(+)  581(+) |
|  |  |  |  |  |  |  |  |
|  |  |  |  | **10** | LTRE1HVBLT49 | CCGAA | 1155(+) |
|  |  |  |  | **11** | WBOXNTERF3 | TGACY | 270(+), 401(+), 437(+), 725(+), 1264(-) |

| **Supplementary Table S10.1b Vradi05g21980** | | | | | | | |
| --- | --- | --- | --- | --- | --- | --- | --- |
| **PlantCare** | | | | **PLACE** | | | |
|  | **Cis-element** | **Sequence** | **Position(strand)** |  | **Cis-element** | **Sequence** | **Position(strand)** |
| 1 | ARE | TGGTTT | 1146(+) | 1 | BOXLCOREDCPAL | ACCWWCC | 434(+) |
| 2 | ERE | ATTTCAAA | 1263(-) | 2 | ERELEE4 | CCGAC | 1263(-) |
| 3 | HSE | AAAAAATTTC | 535(-), 1266(-) | 3 | MYBCORE | CNGTTR | 471(-), 614(-), ,709(-), 768(-), 809(-), 857(-), 860(+) |
| 4 | MBS | CAACTG  TAACTG | 709(+)  809(+) | 4 | MYB1AT | WAACCA | 1146(-) |
|  |  |  |  | 5 | MYB2CONSENSUSAT  MYB2AT | YAACKG  TAACTG | 709(+), 809(+), 860(-)  809(+) |
| 5 | TCA-element | CAGAAAAGGA  CCATCTTTTT | 130(+)  597(+) |  |  |  |  |
|  |  |  |  | 6 | LTRECOREATCOR15 | CCGAC | 820(-) |
|  | | | | 7 | PREATPRODH | ACTCAT | 945(+) |

| **Supplementary Table S10.2b Vradi0158s00480** | | | | | | | |
| --- | --- | --- | --- | --- | --- | --- | --- |
| **PlantCare** | | | | **PLACE** | | | |
|  | **Cis-element** | **Sequence** | **Position(strand)** |  | **Cis-element** | **Sequence** | **Position(strand)** |
| 1 | ABRE | TACGTG | 1385(-) | 1 | ACGTATERD1 | ACGT | 1044(-), 1044(+), 1387(-), 1387(+), |
| 2 | ARE | TGGTTT | 1173(+) | 2 | ABRELATERD1 | ACGTG | 1386(-) |
| 3 | Box-W1 | TTGACC | 1236(-), 1253(+) | 3 | ASF1MOTIFCAMV | TGACG | 1045(-) |
| 4 | MBS | TAACTG  CAACTG | 707(-)  1231(+) | 4 | BOXLCOREDCPAL | ACCWWCC | 1339(+) |
|  |  |  |  | 5  6 | CURECORECR  DPBFCOREDCDC3 | GTAC  ACACNNG | 496(-), 496(+), 1061(-), 1061(+)  132(+) |
| 5 | TCA-element | CAGAAAAGGA  CCATCTTTTT | 130(+)  597(+) |  |  |  |  |
|  |  |  |  | 7 | ELRECOREPCRP1 | TTGACC | 1236(-), 1253(+) |
| 6 | TGACG-motif | TGACG | 1045(-) | 8 | MYBCORE | CNGTTR | 701(-), 708(+), 1163(-), 1231(-) |
|  | | | | 9 | MYB1AT | WAACCA | 310(-), 479(+), 1174(+) |
|  |  |  |  | 10 | MYB2CONSENSUSAT  MYB2AT | YAACKG  TAACTG | 708(-), 1163(+), 1231(+)  708(-) |
|  |  |  |  | 11 | WBOXNTERF3 | TGACY | 1236(-), 1254(+), 1329(-) |

| **Supplementary Table S10.3b Vradi06g06770** | | | | | | | |
| --- | --- | --- | --- | --- | --- | --- | --- |
| **PlantCare** | | | | **PLACE** | | | |
|  | **Cis-element** | **Sequence** | **Position(strand)** |  | **Cis-element** | **Sequence** | **Position(strand)** |
| 1 | ARE | TGGTTT | 1386(-) | 1 | ACGTATERD1 | ACGT | 1407(-), 1407(+) |
| 2 | Box-W1 | TTGACC | 1326(-) | 2 | ASF1MOTIFCAMV | TGACG | 364(-) |
| 3 | CGCTA-motif  TGACG-motif | CGTCA  TGACG | 364(+)  364(-) | 3 | BOXLCOREDCPAL | ACCWWCC | 1358(+) |
|  |  |  |  | 4 | CURECORECR | GTAC | 918(-), 918(+), 1409(-), 1409(+) |
| **4** | ERE | ATTTCAAA | 1273(+) | 5 | DPBFCOREDCDC3 | ACACNNG | 905(+) |
| 5 | HSE | AAAAAATTTC | 1268(+) | 6 | ERELEE4 | AWTTCAA | 1274(+) |
| 6 | MBS | CGGTC | 1325(+) | 7 | ELRECOREPCRP1 | TTGACC | 1327(-) |
| 7 | TCA-element | CCATCTTTTT | 1003(-) | 8 | MYB1AT | WAACCA | 1387(+) |
|  |  | GAGAAGAATA | 1477(-) | 9 | WBOXNTERF3 | TGACY | 1232(-), 1253(+), 1327(-) |

| **Supplementary Table S10.4b Vradi03g09710** | | | | | | | |
| --- | --- | --- | --- | --- | --- | --- | --- |
| **PlantCare** | | | | **PLACE** | | | |
|  | **Cis-element** | **Sequence** | **Position(strand)** |  | **Cis-element** | **Sequence** | **Position(strand)** |
| 1 | ABRE | CACGTG | 789(-) | 1 | ACGTATERD1 | ACGT | 790(-), 790(+) |
| 2 | ARE | TGGTTT | 303(-), 1476(-), 1038(+), 934(-), 1434(-) | 2 | ABRELATERD1 | ACGTG | 789(-), 790(+) |
| 3 | Box-W1 | TTGACC | 203(+),1361(-) | 3 | DPBFCOREDCDC3 | ACACNNG | 123(+), 125(+), 224(+) |
| 4 | EIRE | TTCGACC | 1456(-) | 4 | ERELEE4 | AWTTCAA | 1018(-) |
| 5 | ERE | ATTTCAAA | 1018(-) | 5 | ELRECOREPCRP1 | TTGACC | 203(+), 1361(-) |
| 6 | HSE | AAAAAATTTC | 885(-) | 6 | LTRECOREATCOR15 | CCGAC | 1327(+) |
| 7 | MBS | TAACTG | 1078(-) | 8 | MYBCORE | CNGTTR | 1078(+), 1279(+), 1286(-) |
| 8 | TCA-element | CAGAAAAGGA | 624(+, 1438(+) | 9 | MYB1AT | WAACCA | 303(+), 934(+), 1038(-), 1434(+), 1476(-) |
|  | | | | 10 | MYB2CONSENSUSAT  MYB2AT | YAACKG  TAACTG | 1078(-)  1078(-) |
|  |  |  |  | 11 | MYCATERD1  MYCATERD22 | CATGTG  CACATG | 225(-)  225(+) |
|  |  |  |  | 12 | WBOXNTERF3 | TGACY | 204(+), 533(+), 811(+), 1361(-), 1371(-) |

| **Supplementary Table S10.5b Vradi07g21330** | | | | | | | |
| --- | --- | --- | --- | --- | --- | --- | --- |
| **PlantCare** | | | | **PLACE** | | | |
|  | **Cis-element** | **Sequence** | **Position(strand)** |  | **Cis-element** | **Sequence** | **Position(strand)** |
| 1 | ABRE | TACGTG | 895(+) | 1 | ACGTATERD1 | ACGT | 896(-), 996(+), 1078(-), 1078(+), 1094(-), 1094(+), 1422(-), 1422(+) |
| 2 | ARE | TGGTTT | 534(+), 1446(+) | 2 | ABRELATERD1 | ACGTG | 896(+) |
| 3 | Box-W1 | TTGACC | 32(+) | 3 | ASF1MOTIFCAMV | TGACG | 502(-) |
| 4 | CGCTA-motif  TGACG-motif | CGTCA  TGACG | 502(+)  502(-) | 4 | CURECORECR | GTAC | 489(-), 489(+) |
|  |  |  |  | 5 | DPBFCOREDCDC3 | ACACNNG | 221(+), 484(-), 901(-) |
| 5 | LTR | CCGAAA | 1417(+) | 5 | ELRECOREPCRP1 | TTGACC | 32(+) |
|  | | | | 6 | LTRE1HVBLT49 | CCGAAA | 1417(+) |
|  |  |  |  | 7 | MYBCORE | CNGTTR | 1176(+), 1395(-), 1453(-) |
|  |  |  |  | 8 | MYB1AT | WAACCA | 534(-), 1446(+) |
|  |  |  |  | 9 | MYCATERD1  MYCATERD22 | CATGTG  CACATG | 484(+)  484(-) |
|  |  |  |  | 10 | WBOXNTCHN48 | CTGACY | 1343(-) |
|  |  |  |  | 11 | WBOXNTERF3 | TGACY | 33(+), 89(-), 294(-), 1078(-), 1333(-) |

| **Supplementary Table S10.6b Vradi05g10960** | | | | | | | |
| --- | --- | --- | --- | --- | --- | --- | --- |
| **PlantCare** | | | | **PLACE** | | | |
|  | **Cis-element** | **Sequence** | **Position(strand)** |  | **Cis-element** | **Sequence** | **Position(strand)** |
| 1 | ABRE | TACGGTC | 924(+) | 1 | ACGTATERD1 | ACGT | 143(-), 143(+), 493(-), 493(+), 618(-), 618(+), 912(-), 912(+), |
| 2 | ARE | TGGTTT | 1459(-) | 2 | ABRELATERD1 | ACGTG | 142(-) |
| 3 | Box-W1 | TTGACC | 784(-), 927(+), 952(+) | 3 | ASF1MOTIFCAMV | TGACG | 491(+), 616(+), 913(-) |
| 4 | CGCTA-motif  TGACG-motif | CGTCA  TGACG | 491(-), 913(+), 616(-)  491(+), 913(-), 616(+) | 4 | BOXLCOREDCPAL | ACCWWCC | 1472(+) |
|  |  |  |  | 5 | CBFHV | RYCGAC | 1468(+) |
| 5 | MBS | CAACTG | 783(+), 926(+) | 6 | CURECORECR | GTAC | 1179(-), 1179(+), 1295(-), 1295(+) |
| 6 | TCA element | CCATCTTTTT | 829(+) | 7 | ERELEE4 | AWTTCAA | 499(+) |
|  | | | | 8 | ELRECOREPCRP1 | TTGACC | 784(-), 927(+), 952(+) |
|  |  |  |  | 9 | MYBCORE | CNGTTR | 787(-), 1259(+) |
|  |  |  |  | 10 | MYB1AT | WAACCA | 545(+), 893(+), 1459(-) |
|  |  |  |  | 11 | MYCATERD1  MYCATERD22 | CATGTG  CACATG | 752(-)  752(+) |
|  |  |  |  | 12 | WBOXNTERF3 | TGACY | 1(-), 256(+), 761(+), 784(-),907(-), 927(-), 953(+) |

| **Supplementary Table S10.7b Vradi06g13520** | | | | | | | |
| --- | --- | --- | --- | --- | --- | --- | --- |
| **PlantCare** | | | | **PLACE** | | | |
|  | **Cis-element** | **Sequence** | **Position(strand)** |  | **Cis-element** | **Sequence** | **Position(strand)** |
| 1 | ARE | TGGTTT | 1092(-) | 1 | ACGTATERD1 | ACGT | 118(-), 118(+), 1062(-), 1062(+) |
| 2 | HSE | AAAAAATTTC  AGAAAATTCG | 153(-), 809(+)  1100(-) | 2 | ABRELATERD1 | ACGTG | 1061(-) |
|  |  |  |  | 3 | CBFHV | RYCGAC | 1134(+) |
| 3 | MBS | TAACTG  CAACTG | 77(+)  1489(-) | 4 | CURECORECR | GTAC | 1005(-), 1005(+), 1050(-), 1050(+), 1151(-), 1151(+) |
|  |  |  |  | 5 | DPBFCOREDCDC3 | ACACNNG | 1235(+), 1252(+) |
| 4 | LTR | CCGAAA | 1112(+) | 6 | MYBCORE | CNGTTR | 77(-), 1489(+) |
|  | | | | 7 | MYB1AT | WAACCA | 81(-), 421(+), 1092(+) |
|  |  |  |  | 8 | MYB2AT | TAACTG | 77(+) |
|  |  |  |  | 9 | MYCATERD1  MYCATERD22 | CATGTG  CACATG | 1253(-)  1253(+) |
|  |  |  |  | 10 | LTRE1HVBLT49 | CCGAAA | 1112(+) |
|  |  |  |  | 11 | PREATPRODH | ACTCAT | 1392(+) |
|  |  |  |  | 12 | WBOXNTCHN48 | CTGACY | 763(+) |
|  |  |  |  | 13 | WBOXNTERF3 | TGACY | 447(-),764(+), 786(+), 821(-), 1179(+) |

| **Supplementary Table S10.8b Vradi07g15410** | | | | | | | |
| --- | --- | --- | --- | --- | --- | --- | --- |
| **PlantCare** | | | | **PLACE** | | | |
|  | **Cis-element** | **Sequence** | **Position(strand)** |  | **Cis-element** | **Sequence** | **Position(strand)** |
| 1 | ARE | TGGTTT | 676(-) | 1 | ACGTATERD1 | ACGT | 662(-), 662(+), 783(-), 783(+), 1292(-), 1292(+), 1318(-), 1318(+) |
| 2 | Box-W1 | TTGACC | 933(+), 1278(+) | 2 | ASF1MOTIFCAMV | TGACG | 784(-) |
| 3 | CGCTA motif  TGACG-motif | CGCTA  TGACG | 784(+)  784(-) | 3 | BOXLCOREDCPAL | ACCWWCC | 553(+) |
| 4 | HSE | AAAAAATTTC  AGAAAATTCG | 297(+)  956(-) | 4 | CBFHV  CRTDREHVCBF2 | RYCGAC  GTCGAC | 1288(-), 1288(+)  1288(-), 1288(+) |
| 5 | MBS | TAACTG  CAACTG | 1103(+)  1243(+) | 5 | CURECORECR | GTAC | 278(-), 278(+), 1320(-), 1320(+) |
| 6 | TCA-element | TCAGAAGAGG | 949(-) | 6 | DPBFCOREDCDC3 | ACACNNG | 161(-), 1106(-), 1221(+), 1393(-),1428(-) |
|  |  |  |  | 7 | ELRECOREPCRP1 | TTGACC | 933(+), 1268(+) |
|  | | | | 8 | MYBCORE | CNGTTR | 1103(-), 1280(+) |
|  |  |  |  | 9 | MYB1AT | WAACCA | 677(+) |
|  |  |  |  | 10 | MYB2CONSENSUSAT  MYB2AT | YAACKG  TAACTG | 1103(+), 1280(-)  1103(+) |
|  |  |  |  | 11 | MYCATERD1  MYCATERD22 | CATGTG  CACATG | 1302(+), 1393(+)  1302(-), 1393(-) |
|  |  |  |  | 12 | PREATPRODH | ACTCAT | 1033(+) |
|  |  |  |  | 13 | WBOXNTERF3 | CTGACY | 9(-), 633(+), 934(+), 1153(+), 1245(-), 1269(+), 1306(+), 1380(-) |

| **Supplementary Table S10.9b Vradi0146s00260** | | | | | | | |
| --- | --- | --- | --- | --- | --- | --- | --- |
| **PlantCare** | | | | **PLACE** | | | |
|  | **Cis-element** | **Sequence** | **Position(strand)** |  | **Cis-element** | **Sequence** | **Position(strand)** |
| **1** | ARE | TGGTTT | 506(-) | **1** | ACGTATERD1 | ACGT | 243(-), 244(+) |
| **2** | CGCTA motif  TGACG motif | CGCTA  TGACG | 540(+), 820(-), 1479(-)  540(+), 820(-), 1479(-) | **2** | ABRELATERD1 | ACGTG | 243(-) |
|  |  |  |  | **3** | ASF1MOTIFCAMV | TGACG | 540(-), 820(+), 1479(+) |
| **3** | HSE | AAAAAATTTC  AGAAAATTCG | 409(+)  942(-) | **4** | CURECORECR | GTAC | 67(+), 67(-), 777(+), 777(-) |
|  |  |  |  | **5** | DPBFCOREDCDC3 | ACACNNG | 853(-), 1370(+) |
| **4** | LTR | CCGAAA | 1139(+) | **6** | ERELEE4 | AWTTCAA | 632(+), 946(+), 1095(-) |
| **5** | TCA-element | TCAGAAGAGG | 1229(-) | **7** | LTRE1HVBLT49 | CCGAAA | 1139(+) |
|  |  |  |  | **8** | MYB1AT | WAACCA | 506(+) |

| **Supplementary Table S10.10b Vradi06g02270** | | | | | | | |
| --- | --- | --- | --- | --- | --- | --- | --- |
| **PlantCare** | | | | **PLACE** | | | |
|  | **Cis-element** | **Sequence** | **Position(strand)** |  | **Cis-element** | **Sequence** | **Position(strand)** |
| 1 | ABRE | CACGTG  TACGTG  CGCACGTGTC | 205(+)  163(+), 482(+)  161(+) | 1 | ACGTABREMOTIFA2OSEM | ACGTGKC | 205(-) |
|  |  |  |  | 2 | ACGTATERD1 | ACGT | 164(-), 164(+), 206(-), 206(+), 288(-), 288(+), 483(-), 483(+), 1012(-), 1012(+), |
|  |  |  |  | 3 | ABRELATERD1 | ACGTG | 164(+), 205(-) , 206(+), 288(+), 483(+) |
| 2 | ARE | TGGTTT | 474(-), 1215(+) | 4 | CURECORECR | GTAC | 162(-), 162(+), 1076(-), 1076(+), 1113(-), 1113(+) |
| 3 | Box-W1 | TTGACC | 178(-), 519(-) | 5 | DPBFCOREDCDC3 | ACACNNG | 204(+), 204(-), 231(+) |
| 4 | ERE | ATTTCAAA | 527(-), 920(-) | 6 | ERELEE4 | AWTTCAA | 527(-), 920(-) |
| 5 | HSE | AAAAAATTTC | 530(-) | 7 | ELRECOREPCRP1 | TTGACC | 178(-), 519(-) |
| 6 | MBS | CGGTC  CAACTG | 177(+)  776(+) | 8 | MYBCORE | CNGTTR | 776(-) |
|  |  |  |  | 9 | MYB1AT | WAACCA | 474(+), 1215(-), |
| 7 | TCA-element | GAGAAGAATA | 539(-) | 10 | MYB2CONSENSUSAT | YAACKG | 776(+) |
|  | | | | 11 | MYCATERD1  MYCATERD22 | CATGTG  CACATG | 1141(-), 1383(-)  1141(+), 1383(+) |
|  |  |  |  | 12 | LTRECOREATCOR15 | CCGAC | 251(-) |
|  |  |  |  | 13 | PREATPRODH | ACTCAT | 1003(+) |
|  |  |  |  | 14 | WBOXNTCHN48 | CTGACY | 779(+) |
|  |  |  |  | 15 | WBOXNTERF3 | CTGACY | 181(-), 265(+), 522(-)720(-), 783(+) |

| **Supplementary Table S10.11b Vradi0048s00470** | | | | | | | |
| --- | --- | --- | --- | --- | --- | --- | --- |
| **PlantCare** | | | | **PLACE** | | | |
|  | **Cis-element** | **Sequence** | **Position(strand)** |  | **Cis-element** | **Sequence** | **Position(strand)** |
| 1 | ABRE | ACGTGGC  CACGTG  TACGGT  TACGTG  CGCACGTGTC | 291(-)  345(+), 393(+)  316(+)  873(-)  343(-) | 1 | ACGTABREMOTIFA2OSEM  ABREATCONSENSUS  ABREATRD22 | ACGTGKC  YACGTGGC  RYACGTGGYR | 291-), 342(-)  291(-)  290(-) |
|  |  |  |  | 2 | ACGTATERD1 | ACGT | 75(-), 75(+), 248)-), 248(+), 294(-), 294(+), 346(-), 346(+), 563(-), 559(+), 868(-), 868(+) |
|  |  |  |  | 3 | ASF1MOTIFCAMV | TGACG | 246(+), 260(-) |
| 2 | ARE | TGGTTT | 370(-), 1029(+), 724(-) | 4 | ABRELATERD1 | ACGTG | 293(-), 294(+), 345(-), 346(+), 564(-), 873(-) |
| 3 | Box-W1 | TTGACC | 319(-) | 5 | CURECORECR | GTAC | 1013(-), 1013(+) |
| 4 | CGCTA-motif  TGACG-motif | CGTCA  TGACG | 246(-), 260(+)  246(+), 260(-) | 6 | CBFHV | RYCGAC | 310(-) |
|  |  |  |  | 7 | DPBFCOREDCDC3 | ACACNNG | 84(-), 344(+), 345(-), 948(+) |
| 5 | LTR | CCGAAA | 1041(-) | 8 | DRECRTCOREAT | RCCGAC | 310(-) |
| 6 | MBS | CGGTC  TAACTG | 318(+)  968(+) | 9 | ELRECOREPCRP1 | TTGACC | 319(-) |
|  |  |  |  | 10 | MYBCORE | CNGTTR | 968(-), 1241(+) |
| 7 | TCA-element | CAGAAAAGGA  CCATCTTTTT | 102(+)  914(-) | 11 | MYB1AT | WAACCA | 370(+), 724(+), 734(-), 1029(-) |
|  |  |  |  | 12 | MYB2CONSENSUSAT | YAACKG | 968(+) |
|  | | | | 13 | MYCATERD1  MYCATERD22 | CATGTG  CACATG | 83(+)  83(-) |
|  |  |  |  | 14 | LTRECOREATCOR15 | CCGAC | 310(-) |
|  |  |  |  | 15 | LTRE1HVBLT49 | CCGAAA | 1041(-) |
|  |  |  |  | 16 | PREATPRODH | ACTCAT | 1466(-) |
|  |  |  |  | 17 | WBOXNTERF3 | CTGACY | 319(-), 391(+), 419(+), 892(-), 910(+), 1170(-), 1310(-) |

| **Supplementary Table S10.12b Vradi0100s00500** | | | | | | | |
| --- | --- | --- | --- | --- | --- | --- | --- |
| **PlantCare** | | | | **PLACE** | | | |
|  | **Cis-element** | **Sequence** | **Position(strand)** |  | **Cis-element** | **Sequence** | **Position(strand)** |
| 1 | ARE | TGGTTT | 272(+), 673(+) | 1 | ACGTATERD1 | ACGT | 1446(-), 1446(+) |
| 2 | CGCTA-motif | CGTCA | 1268(-) | 2 | ASF1MOTIFCAMV | TGACG | 1268(+) |
| 3 | EL3-Box | AAACCAATT | 670(-) | 3 | CURECORECR | GTAC | 1264(-), 1264(+) |
| 4 | HSE | AAAAAATTTC | 524(+), 1226(+) | 4 | CBFHV | RYCGAC | 729(-) |
| 5 | TCA-element | GAGAAGAATA  TCAGAAGAGG | 1240(-)  1322(-) | 5 | DPBFCOREDCDC3 | ACACNNG | 1075(-), 1454(-) |
|  |  |  |  | 6 | ERELEE4 | AWTTCAA | 1188(+) |
|  |  |  |  | 7 | MYB1AT | WAACCA | 272(-), 673(-), 1330(+), |
|  | | | | 8 | MYCATERD1  MYCATERD22 | CATGTG  CACATG | 955(+)  955(-) |
|  |  |  |  | 9 | WBOXNTERF3 | CTGACY | 169(+), , 919(+), 1081(+) |

| **Supplementary Table S10.13b Vradi03g06620** | | | | | | | |
| --- | --- | --- | --- | --- | --- | --- | --- |
| **PlantCare** | | | | **PLACE** | | | |
|  | **Cis-element** | **Sequence** | **Position(strand)** |  | **Cis-element** | **Sequence** | **Position(strand)** |
| 1 | ABRE | CACGTG  TACGTG | 621(+)  393(+) | 1 | ACGTABREMOTIFA2OSEM | ACGTGKC | 799(+), 904(+) |
|  |  |  |  | 2 | ACGTATERD1 | ACGT | 76(-), 76(+), 394(-), 394(+), 590(-), 590(+), 622(-), 622(+), 790(-), 790(+), 799(-), 799(+), 904(-), 904(+), 1127(-), 1127(+), 1384(-), 1384(+), 1473(-), 1473(+), 1483(-), 1483(+) |
| 2 | ARE | TGGTTT | 341(+), 1083(+) 933(+), 1287(+) | 3 | ASF1MOTIFCAMV | TGACG | 680(-) |
| 3 | CGCTA-motif | CGCTA | 680(+) | 4 | ABRELATERD1 | ACGTG | 394(+), 589(-), 621(-), 622(+), 799(+), 904(+), 1384(+) |
|  | TGACG-motif | TGACG | 680(-) | 5 | CURECORECR | GTAC | 654(-), 654(+), 727(-), 727(+), 1046(-), 1046(+) |
|  |  |  |  | 6 | CBFHV  CRTDREHVCBF2 | RYCGAC  GTCGAC | 1485(-), 1485(+)  1485(-), 1485(+) |
| 4 | HSE | AAAAAATTTC | 363(-) | 7 | DPBFCOREDCDC3 | ACACNNG | 618(+), 620(+), 1089(-) |
| 5 | MBS | CAACTG | 135(-) | 8 | DRE1COREZMRAB17 | ACCGAGA | 264(-) |
| 6 | TCA-element | CCATCTTTTT | 597(+) | 9 | MYBCORE | CNGTTR | 0(-), 135(+), 482(+), 1108(+), 1396(+) |
|  | | | | 10 | MYB1AT | WAACCA | 272(+), 341(-), 933(-), 1083(-), 1287(-) |
|  |  |  |  | 11 | MYB2CONSENSUSAT  MYB2AT | YAACKG  TAACTG | 135(-), 1396(-)  135(-) |
|  |  |  |  | 12 | WBOXNTERF3 | TGACY | 658(-) |

| **Supplementary Table S10.14b Vradi08g08840** | | | | | | | |
| --- | --- | --- | --- | --- | --- | --- | --- |
| **PlantCare** | | | | **PLACE** | | | |
|  | **Cis-element** | **Sequence** | **Position(strand)** |  | **Cis-element** | **Sequence** | **Position(strand)** |
| 1 | ARE | TGGTTT | 834(+) | 1 | ACGTATERD1 | ACGT | 426(-), 426(-) |
| 2 | CGCTA-motif  TGACG-motif | CGTCA  TGACG | 1010(+)  1010(-) | 2 | ASF1MOTIFCAMV | TGACG | 1011(-) |
| 3 | MBS | TAACTG | 310(+) | 3 | CURECORECR | GTAC | 237(-), 237(+), 418(-), 418(+), 459(-), 459(+) |
| 4 | TCA-element | GAGAAGAATA  CAGAAAA | 769(-)  1220(-) | 4 | DPBFCOREDCDC3 | ACACNNG | 438(+), 896(-), 1322(-) |
|  | | | | 5 | MYBCORE | CNGTTR | 167(-), 310(-), 385(-), 1129(-), 1450(+) |
|  |  |  |  | 6 | MYB1AT | WAACCA | 835(-) |
|  |  |  |  | 7 | MYB2CONSENSUSAT  MYB2AT | YAACKG  TAACTG | 167(+), 310(+), 1129(+)  310(+) |

| **Supplementary Table S10.15b Vradi07g29640** | | | | | | | |
| --- | --- | --- | --- | --- | --- | --- | --- |
| **PlantCare** | | | | **PLACE** | | | |
|  | **Cis-element** | **Sequence** | **Position(strand)** |  | **Cis-element** | **Sequence** | **Position(strand)** |
| 1 | ARE | TGGTTT | 39(+), 1231(-), 1191(-), 1273(-) | 1 | ACGTATERD1 | ACGT | 1268(-), 1268(+), 1297(-), 1297(+) |
| 2 | HSE | AGAAAATTCG | 316(+) | 2 | ASF1MOTIFCAMV | TGACG | 1269(-) |
| 3 | MBS | CAACTG  TAACTG | 836(+), 1366(+)  1063(+) | 3 | ABRELATERD1 | ACGTG | 1267(-) |
|  |  |  |  | 4 | CURECORECR | GTAC | 449(-), 449(+) |
| 4 | TCA-element | CCATCTTTTT | 331(-) | 5 | DPBFCOREDCDC3 | ACACNNG | 885(-) |
|  | | | | 6 | MYBCORE | CNGTTR | 387(+), 836(-), 1050(-), 1366(-) |
|  |  |  |  | 7 | MYB1AT | WAACCA | 39(-), 1044(+), 1191(+), 1231(+), 1273(+) |
|  |  |  |  | 8 | MYB2CONSENSUSAT | YAACKG | 836(+), 1366(+) |
|  |  |  |  | 9 | MYCATERD1  MYCATERD22 | CATGTG  CACATG | 740(-), 866(+)  740(+), 866(-) |
|  |  |  |  | 10 | PREATPRODH | ACTCAT | 171(+) |
|  |  |  |  | 11 | WBOXNTCHN48 | CTGACY | 907(-), 1095(+), 1424(+) |
|  |  |  |  | 12 | WBOXNTERF3 | TGACY | 907(-), 1064(-) 1096(+), 1136(-), 1305(-), 1425(+) |

| **Supplementary Table S10.16b Vradi11g01720** | | | | | | | |
| --- | --- | --- | --- | --- | --- | --- | --- |
| **PlantCare** | | | | **PLACE** | | | |
|  | **Cis-element** | **Sequence** | **Position(strand)** |  | **Cis-element** | **Sequence** | **Position(strand)** |
| 1 | ARE | TGGTTT | 124(-), 267(-), 219(-) | 1 | ACGTATERD1 | ACGT | 1263(-), 1263(+) |
| 2 | Box-W1 | TTGACC | 1278(-) | 2 | ASF1MOTIFCAMV | TGACG | 1261(+) |
| 3 | CGCTA-motif  TGACG-motif | CGTCA  TGACG | 1261(-)  1261(+) | 3 | ABRELATERD1 | ACGTG | 1263(+) |
|  |  |  |  | 4 | BOXLCOREDCPAL | ACCWWCC | 153(+) |
| 4 | TCA-element | CAGAAAAGGA  GAGAAGAATA | 453(-)  646(-), 597(-) | 5 | CURECORECR | GTAC | 263(-), 263(+), 1267(-), 1267(+) |
|  |  |  |  | 6 | ELRECOREPCRP1 | TTGACC | 1278(-) |
|  | | | | 7 | MYB1AT | WAACCA | 124(+), 219(+), 267(+) |
|  |  |  |  | 8 | PREATPRODH | ACTCAT | 867(+), 1326(-), 1350(+) |
|  |  |  |  | 9 | WBOXNTERF3 | TGACY | 198(+), 638(-), 760(-), 1278(-), 1338(-), 1348(+) |

| **Supplementary Table S10.17b Vradi05g05170** | | | | | | | |
| --- | --- | --- | --- | --- | --- | --- | --- |
| **PlantCare** | | | | **PLACE** | | | |
|  | **Cis-element** | **Sequence** | **Position(strand)** |  | **Cis-element** | **Sequence** | **Position(strand)** |
| 1 | CGCTA-motif  TGACG-motif | CGTCA  TGACG | 1285(+), 1310(+)  1285(-),1310(-) | 1 | ACGTATERD1 | ACGT | 158(-), 158(+0, 1309(-), 1309(+) |
|  |  |  |  | 2 | ASF1MOTIFCAMV | TGACG | 1285(-), 1310(-) |
| 2 | HSE | AAAAAATTTC | 1273(+) | 3 | CURECORECR | GTAC | 311(-), 311(+), 742(-), 742(+), 1202(-), 1202(+) |
| 3 | MBS | CAACTG | 54(+) | 4 | CBFHV | RYCGAC | 64(-) |
| 4 | TCA-element | TCAGAAGAGG | 1298(-) | 5 | DPBFCOREDCDC3 | ACACNNG | 179(+), 822(-) |
| 5 | Wun Box | AAATTTCCT | 1442(+) | 6 | MYBCORE | CNGTTR | 54(-), 120(-) |
|  | | | | 7 | MYB2CONSENSUSAT | YAACKG | 54(+), 120(+) |
|  |  |  |  | 8 | MYCATERD1  MYCATERD22 | CATGTG  CACATG | 180(-), 822(+)  180(+), 822(-) |
|  |  |  |  | 9 | PREATPRODH | ACTCAT | 1139(+) |
|  |  |  |  | 10 | WBOXNTCHN48 | CTGACY | 978(-) |
|  |  |  |  | 11 | WBOXNTERF3 | TGACY | 334(+), 563(-), 978(-), 1212(+) |

| **Supplementary Table S10.18b Vradi05g05160** | | | | | | | |
| --- | --- | --- | --- | --- | --- | --- | --- |
| **PlantCare** | | | | **PLACE** | | | |
|  | **Cis-element** | **Sequence** | **Position(strand)** |  | **Cis-element** | **Sequence** | **Position(strand)** |
| 1 | CGCTA-motif  TGACG-motif | CGTCA  TGACG | 727(-)  727(+) | 1 | ACGTATERD1 | ACGT | 707(-), 707(+), 838(-), 838(+), 903(-), 903(+), 1081(-), 1081(+), 1402(-), 1402(+) |
|  |  |  |  | 2 | ASF1MOTIFCAMV | TGACG | 727(+) |
| 2 | ERE | ATTTCAAA | 622(+), 1235(-) | 3 | BOXLCOREDCPAL | ACCWWCC | 472(+), 1293(+) |
| 3 | HSE | AAAAAATTTC  AGAAAATTCG | 127(+), 577(-)  1149(-) | 4 | CURECORECR | GTAC | 470(-), 470(+), 1109(-), 1109(+), 1415(-), 1415(+) |
|  |  |  |  | 5 | DPBFCOREDCDC3 | ACACNNG | 1382(-) |
| 4 | LTR | CCGAAA | 1170(+) | 6 | ERELEE4 | AWTTCAA | 622(+), 861(+), 1235(-) |
| 5 | MBS | CAACTG | 798(+) | 7 | MYBCORE | CNGTTR | 798(-) |
| 6 | TCA-element | GAGAAGAATA | 1116(+) | 8 | MYB2CONSENSUSAT  MYB2AT | YAACKG  TAACTG | 798(+)  798(+) |
|  | | | | 9 | MYCATERD1  MYCATERD22 | CATGTG  CACATG | 553(-)  553(+) |
|  |  |  |  | 10 | LTRE1HVBLT49 | CCGAAA | 1170(+) |
|  |  |  |  | 11 | WBOXNTERF3 | TGACY | 615(+), 651(+), 945(+), 1278(-) |

| **Supplementary Table S11|** List of stress-responsive *cis*-elements | | | | | | | |
| --- | --- | --- | --- | --- | --- | --- | --- |
| **List of stress-responsive *cis*-elements recognized by New PLACE (SOGO)** | | | | **List of stress-responsive *cis*-elements recognized by PlantCare** | | | |
|  | **Cis-element** | **Sequence** | **Putative function** |  | **Cis-element** | **Sequence** | **Putative function** |
| **1** | **ACGTATERD1** | ACGT | ACGT sequence required for etiolation-induced expression of erd1 (early responsive **to dehydration**) in Arabidopsis; | **1** | **ABRE** | TACGTG/ ACGTGGC /CACGTG/TACGGTC/ TACGTG/ CGCACGTGTC | cis-acting element involved in the abscisic acid responsiveness |
| **2** | **ABRELATERD1** | ACGTG | ACGT sequence required for etiolation-induced expression of erd1 (early responsive to **dehydration**) in Arabidopsis; **ABA responsiveness** | **2** | **ARE** | TGGTTT | cis-acting regulatory element essential for the anaerobic induction |
| **3** | **ACGTABRE**  **-MOTIFA2OSEM** | ACGTGKC | Experimentally determined sequence requirement of ACGT-core of motif A in ABRE (**ABA-responsiveness element**)of the rice gene | **3** | **Box-W1** | TTGACC | fungal elicitor responsive element |
| **4** | **ABREATCONSENSUS** | YACGTGGC | ABA-responsive elements (ABREs) found in the promoter of ABA and/or stress-regulated genes | **4** | **CGCTA-motif** | CGCTA | cis-acting regulatory element involved in the MeJA-responsiveness |
| **5** | **ABREATRD22** | RYACGTGGYR | ABRE (**ABA responsive element**)" in Arabidopsis | **5** | **CE1** | TGCCACCGG | cis-acting element associated to ABRE, involved in ABA responsiveness |
| **6** | **ABRECE1HVA22** | TGCCACCGG | ABA responsive element | **6** | **EIRE** | TTCGACC | elicitor-responsive element |
| **7** | **ASF1MOTIFCAMV** | TGACG | ASF-1 binding site; ASF-1 binds to two TGACG motifs found in many promoters and are involved in transcriptional activation of several genes by auxin and/or **salicylic acid** | **7** | **ELI-Box** | AAACCAAT | elicitor-responsive element |
| **8** | **BOXLCOREDCPAL** | ACCWWCC | DcMYB1 binding site; DcMYB1 acts as a transcriptional activator of the carrot phenylalanine ammonia-lyase gene in response to elicitor treatment, **UV-B irradiation** and the dilution effect | **8** | **ERE** | ATTTCAAA | ethylene-responsive element |
| **9** | **CURECORECR** | GTAC | Oxygen deficiency responsive gene expression in Chlamydomonas reinhardtii through a copper-sensing signal transduction | **9** | **HSE** | AAAAAATTTC/ AGAAAATTCG/ CNNGAANNTTCNNG | cis-acting element involved in heat-stress |
| **10** | **CRTDREHVCBF2** | GTCGAC | Preferred sequence for AP2 transcriptional activator HvCBF2 of barley; DNA binding is regulated by **temperature** | **10** | **LTR** | CCGAAA | low-temperature responsive element |
| **11** | **CBFHV** | RYCGAC | Binding site of barley CBF1 (**dehydration-responsive element** (DRE) binding proteins | **11** | **MBS** | CAACTG/TAACTG/ CGGTCA | MYB binding site involved in drought-inducibility |
| **12** | **DPBFCOREDCDC3** | ACACNNG | ABA responsive element | **12** | **TCA-element** | CCATCTTTTT/ TCAGAAGAGG/ CAGAAAAGGA | cis-acting element involved in salicylic acid responsiveness |
| **13** | **DRE2COREZMRAB17**  **DRE1COREZMRAB17** | ACCGAC  ACCGAGA | Regulatory elements in vivo in the promoter of the **abscisic acid** responsive gene rab17 from maize | **13** | **TGACG-motif** | TGACG | cis-acting regulatory element involved in the MeJA-responsiveness |
| **14** | **DRECRTCOREAT** | RCCGAC | Core motif of DRE/CRT (dehydration-responsive element/C-repeat) cis-acting element found in many genes in Arabidopsis and in rice; OsDREB genes in rice that function in **drought-, high-salt- and cold**-responsive gene expression. | **14** | **TC-rich repeats*** | ATTTTCTTCA/ ATTTTCTCCA/ GTTTTCTTAC | cis-acting element involved in defense and stress responsiveness |
| **15** | **ERELEE4** | AWTTCAA | An ethylene-responsive enhancer element is involved in the senescence-related expression of the carnation glutathione-S-transferase (GSTI) gene. |  | | | |
| **16** | **ELRECOREPCRP1** | TTGACC | Required for **elicitor responsiveness** |
| **17** | **GCCCORE** | GCCGCC | Core of GCC-box found in many **pathogen-responsive genes** such as PDF1.2, Thi2.1, and PR4; Has been shown to function as **ethylene-responsive element**; |
| **18** | **MYBCORE**  **MYB1AT**  **MYB2AT**  **MYB2CONSENSUSAT** | CNGTTR  WAACCA  TAACTG  YAACKG | MYB recognition site found in the promoters of the **dehydration-responsive** gene rd22 and many other genes in Arabidopsis |
| **19** | **MYCATERD1** | CATGTG | MYC recognition sequence necessary for expression of erd1 (**early responsive to dehydration**) in dehydrated Arabidopsis |
| **20** | **MYCATRD22** | CACATG | Role of Arabidopsis MYC and MYB homologs in **drought- and abscisic acid**-regulated gene expression |
| **21** | **LTRECOREATCOR15** | CCGAC | Core of low temperature responsive element (LTRE) of cor15a gene in Arabidopsis |
| **22** | **LRENPCABE** | ACGTGGCA | Core of **low temperature** responsive element (LTRE) of cor15a gene in Arabidopsis;**ABA** responsiveness |
| **23** | **LTRE1HVBLT49** | CCGAAA | **Low-temperature-responsive element** in barley |
| **24** | **LTREATLTI78** | ACCGACA | Putative **low temperature responsive element** (LTRE) |
| **25** | **PREATPRODH** | ACTCAT | Pro- or hypoosmolarity-responsive element) found in the promoter region of proline dehydrogenase (ProDH) gene in Arabidopsis |
| **26** | **WBOXNTERF3** | TGACY | May be involved in activation of ERF3 gene by **wounding** |
| **27** | **WBOXNTCHN48** | CTGACY | Innvolved in **elicitor-induced activation** of transcription via W box-related cis-acting elements from a basic chitinase gene by WRKY transcription factors in tobacco |
| **28** | **WBOXATNPR1*** | TTGAC | putative functions in response to **environmental stresses** |
| **29** | **WRKY710S*** | TGAC | Early nuclear events in plant **defence signalling** |
| **30** | **GT1CONSENSUS*** | GRWAAW | Influences the level **of SA-inducible** gene expression; |
| **31** | **GT1GMSCAM4*** | GAAAAA | Plays a role in **pathogen- and salt-induced** gene expression |
| **32** | **MYCCONSENSUSAT*** | CANNTG | MYC recognition site found in the promoters of the dehydration-responsive gene rd22 and many other genes in Arabidopsis |
| *Defense-related cis –elements occurring in almost all the promoter regions | | | | | | | |
